# Electronic structures of bent lanthanide(III) complexes with two N-donor ligands†
†Electronic supplementary information (ESI) available. CCDC 1880942–1880946. For ESI and crystallographic data in CIF or other electronic format see DOI: 10.1039/c9sc03431e


**DOI:** 10.1039/c9sc03431e

**Published:** 2019-09-18

**Authors:** Hannah M. Nicholas, Michele Vonci, Conrad A. P. Goodwin, Song Wei Loo, Siobhan R. Murphy, Daniel Cassim, Richard E. P. Winpenny, Eric J. L. McInnes, Nicholas F. Chilton, David P. Mills

**Affiliations:** a Department of Chemistry , School of Natural Sciences , The University of Manchester , Oxford Road , Manchester , M13 9PL , UK . Email: david.mills@manchester.ac.uk

## Abstract

Low coordinate metal complexes can exhibit superlative physicochemical properties, but this chemistry is challenging for the lanthanides (Ln) due to their tendency to maximize electrostatic contacts in predominantly ionic bonding regimes. Although a handful of Ln^2+^ complexes with only two monodentate ligands have been isolated, examples in the most common +3 oxidation state have remained elusive due to the greater electrostatic forces of Ln^3+^ ions. Here, we report bent Ln^3+^ complexes with two bis(silyl)amide ligands; in the solid state the Yb^3+^ analogue exhibits a crystal field similar to its three coordinate precursor rather than that expected for an axial system. This unanticipated finding is in opposition to the predicted electronic structure for two-coordinate systems, indicating that geometries can be more important than the Ln ion identity for dictating the magnetic ground states of low coordinate complexes; this is crucial transferable information for the construction of systems with enhanced magnetic properties.

## Abstract 2

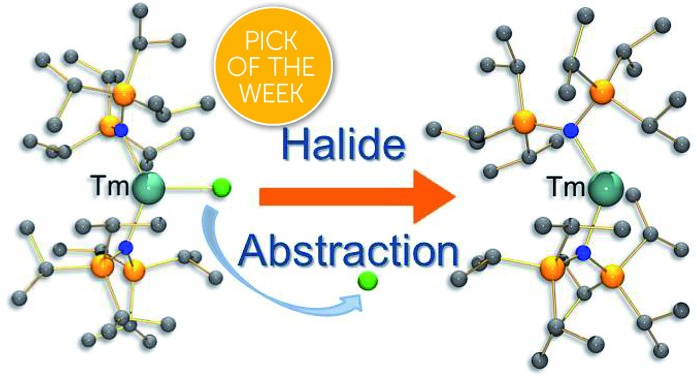
Halide abstraction chemistry is performed on a series of trigonal lanthanide(III) complexes to provide bent complexes that contain only two N-donor ligands.

## Introduction

The remarkable optical, magnetic and catalytic properties of the lanthanides (Ln) have provided numerous technological applications,^1^ and design criteria now exist to build complexes with precise geometrical features that maximize these attributes.^2^–^10^ Highly axial Ln^3+^ complexes have recently become desirable targets for the single-molecule magnet (SMM) community as such geometries can provide maximum anisotropy for several Ln^3+^ ions;^2^–^5^,^11^–^13^ indeed, we have previously predicted that a hypothetical near-linear Dy^3+^ cation [Dy{N(Si^i^Pr_3_)_2_}_2_]^+^ could exhibit a record energy barrier to the reversal of magnetization, providing the inspiration for this work.^14^ Some of us^15^–^18^ and others^19^,^20^ have recently shown that isolated axial Ln^3+^ metallocenium cations [Ln(Cp^R^)_2_]^+^ (Cp^R^ = substituted cyclopentadienyl) can be prepared by halide abstraction from [Ln(Cp^R^)_2_(X)] precursors by using the silylium reagent [H(SiEt_3_)_2_][B(C_6_F_5_)_4_].^21^ The axial [Dy(Cp^R^)_2_]^+^ members of this family^15^,^19^,^20^ together with a linear Tb^2+^ metallocene^22^ exhibit the current highest blocking temperatures for SMMs.

The isolation of low coordinate Ln complexes is often synthetically challenging, as the predominantly ionic bonding regimes in these systems favour high coordination numbers to maximize the number of electrostatic interactions between ligand donor atoms and relatively large Ln cations.^8^ Seminal work by Bradley in the early 1970s provided the trigonal pyramidal Ln complexes, [Ln{N(SiMe_3_)_2_}_3_], which exhibit additional Ln···Cγ–Siβ interactions that stabilize the coordinatively unsaturated Ln^3+^ centres.^23^,^24^ In the interim, numerous trigonal pyramidal and planar Ln^3+^ and Ln^2+^ complexes have been accessed by using a combination of sterically demanding ligands and strict anaerobic conditions.^25^,^26^ In contrast, there are only a handful of structurally characterised monomeric Ln^2+^ complexes with only two formally monodentate ligands; the majority contain intramolecular π-arene contacts,^27^–^31^ whilst bent [Ln{C(SiMe_3_)_3_}_2_] (Ln = Sm, Eu, Yb)^32^–^34^ and near-linear [Ln{N(Si^i^Pr_3_)_2_}_2_] (**1-Ln**; Ln = Sm, Eu, Tm, Yb)^14^,^35^ have additional electrostatic interactions between the ligand σ-bonding frameworks and Ln^2+^ centres. Ln^3+^ complexes with only two monodentate ligands have remained elusive to date as more Lewis acidic Ln^3+^ centres favour higher coordination numbers.^1^

In 2018, some of us showed that **1-Sm** can be easily oxidized by a variety of reagents to afford heteroleptic Sm^3+^ halide complexes [Sm{N(Si^i^Pr_3_)_2_}_2_(X)] (X = F, Cl, Br, I).^36^ Herein we report the synthesis of the bent Ln^3+^ complexes [Ln{N(Si^i^Pr_3_)_2_}_2_][B(C_6_F_5_)_4_] (**2-Ln**; Ln = Sm, Tm, Yb) by an analogous halide abstraction from [Ln{N(Si^i^Pr_3_)_2_}_2_(X)], (**3-Ln**; X = Cl, Ln = Sm,^36^ Tm; X = F, Ln = Yb) using [H(SiEt_3_)_2_][B(C_6_F_5_)_4_]; **3-Tm** and **3-Yb** are prepared by the oxidation of [Ln{N(Si^i^Pr_3_)_2_}_2_] (**1-Ln**; Ln = Tm, Yb) with ^*t*^BuCl and [FeCp_2_][PF_6_], respectively. We have probed the electronic structures of these exotic yet structurally simple complexes by magnetic and EPR methods, supported by *ab initio* calculations. This allows us to probe the effect of approximately linear, bent or planar geometries on the ligand field splitting. Simple electrostatic arguments^5^ based on aspherical electron density distributions in the Russell Saunders sub-levels^37^ predict that **2-Ln** and **3-Ln** should have opposite senses of magnetic anisotropy for a given 4f^*n*^ configuration: we find that this is not always the case, and in fact can vary markedly with the degree of bending of the N–Ln–N angle.

## Results and discussion

### Synthesis

**Scheme 1:**
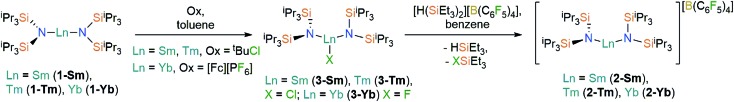
Synthesis of **2-Ln** and **3-Ln**. See ref. 36 for the synthesis of **3-Sm**.

Oxidation of the Ln^2+^ complexes **1-Ln** with either ^*t*^BuCl (Ln = Sm,^36^ Tm) or [FeCp_2_][PF_6_] (Yb) in toluene gave the heteroleptic Ln^3+^ complexes **3-Ln** in good yields (58–72%) following recrystallization from hexane (Scheme 1); similar oxidative procedures on Ln^2+^ bis(silyl)amide complexes have recently been applied by Anwander and co-workers.^38^ The Eu^3+^ analogue **3-Eu** could not be accessed by analogous methods, with crystals of **1-Eu** the only isolable product from numerous attempts to oxidize **1-Eu** with ^*t*^BuCl, [FeCp_2_][PF_6_] and Ph_3_CCl. This can be attributed to the preference of Eu to exhibit the +2 oxidation state over all other Ln, as illustrated by standard reduction potentials, *E*^θ^, Ln^3+^ → Ln^2+^: –0.35 V (Eu), –1.15 V (Yb), –1.55 V (Sm), –2.3 V (Tm).^39^ Halide abstraction of **3-Ln** using [H(SiEt_3_)_2_][B(C_6_F_5_)_4_] in benzene (Sm, Tm) or toluene (Yb) yielded the bent Ln^3+^ complexes, **2-Ln**, in moderate yields (46–70%) after recrystallization from DCM layered with hexane (Scheme 1). The silylium reagent was selected for its solubility in non-coordinating solvents and for the provision of a large thermodynamic driving force for the reaction.^40^

### NMR spectroscopy

The paramagnetic Ln^3+^ centres in **2-Ln** and **3-Ln** engender large pseudocontact shifts and significant signal broadening in NMR spectra;^41^,^42^ the spectra that exhibited signals are compiled in ESI Fig. S4–S13.† ^1^H NMR spectra were recorded from +200 to –200 ppm and for **2-Sm** peaks were observed at 0.43 ppm and –5.27 ppm, corresponding to the methyl and methine protons, respectively, of the bis(silyl)amide ligand. For both **2-Tm** and **2-Yb** only one broad peak was observed at 25.04 ppm and 11.02 ppm, respectively, which we tentatively assign to the methyl protons as these are more numerous than methine protons. No signals were observed for **2-Ln** by ^29^Si{^1^H} and ^13^C{^1^H} NMR spectroscopy. Similarly, no signals were observed for the [B(C_6_F_5_)_4_]^–^ anion in the ^13^C{^1^H} NMR spectra of **2-Ln**; however for **2-Sm**, **2-Tm** and **2-Yb**, the ^11^B{^1^H} NMR spectra displayed sharp peaks at –16.76, –12.35 and –14.67 ppm, respectively. The ^19^F{^1^H} NMR spectra of **2-Sm** and **2-Yb** each displayed three signals characteristic of the [B(C_6_F_5_)_4_]^–^ anion (–133.17, –163.71 and –167.60 ppm for **2-Sm** and –131.58, –162.00 and –165.15 ppm for **2-Yb**), but only one signal was observed in the ^19^F{^1^H} NMR spectrum of **2-Tm** (–128.51 ppm). No signals corresponding to **3-Ln** could be seen in the ^1^H or ^13^C{^1^H} NMR spectra for all **3-Ln**, with only diamagnetic impurities observed; no features were seen in the ^19^F NMR spectrum of **3-Yb**. Given the paucity of information that could be extracted by NMR spectroscopy for **2-Ln** and **3-Ln**, we did not conduct variable temperature studies as these did not prove fruitful for **1-Ln** previously;^35^ instead we have analysed metal–ligand interactions by computational methods (see below).

### Single crystal XRD

**Fig. 1:**
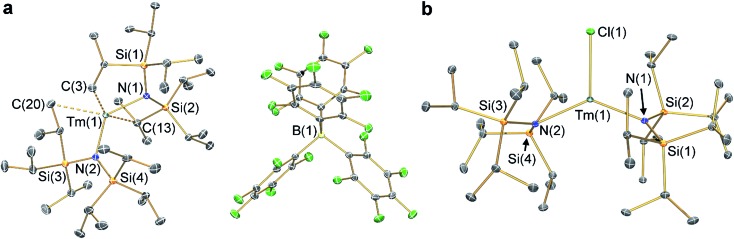
Molecular structures of (a) **2-Tm** and (b) **3-Tm** at 100 K with selected atom labelling. Displacement ellipsoids set at 50% probability level, solvent of crystallization and hydrogen atoms are omitted for clarity. Key: thulium, teal; silicon, orange; nitrogen, blue; fluorine, green; boron, yellow; carbon, grey.

The solid state structures of **2-Ln** and **3-Ln** were determined by single crystal X-ray diffraction. Complexes **2-Tm** and **3-Tm** are depicted in Fig. 1 and selected metrical parameters are compiled in Table 1; see ESI Fig. S1–S3† and ref. 35 for the structures of other complexes. Complexes **2-Ln** are structurally analogous, though **2-Sm** and **2-Yb** both adopt the *P*2_1_/*n* space group and **2-Tm** crystallizes in *P*1[combining macron], and one molecule of DCM was present in the crystal lattice for both **2-Tm** and **2-Yb**, but is absent in crystals of **2-Sm**. The [Ln{N(Si^i^Pr_3_)_2_}_2_]^+^ cations in **2-Ln** exhibit bent geometries defined by the two Ln–N bonds, with N–Ln–N angles of 131.02(8)° for **2-Sm**, 125.49(9)° for **2-Tm**, and 127.7(2)° for **2-Yb**, which are in contrast to the near-linear geometries seen for **1-Ln** (range 166.01(14)–175.5(2)°).^14^,^35^ We attribute the bent geometries of **2-Ln** to the Ln^3+^ cations being more Lewis acidic than the Ln^2+^ centres in **1-Ln**,^14^,^35^ as this permits the more electron deficient Ln^3+^ centres to form additional stabilizing electrostatic contacts with methyl and methine groups of the {N(Si^i^Pr_3_)_2_} ligands. A permanent dipole is formed between the two formally anionic N^–^ centres and Ln^3+^ ion upon bending; such dipolar stabilization mechanisms have previously been used to explain the pyramidal geometries of some f-block tris-silylamides.^43^ Crystal packing forces and inter-ligand dispersion forces also likely make important contributions.^44^ This subtle interplay of forces is particularly apparent for **2-Yb** (see below).

**Table 1:** Selected bond distances and angles of Ln{N(Si^i^Pr_3_)_2_}_2_ moieties in **2-Ln** and **3-Ln**

Complex	Ln–N/Å	N–Ln–N/°	Ln–X
**2-Sm**	2.257(3), 2.228(3)	131.02(8)	—
**2-Tm**	2.156(2), 2.156(2)	125.49(9)	—
**2-Yb**	2.152(4), 2.144(5)	127.7(2)	—
**3-Sm** 36	2.295(2), 2.317(2)	128.24(7)	2.5813(7)
**3-Tm**	2.219(2), 2.238(2)	129.39(5)	2.4832(5)
**3-Yb**	2.226(3), 2.235(3)	138.71(9)	1.983(2)

As with the **1-Ln** series,^14^,^35^ the heavier Ln^3+^ centres in **2-Ln** exhibit more bent N–Ln–N angles, which we again ascribe to the greater charge density of smaller Ln^3+^ cations driving stronger electrostatic interactions with ligand C–H bonds. The {N(Si^i^Pr_3_)_2_} ligands in **2-Ln** are staggered with respect to each other, with the mean Ln–N bond lengths decreasing with Ln^3+^ atomic radii: 2.243(4) Å (Sm), 2.156(3) Å (Tm) and 2.148(6) Å (Yb). It may appear counterintuitive that the Ln–N bonds in **2-Ln** are shorter than those in **1-Ln** (2.483(6) Å, Sm; 2.373(2) Å, Tm; and 2.384(3) Å, Yb)^14^,^35^ given the decreased N–Ln–N angles in **2-Ln** compared with **1-Ln**, but shorter Ln–N bonds for **2-Ln** are expected from an increase in Ln oxidation state. Three Si–C bonds are oriented towards the Ln^3+^ centre in each [Ln{N(Si^i^Pr_3_)_2_}_2_]^+^ cation; these are assigned as Ln···Cγ–Siβ agostic-type interactions by analogy with those discussed for three-coordinate silyl-substituted Ln complexes.^45^–^48^ These interactions lead to three relatively long β-Si–C bonds, three short Ln···Si distances, six Ln···C and six Ln···H electrostatic contacts with methyl/methine groups [*e.g.* for **2-Tm**: range Tm···C: 2.731(3)–3.051(3) Å; range Tm···H: 2.200–2.495 Å; range Tm···Si: 3.066(2)–3.178(2) Å; mean β-Si–C: 1.938(3) Å; range other Si–C: 1.889(3)–1.917(3) Å]. The [B(C_6_F_5_)_4_]^–^ anions do not coordinate; the shortest Ln···F distance for **2-Yb** is 4.627(4) Å, whereas for **2-Sm** and **2-Tm** the shortest Ln···F distances are longer at 7.957(2) Å and 7.715(2) Å, respectively. Using the IUPAC definition of coordination number as the number of metal–ligand σ-bonds,^49^ the cations of **2-Ln** can be considered to be formally two-coordinate as they each exhibit two Ln–N bonds; we probed the numerous additional Ln···Cγ–Siβ electrostatic interactions further through calculations as these could affect the magnetic properties of the proposed [Dy{N(Si^i^Pr_3_)_2_}_2_]^+^ cation (see below).^12^,^14^

The structure of **3-Sm** has previously been reported,^36^ but will be discussed together with **3-Tm** and **3-Yb** as all three complexes are structurally similar. Complex **3-Yb** crystallizes in *P*1[combining macron], whilst **3-Sm** and **3-Tm** are in the *P*2_1_/*c* space group. Complexes **3-Ln** all crystallize with distorted trigonal planar geometries, with the Ln^3+^ centres positioned out of the plane defined by the two nitrogen atoms and halide (distances of Ln from N_2_(X) plane: 0.245(2) Å for **3-Sm**, 0.3292(9) Å for **3-Tm** and 0.312(2) Å for **3-Yb**). As expected the Yb–F bond length of **3-Yb** [1.983(2) Å] is shorter than the Ln–Cl bond lengths of **3-Sm** (2.5813(7) Å) and **3-Tm** (2.4832(5) Å) due to the smaller size of the fluoride anion; this also leads to differing N–Ln–N angles (**3-Sm**: 128.24(7)°; **3-Tm**: 129.39(5)°; **3-Yb**: 138.71(9)°). The mean Sm–N bond length of **3-Sm** (2.306(3) Å) is significantly longer than the mean Ln–N bond lengths of **3-Tm** (2.229(3) Å) and **3-Yb** (2.231(4) Å), which corresponds with earlier Ln^3+^ ions being larger.^1^ The Ln–N bond lengths in **3-Ln** are longer than those in **2-Ln**, as expected from increasing the formal coordination number from two to three. Finally, as with **2-Ln** the coordination spheres of the Ln^3+^ centres of **3-Ln** are completed by multiple electrostatic contacts with methine and methyl groups. These are also likely to arise from Ln···Cγ–Siβ agostic-type interactions, though in **3-Ln** there are fewer, and the Tm···C/H/Si distances are generally longer due to the presence of a halide [*e.g.* for **3-Tm**: range three Tm···C: 2.874(2)–3.261(2) Å; range three Tm···H: 2.324–2.467 Å; range three Tm···Si: 3.195(2)–3.354(2) Å; mean three β-Si–C: 1.928(3) Å; range other Si–C: 1.899(2)–1.913(2) Å].

### UV-vis-NIR spectroscopy

**Fig. 2:**
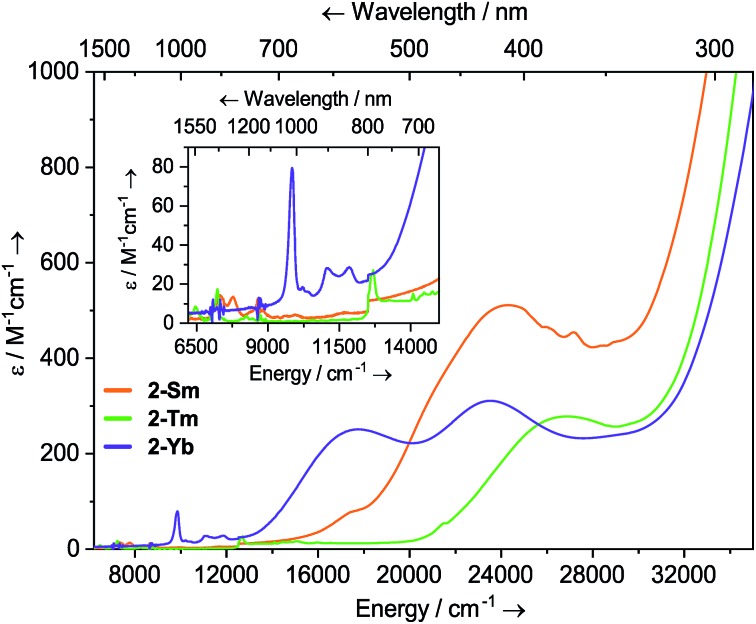
Room temperature UV-vis-NIR spectra of **2-Ln** (1 mM in DCM) from 6200–35 000 cm^–1^.

Dilute solutions of **2-Sm**, **2-Tm** and **2-Yb** in DCM are pale red, green and purple, respectively, and their electronic absorption spectra are dominated by strong ligand to metal charge transfer bands tailing in from the UV region (Fig. 2 and ESI Fig. S19–S21†). Complex **2-Sm** (4f^5^) exhibits the most intense absorption in the visible region [*λ*_max_ = 411 nm (24 300 cm^–1^), *ε* = 511 M^–1^ cm^–1^], whilst **2-Tm** and **2-Yb** exhibit weaker visible absorptions [**2-Tm**; *λ*_max_ = 373 nm (26 800 cm^–1^), *ε* = 275 M^–1^ cm^–1^; **2-Yb**: *λ*_max_ = 425 nm (23 500 cm^–1^), *ε* = 309 M^–1^ cm^–1^, *λ*_max_ = 563 nm (17 800 cm^–1^), *ε* = 249 M^–1^ cm^–1^]. Weak absorptions (*ε* < 100 mol^–1^ dm^3^ cm^–1^) were seen for all **2-Ln** in the near-IR region, corresponding to Laporte-forbidden f–f transitions:^1^**2-Sm** shows absorptions at *λ*_max_ = 1370 nm (7300 cm^–1^), *ε* = 14 M^–1^ cm^–1^ and 1285 nm (7782 cm^–1^), *ε* = 13 M^–1^ cm^–1^, which arise due to ^6^H_5/2_ → ^6^F_J_ transitions; **2-Tm** shows absorptions at *λ*_max_ = 1549 nm (6456 cm^–1^), *ε* = 6 M^–1^ cm^–1^ and *λ*_max_ = 1383 nm (7230 cm^–1^), *ε* = 15 M^–1^ cm^–1^ which arise due to ligand field-split ^3^H_6_ → ^3^H_4_ transitions; **2-Yb** has a broad feature at *λ*_max_ = 1015 nm (9552 cm^–1^), *ε* = 77 M^–1^ cm^–1^ and two weaker absorptions at *λ*_max_ = 904 nm (11 061 cm^–1^), *ε* = 26 M^–1^ cm^–1^ and *λ*_max_ = 844 nm (11 840 cm^–1^), *ε* = 27 M^–1^ cm^–1^ which correspond to ^2^F_7/2_ → ^2^F_5/2_ transitions, showing the ligand field splitting in the excited ^2^F_5/2_ term. These absorptions are moderately strong for f–f transitions because they are all spin-allowed (*ε* < 200 M^–1^ cm^–1^).^1^ The spectral pattern of one intense absorption and two weaker absorptions of approximately equal intensity at higher energy for the ^2^F_7/2_ → ^2^F_5/2_ manifold is a common feature for Yb^3+^ complexes; Da Re *et al.*^50^ and Denning *et al.*^51^ have discussed these transitions in considerable detail previously.

**Fig. 3:**
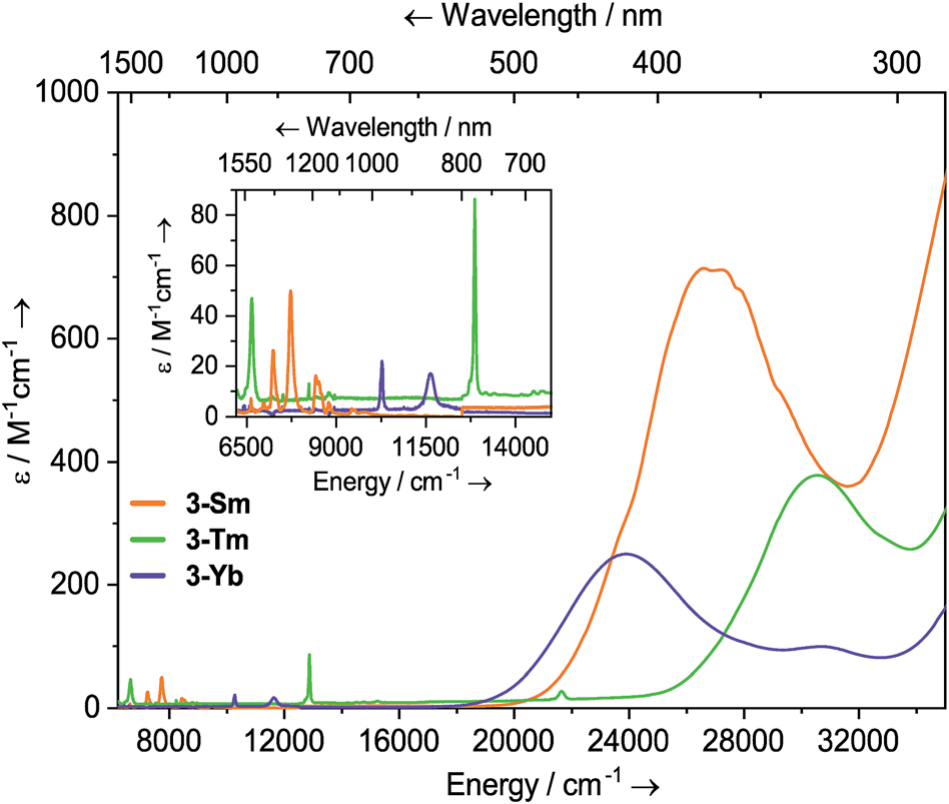
Room temperature UV-vis-NIR spectra of **3-Ln** (1 mM in THF) from 6200–35 000 cm^–1^. For **3-Sm**, an empirical absorption correction of *ε* + 1.9 mol^–1^ dm^3^ cm^–1^ has been applied.

Solutions of **3-Sm**, **3-Tm** and **3-Yb** are pale yellow, green and red, respectively, and as with **2-Ln** their absorption spectra, are dominated by ligand to metal charge transfer bands tailing in from the UV region (Fig. 3, ESI Fig. S22 and S23† and ref. 36) [**3-Sm**: *λ*_max_ = 376 nm (26 595 cm^–1^), *ε* = 713 M^–1^ cm^–1^; **3-Tm**: *λ*_max_ = 327 nm (30 581 cm^–1^), *ε* = 378 M^–1^ cm^–1^; **3-Yb**: *λ*_max_ = 418 nm (23 923 cm^–1^), *ε* = 250 M^–1^ cm^–1^, *λ*_max_ = 326 nm (30 674 cm^–1^), *ε* = 99 M^–1^ cm^–1^]. In the near IR region f–f absorptions are observed for all complexes; **3-Sm** exhibits three main peaks at *ν̃* 7246, 7710 and 8439 cm^–1^ due to ^6^H_5/2_ → ^6^F_J_ transitions, however there appear to be numerous weaker transitions. Complex **3-Tm** shows two main absorptions at *λ*_max_ = 1506 nm (6640 cm^–1^), *ε* = 47 M^–1^ cm^–1^ and *λ*_max_ = 777 nm (12 870 cm^–1^), *ε* = 86 M^–1^ cm^–1^, corresponding to ^3^H_6_ → ^3^H_4_ and ^3^H_6_ → ^3^F_4_ transitions, however again these are structured due to ligand field splitting. Complex **3-Yb** displays two absorptions at *λ*_max_ = 973 nm (10 277 cm^–1^), *ε* = 22 M^–1^ cm^–1^ and *λ*_max_ = 860 nm (11 627 cm^–1^), *ε* = 17 M^–1^ cm^–1^ corresponding to ligand field-split ^2^F_7/2_ → ^2^F_5/2_ transitions. The f–f transitions are at higher energy for **3-Ln**, presumably due to stronger ligand fields; this is most clear for the Yb pair, where for **2-Yb** the lowest energy transition is at 9500 cm^–1^, whilst this is seen at 10 200 cm^–1^ for **3-Yb**.

### Magnetism and EPR spectroscopy

Linear and trigonal-planar environments should stabilize oblate- and prolate-spheroid electron density distributions, respectively, along the axis of quantization.^2^–^5^ This should then stabilize either the minimum or maximum |*m*_*J*_| sublevels of the ^2*S*+1^*L*_*J*_ Russell Saunders ground term depending on the 4f^*n*^ configuration.^2^–^5^ The ions studied here are 4f^5^ (Sm^3+^), 4f^12^ (Tm^3+^) and 4f^13^ (Tm^2+^, Yb^3+^) and in each case the electron density distribution in the maximum |*m*_*J*_| states is prolate, hence an ideal linear geometry at Ln should give the minimum |*m*_*J*_| = ±1/2 (Kramers) or 0 (non-Kramers) ground sublevels, along with easy-plane magnetic anisotropy. Correspondingly, ideal trigonal-planar geometry at Ln should give the maximum |*m*_*J*_| = *J* ground levels and easy-axis magnetic anisotropy. These states can be probed by magnetometry and EPR spectroscopy. Room temperature solution phase magnetic moments (where *χ* is the molar magnetic susceptibility, *T* is the temperature) for **2-Ln** and **3-Ln** determined by the Evans method^52^ are in good agreement with those from solid-state SQUID magnetometry (Table 2 and ESI Fig. S24–S35†). We present the magnetic data for **2-Ln** and **3-Ln** pairs for each Ln^3+^ ion in turn.

**Table 2:** Room temperature *χT* values for **2-Ln** and **3-Ln** determined by Evans solution NMR method and solid-state SQUID magnetometry (1.0 T applied field for **2-Sm** and **3-Sm**; 0.1 T applied field for other compounds), with free-ion values [*g*_*J*_^2^*J*(*J* + 1)/8], and values from CASSCF calculated electronic structures

χ*T*/cm^3^ mol^–1^ K	**2-Sm**	**2-Tm**	**2-Yb**	**3-Sm**	**3-Tm**	**3-Yb**
Free-ion	0.09^*a*^	7.15	2.57	0.09^*a*^	7.15	2.57
Evans	0.43	6.44	2.13	0.38	6.31	1.78
SQUID	0.23	6.86	1.98	0.24	6.31	1.93
CASSCF	0.29	6.88	2.24	0.29	6.85	2.24

^*a*^Theoretical value for ground spin orbit multiplet in the absence of a ligand field.

Complexes **2-Yb** and **3-Yb** have room temperature *χT* values of 1.98 and 1.93 cm^3^ mol^–1^ K, respectively (ESI Fig. S29 and S35†): these are lower than the free-ion 4f^13 2^F_7/2_ value due to substantial crystal field effects, as supported by CASSCF-SO calculations which gives the total spread of the *J* = 7/2 term approaching 2000 cm^–1^ (ESI Table S3†). The same is true for the isoelectronic 4f^13^ Tm^2+^ analogue **1-Tm**.^35^ For **2-Yb** and **3-Yb***χT* decreases slowly on cooling, reaching 1.3 and 1.6 cm^3^ mol^–1^ K, respectively, at 2 K. At 2 K and 7 T, **2-Yb** and **3-Yb** reach saturation magnetizations of 1.80 and 1.84 *μ*_B_, respectively, and the temperature dependence of the traces indicates isolated Kramers doublet ground states as expected (ESI Fig. S28 and S34†).^35^

**Fig. 4:**
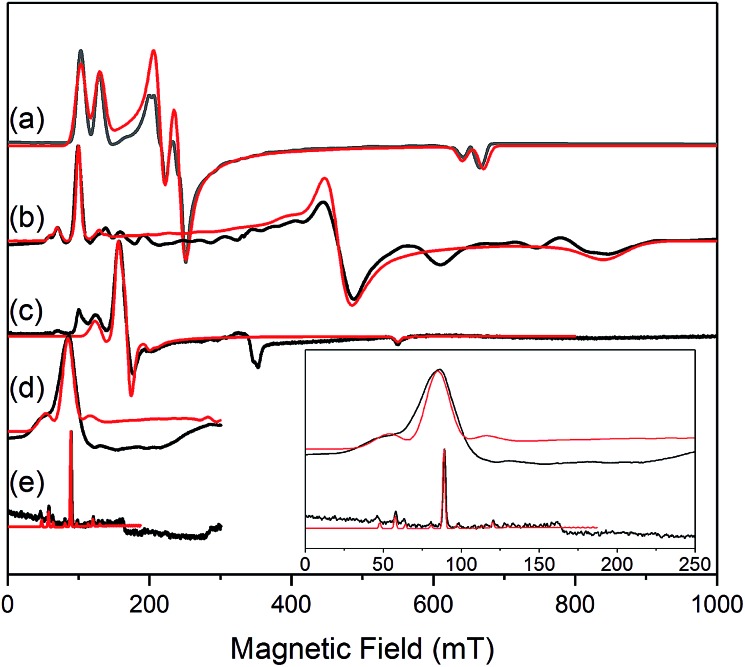
c.w. X-band EPR spectra. (a) **1-Tm** as a powder at 10 K;^35^ (b) **2-Yb** as a powder (in eicosane) at 10 K; (c) **2-Yb** in 1 mM DCM solution at 10 K (the feature at 320 mT is a background signal); (d) **3-Yb** as a powder (in eicosane) at 10 K; (e) **3-Yb** in 1 mM DCM solution at 10 K. Insert shows an expansion of the low field region of (d) and (e); these spectra are truncated as there are no features arising from **3-Yb** at higher fields. Experimental spectra are in black, simulations are in red.

**Fig. 5:**
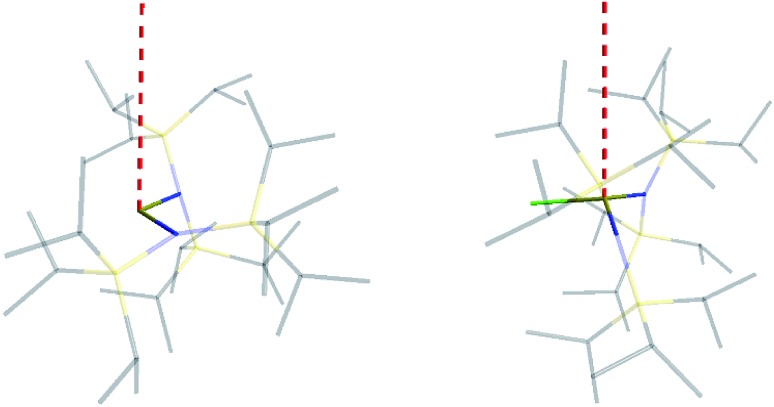
Orientation of the main magnetic axis (red dashed line) for **2-Yb** (left) and **3-Yb** (right).

The similar properties of **2-Yb** and **3-Yb** were confirmed by low-temperature EPR spectroscopy (Fig. 4 and Table 3): solid **2-Yb** has near-axial *g*-values of *g*_1_ = 6.80, *g*_2_ = 1.46 and *g*_3_ = 1.09, whilst solid **3-Yb** gives *g*_1_ = 7.11 with *g*_2,3_ not observed but ≪1. Approximating *g*_1_ = *g*_‖_ and *g*_2,3_ = *g*_⊥_, this *g*_‖_ ≫ *g*_⊥_ pattern clearly demonstrates easy-axis magnetic anisotropy, consistent with a high |*m*_*J*_| ground state doublet (the pure ±7/2 doublet would have *g*_‖_, *g*_⊥_ = 8.0, 0). This is expected for trigonal planar **3-Yb**, but not for **2-Yb** which has only two N-donors that we would expect to stabilize the low |*m*_*J*_| doublet. Hence, for **2-Yb** it appears that the N–Yb–N angle has sufficiently deviated from linearity such that the crystal field is still quantized along the axis normal to the YbN_2_ plane despite the loss of the in-plane fluoride from **3-Yb**. Clearly this result is very different from the easy-plane isoelectronic near-linear Tm^2+^ compound **1-Tm** (Fig. 4a). To further probe this finding, we examined the EPR spectra of the Yb^3+^ compounds in solution. EPR spectra of a frozen solution of **3-Yb** is very similar to the solid state, with *g*_1_ = 7.51 (*g*_2,3_ not observed), however, a frozen solution of **2-Yb** gives *g*_1_ = 4.38, *g*_2_ = 3.99 and *g*_3_ = 1.21 (Fig. 4), which unambiguously shows that there has been a switch to easy-plane anisotropy (now approximate *g*_1,2_ = *g*_⊥_ and *g*_3_ = *g*_‖_) as the *g*_‖_ ≪ *g*_⊥_ pattern indicates stabilization of a low |*m*_*J*_| doublet (the pure ±1/2 doublet would have *g*_‖_, *g*_⊥_ = 1.14, 4.17).^53^ Thus, the structure of **2-Yb** must relax in solution such that the N–Yb–N angle opens up and there is a flip of the orientation of the axis of quantization from being normal to the YbN_2_ plane to lying along the N···N direction. This is supported by CASSCF-SO results based on the crystal structures: these give ground Kramers doublet *g*_1_ = 7.12, *g*_2_ = 1.14 and *g*_3_ = 0.55 for **2-Yb**, and *g*_1_ = 7.90, *g*_2_ = 0.10 and *g*_3_ = 0.07 for **3-Yb** (Table 3), with *g*_1_ (*g*_‖_, defining the axis of quantization) oriented normal to the YbN_2_(F) plane (Fig. 5). The ground doublet is 99% |*m*_*J*_| = 7/2 in character for **3-Yb**, and slightly more mixed at 85% |*m*_*J*_| = 7/2 for **2-Yb** due to the competing components of the crystal field (ESI Table S3†).

**Table 3:** Comparison of EPR data and metrical parameters for isoelectronic **1-Tm**, **2-Yb** and **3-Yb**

Complex	N–Ln–N/°	Calculated *g*-values			Measured *g*-values					
					Solid state			Frozen solution		
		*g*_1_	*g*_2_	*g*_3_	*g*_1_	*g*_2_	*g*_3_	*g*_1_	*g*_2_	*g*_3_
**1-Tm** 35	166.89(6)	5.49	3.60	1.15	5.71	2.92	1.01	5.71	2.92	1.01
**2-Yb**	127.7(2)	7.12	1.14	0.55	6.80	1.46	1.09	4.38	3.99	1.21
**3-Yb**	138.71(9)	7.90	0.10	0.07	7.11	—	—	7.51	—	—

Complex **2-Tm** has a *χT* value of 6.86 cm^3^ mol^–1^ K at 300 K, in good agreement with the free-ion 4f^12 3^H_6_ value. *χT* decreases rapidly with decreasing temperature due to depopulation effects within the multiplet, reaching *ca.* 0.8 cm^3^ K mol^–1^ at 2 K (ESI Fig. S27†). *M*(*H*) curves measured at 2 and 4 K are superimposable and fail to saturate (ESI Fig. S26†), suggesting a singlet non-magnetic ground state for this non-Kramers system. CASSCF-SO calculations, performed on the two crystallographically non-equivalent molecules in the unit cell of **2-Tm**, confirm this, giving a singlet ground state which is separated from the first excited level by *ca.* 14.5 cm^–1^ (average for two independent molecules, ESI Table S3†). Magnetic data for **3-Tm** are markedly different: *χT* (6.31 cm^3^ mol^–1^ K at 300 K) only decreases slowly on cooling, reaching 5.48 cm^3^ mol^–1^ K at 2 K (ESI Fig. S33†), and *M*(*H*) at 2 and 4 K saturate at 3.3 *μ*_B_ above *ca.* 4 T (ESI Fig. S32†); this is direct evidence of a pseudo-doublet magnetic ground state. Indeed, CASSCF-SO calculations give a ground state pseudo-doublet for **3-Tm** with an intra-doublet gap of only 0.13 cm^–1^. The pseudo-doublet wave functions are mixtures of *m*_*J*_ = +6 and –6, which resolve into a pure *m*_*J*_ = +6 and *m*_*J*_ = –6 pair (98% purity) in a small applied magnetic field (ESI Table S5 and S6†). These results are supported by EPR spectroscopy of **2-Tm** and **3-Tm** in the solid state. We find that **2-Tm** is EPR silent at 5 K (ESI Fig. S37†), consistent with the magnetic data and as predicted by CASSCF-SO, whilst **3-Tm** has a near-zero-field EPR transition at X-band (*ca.* 9.39 GHz; ESI Fig. S38†) indicating a zero-field splitting between the pseudo-doublet states of *ca.* 0.3 cm^–1^, in excellent agreement with magnetometry and CASSCF-SO.

For **2-Sm** and **3-Sm** the room temperature *χT* products are 0.23 and 0.24 cm^3^ mol^–1^ K, respectively, higher than the free-ion value for the 4f^5 6^H_5/2_ multiplet (ESI Fig. S25 and S31).† This is indicative of low-lying, thermally accessible excited states as is commonly observed for Sm^3+^ (the ^6^H_7/2_ term lies at only *ca.* 1000 cm^–1^).^54^ On cooling, *χT* steadily decreases to 0.05 and 0.02 cm^3^ mol^–1^ K, respectively, at 2 K. For both **2-Sm** and **3-Sm**, the molar magnetization (*M*) at low temperatures fails to saturate as a function of applied magnetic field (*H*), reaching *ca.* 0.08 and 0.16 *μ*_B_, respectively, at 2 K and 7 T (ESI Fig. S24 and S30†). In both cases, the traces for 2 and 4 K are distinct. These data are consistent with low magnetic moment Kramers doublet ground states. The ^6^H_5/2_ ground term has a low Landé factor of *g*_*J*_ = 2/7, hence the effective *g*-factors for all the Kramers doublets are low. The extreme cases of pure |*m*_*J*_| = 1/2 and 5/2 doublets would have *g*_‖_, *g*_⊥_ = 0.29, 0.86 and 1.43, 0, respectively, and these would give rather similar and low magnetic moments for powders. Unfortunately, we were unable to obtain reliable EPR spectra for **2-Sm** or **3-Sm**. CASSCF-SO calculations give a reasonable agreement with the experimental *χT*(*T*) and *M*(*H*) curves for both **2-Sm** and **3-Sm** (ESI Fig. S24, S25 and S31†) and indicate that the ground state *g*-tensor for **2-Sm** is strongly rhombic, whereas in the case of **3-Sm** the main magnetic axis is perpendicular to the N_2_(Cl) plane with strongly easy-axis character.

Comparing **2-Yb** with isoelectronic **1-Tm**, the N–Ln–N angle in **1-Tm** is much closer to linear at 166.89(6)° [*cf.* 127.7(2)° for **2-Yb**] and it has easy-plane magnetic anisotropy as shown by EPR spectroscopy in both solid and frozen solution state with *g*_1_ = 5.6, *g*_2_ = 3.0 and *g*_3_ = 1.0.^35^ CASSCF-SO calculations for the crystal structure of **1-Tm** give *g*_1_ = 5.49, *g*_2_ = 3.60 and *g*_3_ = 1.15, with *g*_3_ oriented along the N–Tm–N direction, resulting from a 99% pure |*m*_*J*_| = 1/2 ground doublet.^35^ In order to test the importance of the identity of the metal ion *vs.* the N–Ln–N angle, we performed further CASSCF-SO calculations on the structure of **1-Tm** [N–Ln–N 166.89(6)°] where we substitute Yb^3+^ in place of Tm^2+^, and on the structure of **2-Yb** [N–Yb–N 127.7(2)°] where we substitute Tm^2+^ in place of Yb^3+^ (note the change in ion charge to maintain an f^13^ configuration in both cases). We find the former to have an |*m*_*J*_| = 1/2 ground doublet (*g*_1_ = 5.34, *g*_2_ = 3.67, *g*_3_ = 1.16), and the latter to have an |*m*_*J*_| = 7/2 ground doublet (*g*_1_ = 6.76, *g*_2_ = 1.97, *g*_3_ = 0.82): thus, it is the structure that dictates these differing properties for f^13^ configurations and it is not due to the identity of the metal ion. Nocton and co-workers have recently made similar observations for isoelectronic f^13^ Tm^2+^ and Yb^3+^ 18-crown-6 complexes.^55^ Whilst such reasoning is logical, it is not a phenomenon that has been observed frequently with real-world chemical systems.

### *Ab initio* calculations

**Fig. 6:**
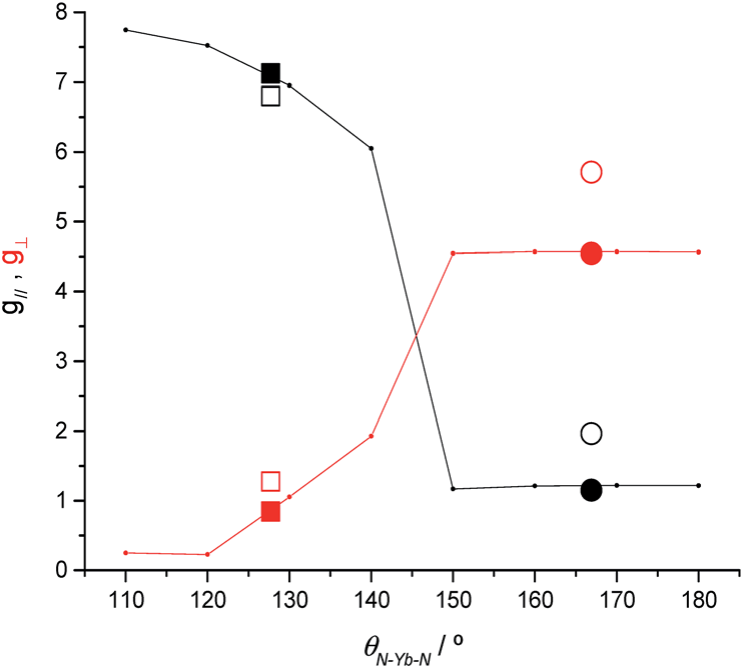
CASSCF-SO calculated *g*_‖_ (black) and *g*_⊥_ (red) for the ground Kramers doublet of model structures based on **2-Yb** as a function of the N–Ln–N angle (lines). CASSCF-SO values based on XRD experimental models (solid symbols) and experimental values (open symbols) for **2-Yb** (squares) and the isoelectronic **1-Tm** (circles). Given the rhombicity of the calculated *g*-tensor we defined *g*_‖_ as the unique value that is either larger or smaller than the average of the three *g*-values, while *g*_⊥_ is defined as the average value of the two remaining *g*-values.

To clarify the dependence of the magnetic anisotropy on the N–Ln–N angle in **2-Yb** we have carried out a systematic *ab initio* investigation. CASSCF-SO calculations have been performed on model structures based on the experimental structure of **2-Yb** in which the N–Ln–N angle has been varied between 180° and 110°. The calculated *g*-values of the ground Kramers doublet of Yb^3+^ show a clear dependence of the type of magnetic anisotropy on the N–Ln–N angle, with the switching point located between 140° and 150° (Fig. 6): easy-axis-like (*g*_1_ > *g*_2,3_; *g*_‖_ > *g*_⊥_) for N–Ln–N angles <140° and easy-plane-like (*g*_3_ < *g*_1,2_; *g*_‖_ < *g*_⊥_) for angles> 150°. This implies that there must be a significant structural change in the N–Yb–N angle of **2-Yb** in the solution phase, becoming at least 150°. Optimization of the structure of **2-Yb** in the gas phase using density-functional theory (DFT) shows an increase in the N–Yb–N angle from 127 to 133° (ESI Table S13†). This indicates that the molecule tends to become more linear when removed from the solid state, suggesting that interactions with solvent molecules (absent in our gas phase calculations) stabilize larger N–Yb–N angles.

We have conducted the same angular-dependent study of the electronic structure of **2-Tm** as for **2-Yb**. The N–Tm–N angle has been varied between 180° and 120° (ESI Fig. S36†). Our results show that there is also a characteristic change in electronic structure for f^12^**2-Tm**: above 160° the singlet ground state is mainly a mixture of *m*_*J*_ = +1 and –1 functions, while below 150° the ground state is dominated by the *m*_*J*_ = 0 function (ESI Fig. S36†). The quantization axis in all cases is the direction that bisects the N–Tm–N angle; given the low symmetry of the complex and the fact that the molecule is neither linear nor trigonal this is not surprising. Therefore, the change in electronic structure from **2-Tm** to **3-Tm** appears to be in agreement with electron density arguments: the trigonal-planar coordination environment of **3-Tm** stabilizes a prolate ground pseudo-doublet with maximum |*m*_*J*_| where the quantization axis is normal to the trigonal plane, whilst the two-coordinate environment of **2-Tm** stabilizes an oblate singlet state dominated by *m*_*J*_ = 0; however in the latter case, far from being linear with a N–Tm–N angle of 125.49(9)°, the axis of quantization bisects the N–Tm–N angle and thus does not follow simple electron density arguments.

To examine the impact of the Ln···Cγ–Siβ agostic-type interactions on the electronic structure of the Yb^3+^ ion in **2-Yb**, we have performed a CASSCF-SO calculation on a model complex where the ligands have been trimmed to {N(SiH_3_)_2_}, thus removing the Ln···C and Ln···H electrostatic contacts; these calculations reveal changes of <10% to the SO energy levels (ESI Table S10†) and a slight increase in axiality of the ground Kramers doublet (ESI Table S11†) compared to **2-Yb**. Although we cannot rule out changes to the N-donor strength for the trimmed ligand *versus* {N(Si^i^Pr_3_)_2_}, these results suggest that the Ln···Cγ–Siβ agostic-type interactions have only a slight influence on the electronic structure at the Yb(iii) site and that they are far weaker than the Ln–N coordination bonds that dominate the electronic structure.

Finally, as this study was driven by our attempts to isolate a near-linear two-coordinate Dy^3+^ complex, it is relevant to predict what the SMM properties of such a material could be now that we are far closer to a representative material with **2-Tm** and **2-Yb**, than in the previously reported near-linear **1-Sm**.^14^ Hence, we have performed CASSCF-SO calculations using the molecular geometry of **2-Tm** where Tm^3+^ has been replaced with Dy^3+^. As predicted based on simple model compounds,^12^ even this bent geometry with a N–Dy–N angle of 125.49(9)° and equatorial agostic interactions can produce a very high barrier to magnetic relaxation of *ca.* 1300–1400 cm^–1^ (ESI Fig. S39 and Table S12†), and thus bent two-coordinate Dy^3+^ complexes of the type presented here are still exciting synthetic targets.

## Conclusions

The preference for bent geometries in [Ln{N(Si^i^Pr_3_)_3_}_2_]^+^ cations can be accredited to the formation of multiple electrostatic contacts between the highly Lewis acidic Ln^3+^ ions and the electron density associated with the ligand σ-bonding network, together with dipole stabilization, crystal packing forces and dispersion force interactions. By a combination of magnetic studies, EPR spectroscopy and *ab initio* calculations we have deduced the electronic structures of the bent Ln^3+^ cations. Interestingly, in the solid state [Yb{N(Si^i^Pr_3_)_3_}_2_]^+^ expresses a similar crystal field to its three-coordinate precursor, rather than the axial crystal field that would be predicted for a two-coordinate complex. EPR spectroscopy shows that [Yb{N(Si^i^Pr_3_)_3_}_2_]^+^ switches to an axial crystal field in solution, indicating that the N–Ln–N angle is less bent in the solution phase. The electronic structures of these bent Ln^3+^ cations are therefore sensitive to changes in molecular geometry.

Our synthetic results show that axial Dy^3+^ complexes such as [Dy{N(Si^i^Pr_3_)_2_}_2_]^+^, proposed as SMMs with large energy magnetization reversal barriers,^14^ are feasible chemical targets, whilst our electronic structure results show that the physical properties of target complexes for the SMM community are not trivially predictable. As a bent [Dy{N(Si^i^Pr_3_)_2_}_2_]^+^ cation is predicted to show a lower effective barrier to magnetic reversal than a linear analogue, it would be of benefit to be able to predict what ligand systems would provide two-coordinate Dy^3+^ complexes that are less bent. Although the [Ln{N(Si^i^Pr_3_)_2_}_2_]^+^ framework is of sufficient steric bulk, a linear geometry is not enforced as the coordination sphere is flexible enough to be rearranged to increase the strength of ligand–metal electrostatic and ligand–ligand London dipole interactions. Given that recently isolated linear Dy^2+^ and Tb^2+^ metallocene systems have been proposed to exhibit significant s–d mixing,^22^ it can be inferenced that combining electronic stabilization with similarly bulky but more rigid ligand frameworks may be a useful strategy in the future pursuit of linear two-coordinate Ln^3+^ complexes.

## Experimental

### Materials and methods

All manipulations were conducted under argon with the strict exclusion of oxygen and water by using Schlenk line and glove box techniques. Toluene, benzene and hexane were dried by refluxing over potassium and were stored over potassium mirrors. Dichloromethane (DCM) was dried over CaH_2_ and was stored over 4 Å molecular sieves. All solvents were degassed before use. For NMR spectroscopy C_6_D_6_ was dried by refluxing over K and CD_2_Cl_2_ was dried by refluxing over CaH_2_. Both NMR solvents were vacuum transferred and degassed by three freeze–pump–thaw cycles before use. **1-Ln**,^14^,^35^ [H(SiEt_3_)_2_][B(C_6_F_5_)_4_]21*a* and **3-Sm**^36^ were prepared according to literature methods.

^1^H (400 MHz), ^13^C{^1^H} (100 MHz and 125 MHz), ^13^C{^19^F} (126 MHz), ^11^B{^1^H} (128 MHz) and ^19^F{^1^H} (376 MHz) NMR spectra were obtained on an Avance III 400 MHz or 500 MHz spectrometer at 298 K. These were referenced to the solvent used, or to external TMS (^1^H, ^13^C), H_3_BO_3_/D_2_O (^11^B) or C_7_H_5_F_3_/CDCl_3_ (^19^F). UV-vis-NIR spectroscopy was performed on samples in Youngs tap-appended 10 mm path length quartz cuvettes on an Agilent Technologies Cary Series UV-vis-NIR Spectrophotometer from 175–3300 nm. FTIR spectra were variously recorded as Nujol mulls in KBr discs on a PerkinElmer Spectrum RX1 spectrometer or as microcrystalline powders using a Bruker Tensor 27 ATR-Fourier Transform Infrared (ATR-FTIR) spectrometer. EPR spectroscopic measurements were performed at X-band using a Bruker super-high-Q X-band resonator attached to a Bruker EMX bridge, on solid state and frozen solution samples contained in flame-sealed quartz EPR tubes. Elemental analysis was carried out by Mr Martin Jennings and Mrs Anne Davies at the Microanalytical service, School of Chemistry, the University of Manchester. Elemental analysis results for **2-Yb** reproducibly gave low carbon values; this has consistently been seen for {N(Si^i^Pr_3_)_2_} complexes and we have previously attributed this observation to the formation of carbides from incomplete combustion.^14^,^35^,^36^,^56^ However, all other analytical data obtained are consistent with the bulk purity of **2-Ln** and **3-Ln**.

#### [Sm{N(Si^i^Pr_3_)_2_}_2_][B(C_6_F_5_)_4_] (**2-Sm**)

Benzene (30 mL) was added to **3-Sm** (0.843 g, 1 mmol) and [H(SiEt_3_)_2_][B(C_6_F_5_)_4_] (0.911 g, 1 mmol) and the resultant orange reaction mixture was stirred overnight at room temperature. The solvent was removed *in vacuo* and the oily red solid was washed with hexane (3 × 20 mL) and dried *in vacuo* for 1 h. The resultant red solid was cooled to –78 °C, dissolved in DCM (5 mL), layered with hexane (10 mL) and stored overnight at –25 °C to yield red crystals of **2-Sm** (1.137 g, 76%). Anal. calcd (%) for C_60_H_84_N_2_Si_4_BF_20_Sm: C, 48.47; H, 5.69; N, 1.88; found: C, 47.25; H, 5.63; N, 1.72. *χT* product (Evans method, 298 K, [D_2_]DCM): 0.43 cm^3^ mol^–1^ K. ^1^H NMR ([D_2_]DCM, 400 MHz, 298 K): *δ* = –5.27 (br, 72H, *v*_1/2_ ∼ 10 Hz, C*H*(CH_3_)_2_), 0.43 (br, 12H, *v*_1/2_ ∼ 50 Hz, CH(C*H*_3_)_2_). ^11^B{^1^H} NMR ([D_2_]DCM, 128 MHz, 298 K): *δ* = –16.76 (s). ^19^F NMR ([D_2_]DCM, 376 MHz, 298 K): *δ* = –133.17 (br, *o*-F), –163.71 (br, *p*-F), –167.60 (br, *m*-F). The paramagnetism of **2-Sm** precluded assignment of its ^13^C{^1^H} and ^29^Si NMR spectra. IR (ATR, microcrystalline): 2954 (s), 2870 (s), 2813 (s), 1642 (s), 1511 (s), 1459 (s), 1384 (m), 1273 (s), 1082 (s), 978 (s), 928 (s), 881 (s), 765 (m), 693 (s), 676 (m), 543 (s), 489 (s), 415 (s) cm^–1^.

#### [Tm{N(Si^i^Pr_3_)_2_}_2_][B(C_6_F_5_)_4_] (**2-Tm**)

Benzene (30 mL) was added to **3-Tm** (1.905 g, 2.21 mmol) and [H(SiEt_3_)_2_][B(C_6_F_5_)_4_] (2.012 g, 2.21 mmol) and the resultant yellow reaction mixture was stirred overnight at room temperature. The solvent was removed *in vacuo* and the oily yellow-green solid was washed with hexane (3 × 20 mL) and dried *in vacuo* for 1 h. The resultant yellow-green solid was cooled to –78 °C, dissolved in DCM (5 mL), layered with hexane (10 mL) and stored overnight at –25 °C to yield yellow-green crystals of **2-Tm** (1.540 g, 46%). Anal. calcd (%) for C_60_H_84_N_2_Si_4_BF_20_Tm: C, 46.06; H, 5.45; N, 1.76; found: C, 46.01; H, 5.55; N, 1.70. *χT* product (Evans method, 298 K, [D_2_]DCM): 6.44 cm^3^ mol^–1^ K. ^1^H NMR ([D_2_]DCM, 400 MHz, 298 K): *δ* = 25.04 (br, *v*_1/2_–800 Hz, CH(C*H*_3_)_2_). ^11^B{^1^H} NMR ([D_2_]DCM, 128 MHz, 298 K): *δ* = –12.39 (s). ^19^F NMR ([D_2_]DCM, 376 MHz, 298 K): *δ* = –128.51 (br, *o*-F). The paramagnetism of **2-Tm** precluded assignment of its ^13^C{^1^H} and ^29^Si NMR spectra. IR (Nujol): 2359 (m), 2340 (m), 1643 (w), 1514 (m), 980 (m), 918 (w), 897 (w), 800 (w), 773 (w), 756 (w), 700 (w), 683 (w), 667 (w), 660 (w) cm^–1^.

#### [Yb{N(Si^i^Pr_3_)_2_}_2_][B(C_6_F_5_)_4_] (**2-Yb**)

Toluene (15 mL) was added to a pre-cooled (–78 °C) mixture of **3-Yb** (0.425 g, 0.5 mmol) and [H(SiEt_3_)_2_][B(C_6_F_5_)_4_] (0.455 g, 0.5 mmol). The resultant dark purple reaction mixture was allowed to warm to room temperature slowly and stirred overnight. The solvent was removed *in vacuo* and the oily dark purple solid was washed with hexane (3 × 20 mL) and dried *in vacuo* for 1 h. The resultant dark purple solid was cooled to –78 °C, dissolved in DCM (1.5 mL), layered with hexane (3 mL) and stored at –35 °C overnight to yield dark purple crystals of **2-Yb** (0.5272 g, 70%). Anal. calcd (%) C_60_H_84_N_2_Si_4_F_20_BYb·CH_2_Cl_2_: C, 45.94; H, 5.44; N, 1.76; found: C, 44.81; H, 5.18; N, 1.58. *χT* product (Evans method, 298 K, [D_2_]DCM): 2.13 cm^3^ mol^–1^ K. ^1^H NMR ([D_2_]DCM, 400 MHz, 298 K): *δ* = 11.02 (br, *v*_1/2_ ∼ 400 Hz, CH(C*H*_3_)_2_). ^11^B{^1^H} NMR ([D_2_]DCM, 128 MHz, 298 K): *δ* = –14.67 (s). ([D_2_]DCM, 376 MHz, 298 K): *δ* = –131.58 (br, *o*-F), –162.05 (br, *p*-F), –165.15 (br, *m*-F). The paramagnetism of **2-Yb** precluded assignment of its ^13^C{^1^H} and ^29^Si NMR spectra. IR (Nujol): 1267 (w), 1086 (m), 980 (m), 945 (w), 885 (w), 800 (w), 704 (m), 660 (m) cm^–1^.

#### [Tm{N(Si^i^Pr_3_)_2_}_2_(Cl)] (**3-Tm**)

A solution of ^*t*^BuCl (0.82 mL, 7.5 mmol) in toluene (10 mL) was added dropwise to a pre-cooled (–78 °C) solution of **1-Tm** (1.240 g, 1.5 mmol). The reaction mixture was allowed to warm slowly to room temperature and was stirred at room temperature for 30 min, resulting in a colour change from dark brown to light brown. Volatiles were removed *in vacuo* and the product was extracted with hexane (10 mL), filtered, concentrated to 7 mL and stored at –35 °C overnight to yield pale green crystals of **3-Tm** (0.930 g, 72%). Anal. calcd (%) C_36_H_84_N_2_Si_4_ClTm: C, 50.17; H, 9.82; N, 3.25; found: C, 50.39; H, 10.23; N, 4.11. *χT* product (Evans method, 298 K, [D_6_]benzene): 6.31 cm^3^ mol^–1^ K. The paramagnetism of **3-Tm** precluded assignment of its ^1^H, ^13^C{^1^H} and ^29^Si NMR spectra. IR (Nujol): 1260 (w), 1245 (w), 1077 (w), 1061 (w), 1012 (m), 991 (w), 934 (s), 879 (m), 799 (w), 728 (m), 701 (s), 667 (m), 632 (m), 598 (m) cm^–1^.

#### [Yb{N(Si^i^Pr_3_)_2_}_2_(F)] (**3-Yb**)

Toluene (20 mL) was added to a pre-cooled (–78 °C) mixture of **1-Yb** (1.246 g, 1.5 mmol) and [Fe(Cp)_2_][PF_6_] (0.496 g, 1.5 mmol) with stirring, and a white vapour was observed. The orange reaction mixture was stirred overnight at room temperature. All volatiles were removed *in vacuo* and ferrocene was sublimed away from the crude product at 90 °C for 1.5 hours. The remaining crude orange powder (1.029 g) was extracted with hexane (10 mL), filtered, concentrated to 7 mL and stored at –35 °C overnight to yield orange-red crystals of **3-Yb** (0.734 g, 58%). Anal. calcd (%) C_36_H_84_N_2_Si_4_FYb·0.8C_6_H_14_: C, 53.36; H, 10.45; N, 3.05; found: C, 53.92; H, 10.87; N, 3.73. *χT* product (Evans method, 298 K, [D_6_]benzene): 1.78 cm^3^ mol^–1^ K. The paramagnetism of **3-Yb** precluded assignment of its ^1^H, ^13^C{^1^H}, ^19^F and ^29^Si NMR spectra. IR (Nujol): 1247 (w), 1214 (w), 1071 (w), 1012 (w), 996 (w), 944 (m), 882 (m), 800 (w), 703 (m), 665 (m) cm^–1^.

## Conflicts of interest

There are no conflicts to declare.

## Supplementary Material

Supplementary information

Video abstract

Crystal structure data
