# Supplementary material for: Electronic structures of bent lanthanide(III) complexes with two N-donor ligands
Source: Chem Sci. 2019 Sep 18;10(45):10493–502. doi: 10.1039/c9sc03431e (PMC7020784; doi:10.1039/c9sc03431e)
Supplement: Supplementary file 1 [file SC-010-C9SC03431E-s001.pdf]

Electronic Supplementary Material (ESI) for Chemical Science.
This journal is © The Royal Society of Chemistry 2019

Supplementary Information for:

Electronic Structures of Bent Lanthanide(III) Complexes With Two N-Donor Ligands

Hannah M. Nicholas, Michele Vonci, Conrad A. P. Goodwin, Song Wei Loo, Siobhan R. Murphy, Daniel Cassim, Richard E. P. Winpenny, Eric J. L. McInnes, Nicholas F. Chilton and David P. Mills*

School of Chemistry, The University of Manchester, Oxford Road, Manchester, M13 9PL, U.K.

Contents

1. Crystallography **S2**

2. Molecular structures of complexes 2-Sm, 2-Yb and 3-Yb **S5**

3. NMR spectroscopy **S8**

4. ATR-IR spectroscopy **S13**

5. UV-vis-NIR spectroscopy **S16**

6. Magnetism, EPR spectroscopy and CASSCF-SO electronic structure **S19**

7. DFT optimisation **S40**

8. References **S44**

1. Crystallography

The crystal data for complexes **2-Ln** (Ln = Sm, **2-Sm**; Tm, **2-Tm**; Yb, **2-Yb**), **3-Ln** (Ln = Tm, **3-Tm**; Yb, **3-Yb**) are compiled in Tables S1 and S2. Crystals of **2-Sm**, **2-Tm**, **3-Tm** and **3-Yb** were examined using a Rigaku XtalLAB AFC11 diffractometer with a CCD area detector and a graphite-monochromated Cu Kα (λ = 1.54178 Å) or Mo Kα radiation (λ = 0.71073 Å). Crystals of **2-Yb** were examined using a Bruker Apex II diffractometer with a CCD area detector and a graphite-monochromated Cu Kα radiation (λ = 1.54184 Å). Intensities were integrated from data recorded on 0.5° (**2-Tm**, **2-Yb** and **3-Yb**) or 1° (**2-Sm** and **3-Tm**) frames by ω rotation or ω and ϕ rotation in the case of **2-Yb**. Cell parameters were refined from the observed positions of all strong reflections in each data set. A Gaussian grid face-indexed (**2-Sm**, **2-Tm**, **3-Tm** and **3-Yb**), or multi-scan (**2-Yb**) absorption correction with a beam profile was applied.[1] The structures were solved using SHELXL (**2-Sm**, **2-Tm**, **2-Yb**, **3-Tm** and **3-Yb**)[2] and the datasets were refined by full-matrix least-squares on all unique F^2 values,[3] with anisotropic displacement parameters for all non-hydrogen atoms, and with constrained riding hydrogen geometries; U_{iso}(H) was set at 1.2 (1.5 for methyl groups) times U_{eq} of the parent atom. The largest features in final difference syntheses were close to heavy atoms and were of no chemical significance. CrysAlisPro[1] was used for control and integration, and SHELX[3,5] was employed through OLEX2[6] for structure solution and refinement. ORTEP-3[5] and POV-Ray[3] were employed for molecular graphics. CCDC 1880942–1880946 contain the supplementary crystal data for this article. These data can be obtained free of charge from the Cambridge Crystallographic Data Centre via www.ccdc.cam.ac.uk/data_request/cif.

Table S1. Crystallographic data for **2-Sm**, **2-Tm**, **2-Yb**.

	**2-Sm**	**2-Tm**	**2-Yb**
Empirical formula	$C_{60}H_{84}BN_2F_{20}Si_4Sm$	$C_{61}H_{86}BCl_2F_{20}N_2Si_4Tm$	$C_{61}H_{86}BCl_2F_{20}N_2Si_4Yb$
Formula weight	1486.81	1590.31	1594.42
Temperature/K	100.0(2)	293(2)	150.0(2)
Crystal system	monoclinic	triclinic	monoclinic
Space group	$P2_1/n$	P-1	$P2_1/n$
a/Å	19.9284(3)	16.3084(3)	16.0620(3)
b/Å	17.0513(2)	16.8020(4)	25.9262(6)
c/Å	21.1049(4)	25.4751(5)	16.7464(3)
α/°	90	89.3578(18)	90
β/°	114.818(2)	88.4783(16)	92.749(2)
γ/°	90	88.0167(16)	90
Volume/$Å^3$	6509.2(2)	6973.5(2)	6965.6(2)
Z	4	4	4
$\rho_{calc}g/cm^3$	1.517	1.515	1.520
μ/mm^{-1}	8.338	1.510	4.666
F(000)	3044.0	3240.0	3244.0
Crystal size/mm^3	0.45 × 0.2 × 0.17	0.547 × 0.351 × 0.275	0.3 × 0.1 × 0.1
Radiation	CuKα (λ = 1.54184)	MoKα (λ = 0.71073)	CuKα (λ = 1.54184)
2Θ range for data collection/°	5.122 to 136.502	4.746 to 50.7	6.288 to 136.494
Index ranges	$-23 \le h \le 24, -18 \le k \le 20, -25 \le l \le 25$	$-19 \le h \le 19, -20 \le k \le 20, -30 \le l \le 30$	$-19 \le h \le 19, -31 \le k \le 31, -20 \le l \le 20$
Reflections collected	71543	69827	28972
Independent reflections	11897 [R_{int} = 0.0790, R_{sigma} = 0.0373]	25354 [R_{int} = 0.0300, R_{sigma} = 0.0373]	12492 [R_{int} = 0.0732, R_{sigma} = 0.1021]
Data/restraints/parameters	11897/0/817	25354/1593/1697	12492/30/844
Goodness-of-fit on F^2	1.052	1.049	1.007
Final R indexes [I>=2σ (I)]	R_1 = 0.0361, wR_2 = 0.0919	R_1 = 0.0305, wR_2 = 0.0678	R_1 = 0.0560, wR_2 = 0.1146
Final R indexes [all data]	R_1 = 0.0405, wR_2 = 0.0944	R_1 = 0.0377, wR_2 = 0.0697	R_1 = 0.1003, wR_2 = 0.1337
Largest diff. peak/hole / e $Å^{-3}$	0.64/-1.34	1.36/-0.55	0.79/-0.78

Table S2. Crystallographic data for **3-Tm** and **3-Yb**.

	**3-Tm**	**3-Yb**
Empirical formula	$C_{36}H_{84}ClN_2Si_4Tm$	$C_{36}H_{84}FN_2Si_4Yb$
Formula weight	861.79	849.45
Temperature/K	100.00(2)	150(2)
Crystal system	monoclinic	triclinic
Space group	$P2_1/c$	P-1
a/Å	15.8309(4)	8.7293(3)
b/Å	13.1077(3)	11.1712(4)
c/Å	22.5379(6)	24.4919(8)
α/°	90	84.182(3)
β/°	109.043(3)	80.590(3)
γ/°	90	68.517(3)
Volume/$Å^3$	4420.8(2)	2190.28(13)
Z	4	2
$\rho_{calc}g/cm^3$	1.295	1.288
μ/mm^{-1}	2.201	5.206
F(000)	1824.0	898.0
Crystal size/mm^3	0.435 × 0.301 × 0.217	0.2 × 0.1 × 0.05
Radiation	MoKα (λ = 0.71073)	CuKα (λ = 1.54184)
2Θ range for data collection/°	7.056 to 50.692	3.66 to 136.502
Index ranges	$-18 \leq h \leq 19, -15 \leq k \leq 15, -27 \leq l \leq 27$	$-10 \leq h \leq 10, -13 \leq k \leq 12, -29 \leq l \leq 29$
Reflections collected	31900	17168
Independent reflections	8039 [R_{int} = 0.0260, R_{sigma} = 0.0227]	7685 [Rint = 0.0349, Rsigma = 0.0302]
Data/restraints/parameters	8039/0/421	7685/0/421
Goodness-of-fit on F^2	1.029	1.047
Final R indexes [I>=2σ (I)]	R_1 = 0.0185, wR_2 = 0.0468	R1 = 0.0355, wR2 = 0.0925
Final R indexes [all data]	R_1 = 0.0210, wR_2 = 0.0476	R1 = 0.0376, wR2 = 0.0937
Largest diff. peak/hole / e $Å^{-3}$	0.59/-0.60	1.49/-1.43

2. Molecular structures of complexes 2-Sm, 2-Yb and 3-Yb.

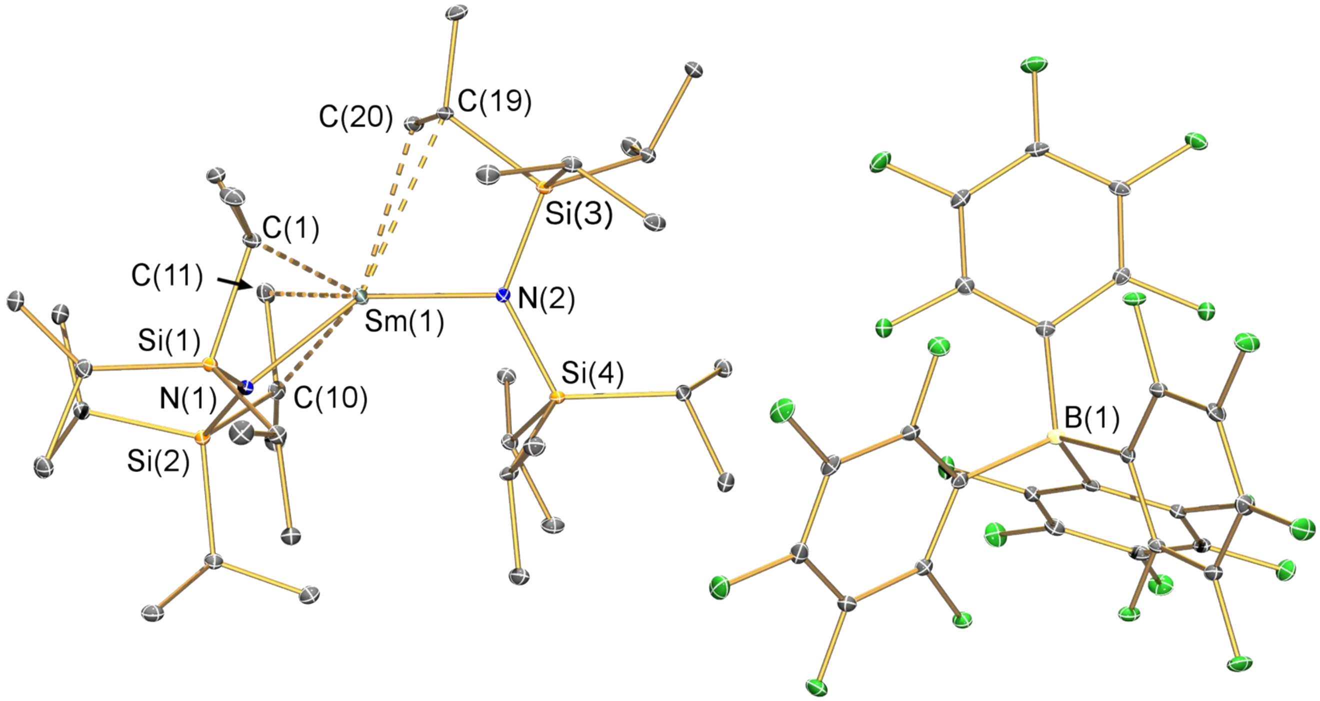

Figure S1. Molecular structure of **2-Sm** with selected atom labelling. Displacement ellipsoids set at 50% probability level, solvent of crystallization and hydrogen atoms are omitted for clarity. C atoms are grey and F atoms are green. Selected distances and angles: Sm(1)–N(1), 2.25667(3) Å; Sm(1)–N(2), 2.22817(4) Å; Sm(1)···C(1), 2.94148(4) Å; Sm(1)···C(10), 2.99550(5) Å; Sm(1)···C(11), 3.00204(9) Å; Sm(1)···C(19), 3.06311(3) Å; Sm(1)···C(20), 2.90931(4) Å; N(1)–Sm(1)–N(2),131.021(2)°.

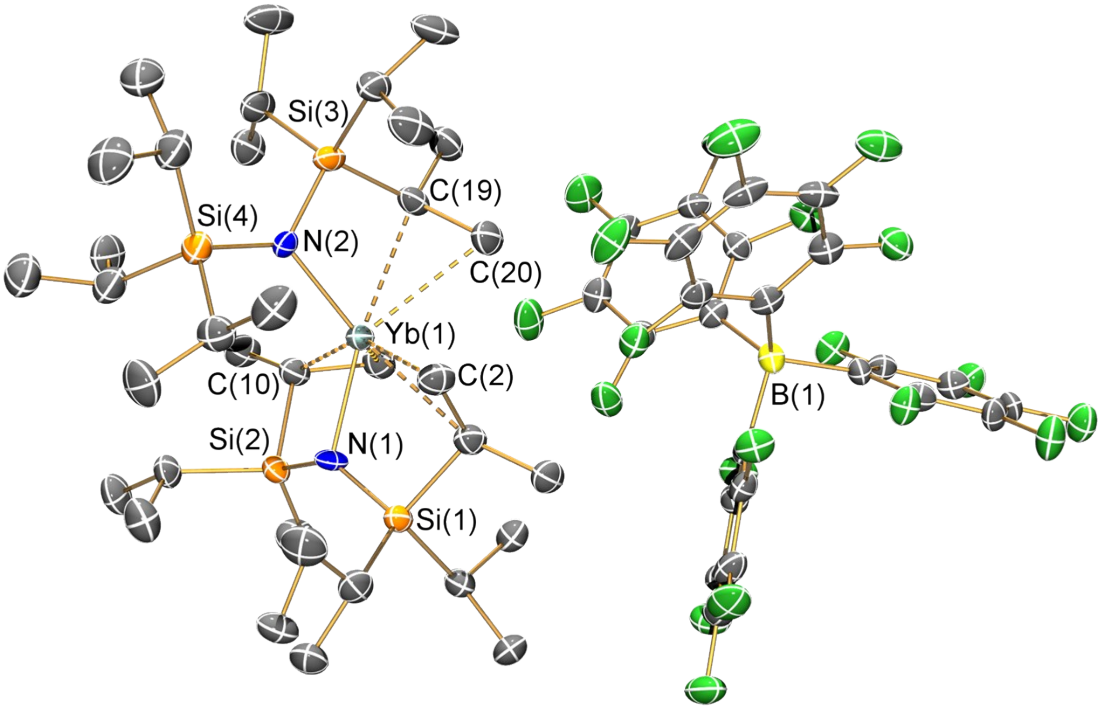

Figure S2. Molecular structure of **2-Yb** with selected atom labelling. Displacement ellipsoids set at 50% probability level, solvent of crystallization and hydrogen atoms are omitted for clarity. C atoms are grey and F atoms are green. Selected distances and angles: Yb(1)–N(1), 2.15164(3) Å; Yb (1)–N(2), 2.14416(4) Å; Yb(1)···C(2), 2.80753(4) Å; Yb(1)···C(10), 2.87076(5) Å; Yb(1)···C(19), 2.69789(5) Å; Yb(1)···C(20), 2.75446(4) Å; N(1)–Yb(1)–N(2), 127.6666(11)°.

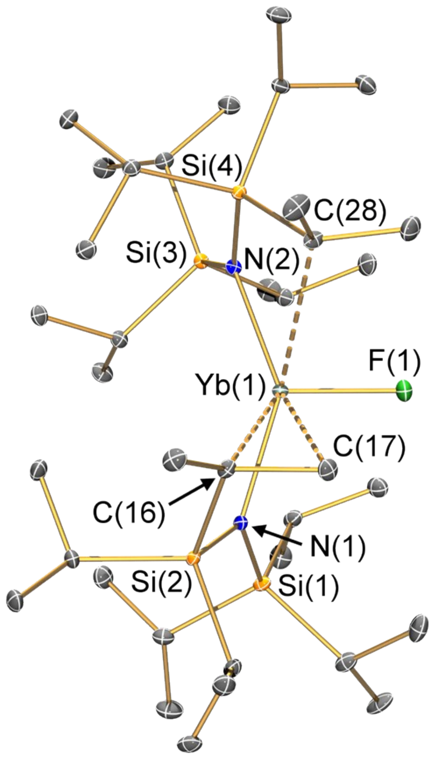

Figure S3. Molecular structure of **3-Yb** with selected atom labelling. Displacement ellipsoids set at 50% probability level, solvent of crystallization and hydrogen atoms are omitted for clarity. C atoms are gray. Selected distances and angles: Yb(1)–N(1), 2.22565(8) Å; Yb(1)–N(2), 2.23538(7) Å; Yb(1)···C(16), 2.93449(10) Å; Yb(1)···C(17), 3.13443(12) Å; Yb(1)···C(28), 3.12546(13) Å; Yb(1)–F(1), 1.9875(6) Å; N(1)– Yb(1)–N(2), 138.712(2)°.

3. NMR Spectroscopy

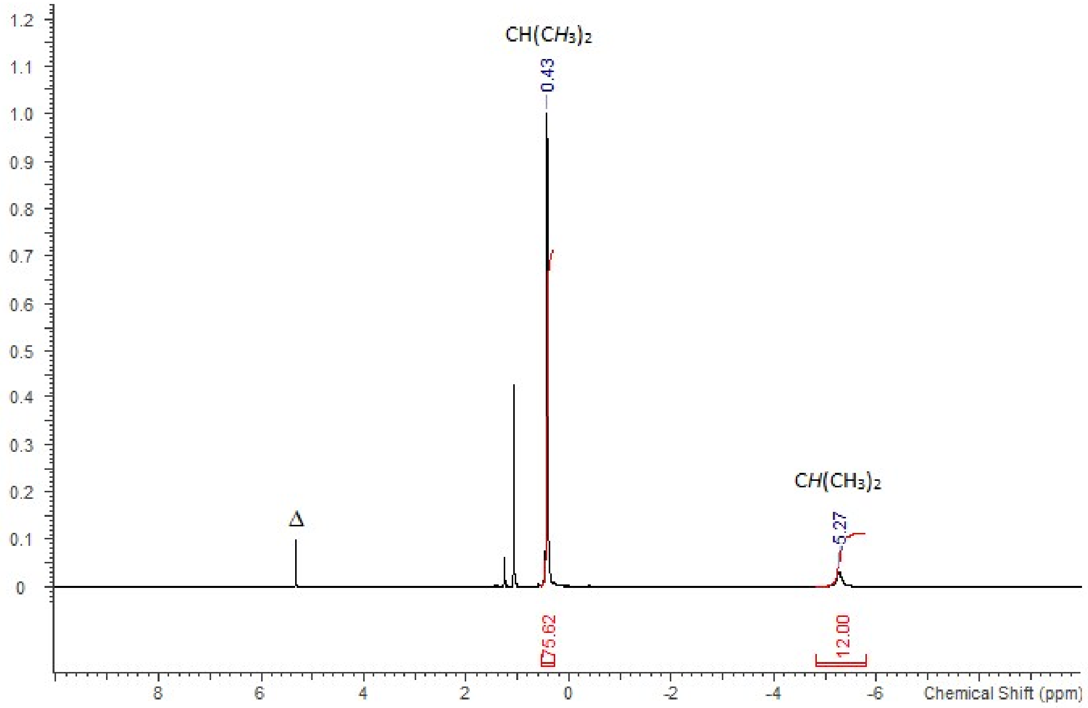

Figure S4. ^{1}H NMR spectrum (swept from +200 to –200 ppm) of **2-Sm** in CD_2Cl_2, zoomed in the region +10 to –10 ppm. Δ denotes solvent residual. Diamagnetic impurities between 1 and 1.5 ppm.

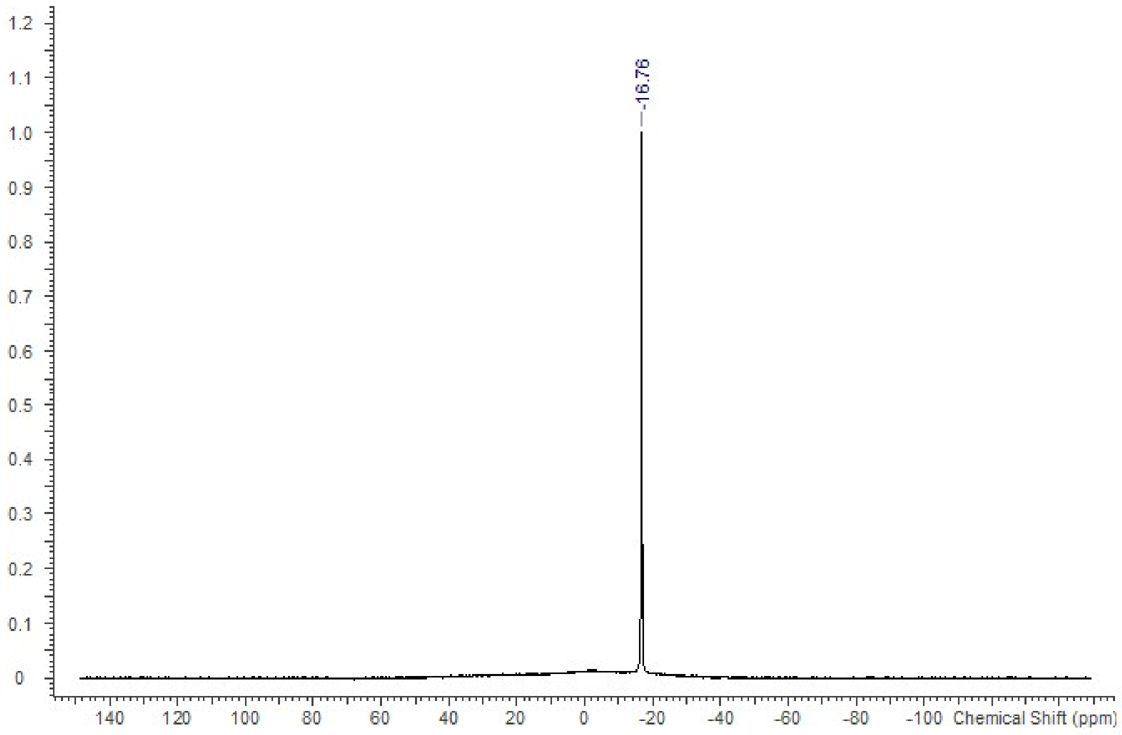

Figure S5. ^{11}B NMR spectrum of **2-Sm** in CD_2Cl_2.

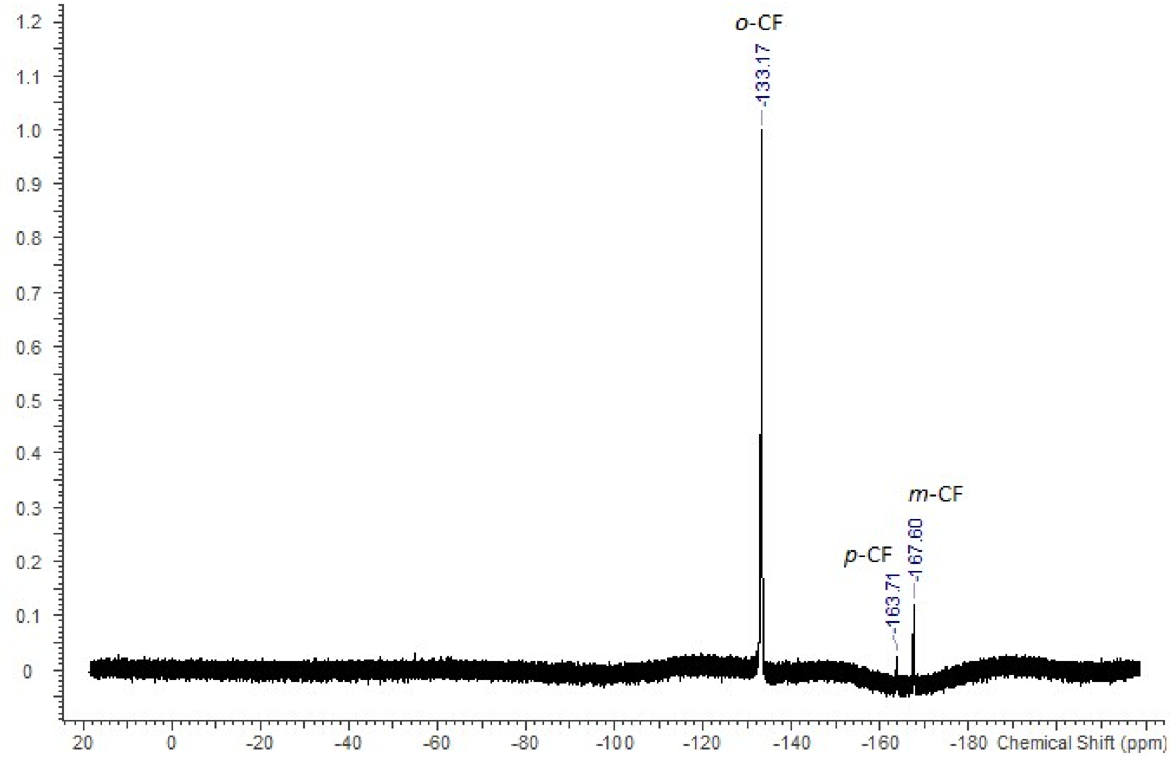

Figure S6. ^{19}F NMR spectrum of **2-Sm** in CD_2Cl_2.

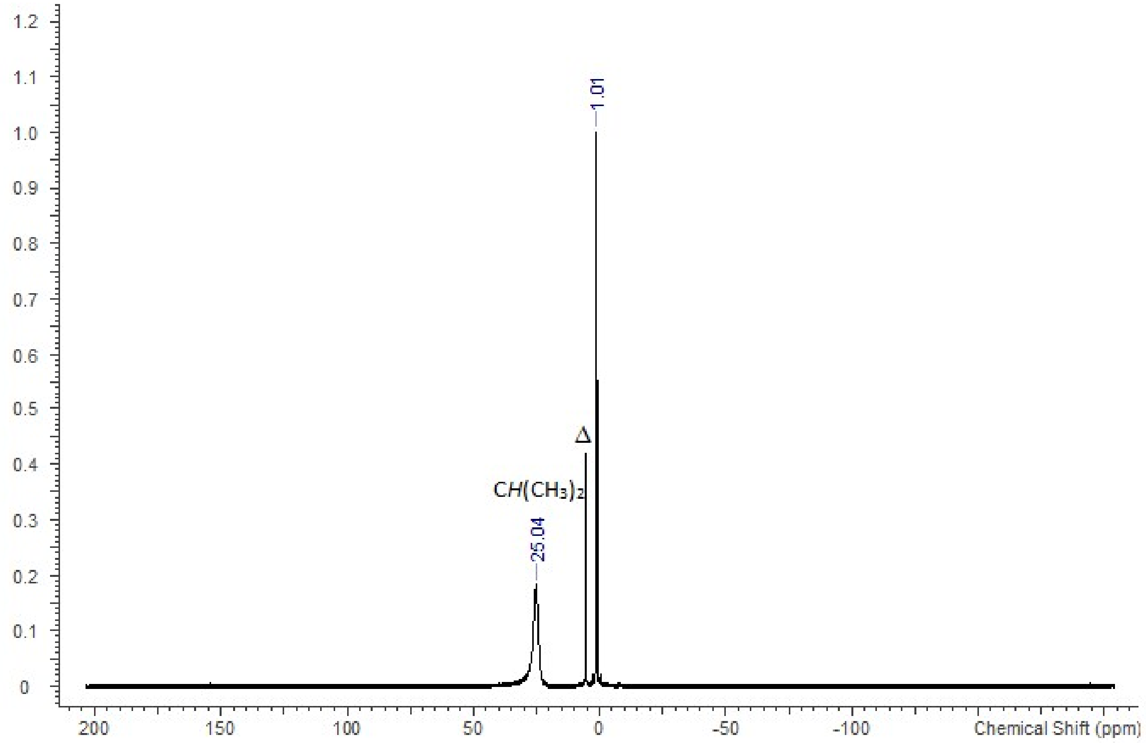

Figure S7. ^{1}H NMR spectrum (swept from +200 to –200 ppm) of **2-Tm** in CD_2Cl_2. Δ denotes solvent residual. Signal at 1.01 ppm assigned to trace $HN(Si^iPr_3)_2$ impurity.

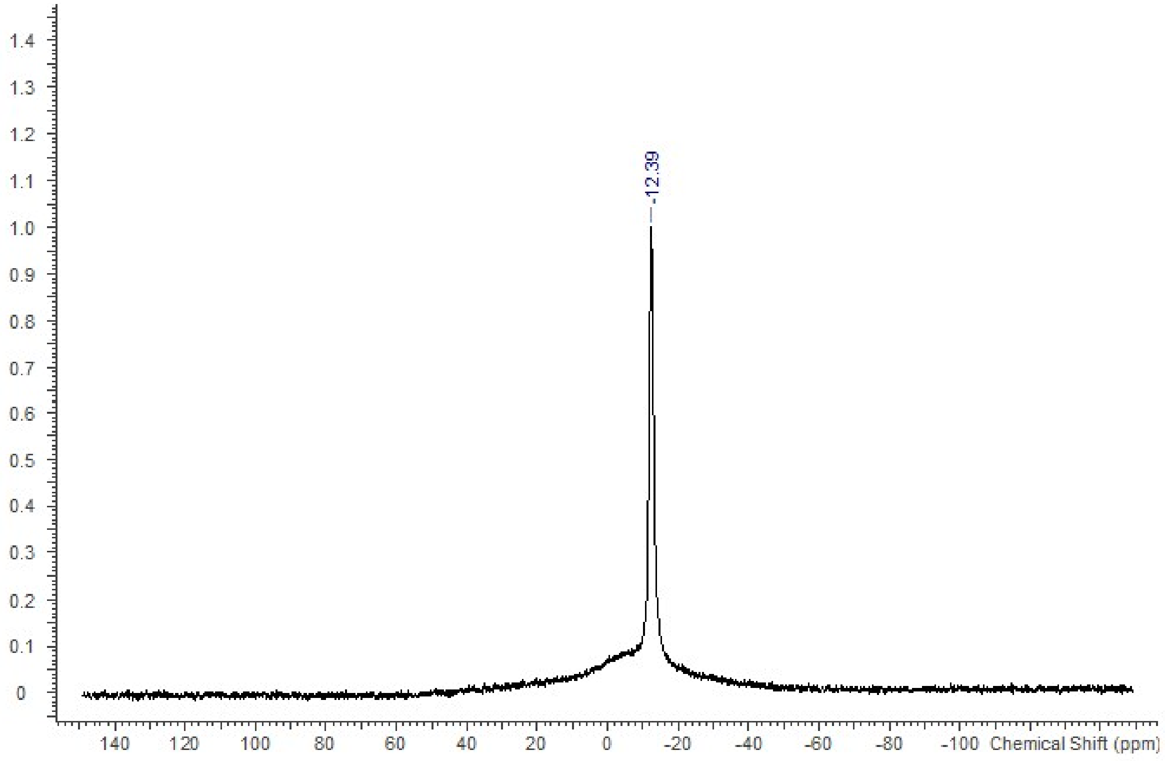

Figure S8. ^{11}B NMR spectrum of **2-Tm** in CD_2Cl_2.

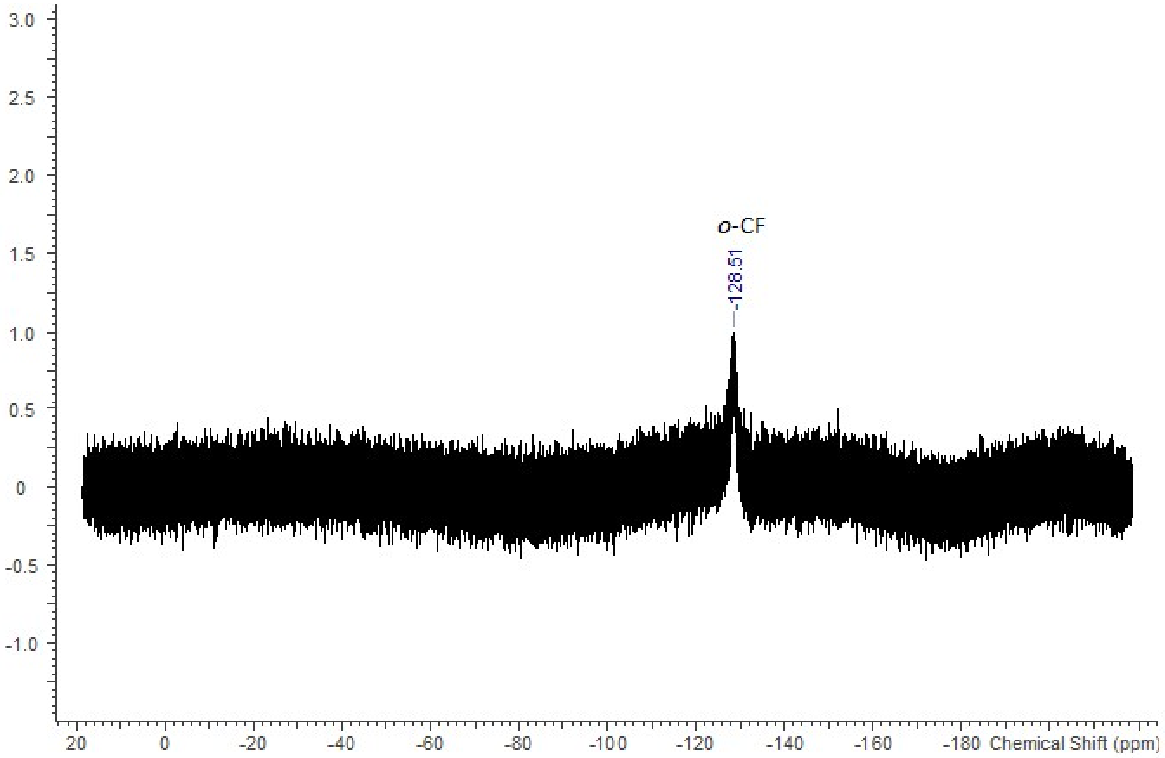

Figure S9. ^{19}F NMR spectrum of **2-Tm** in CD_2Cl_2.

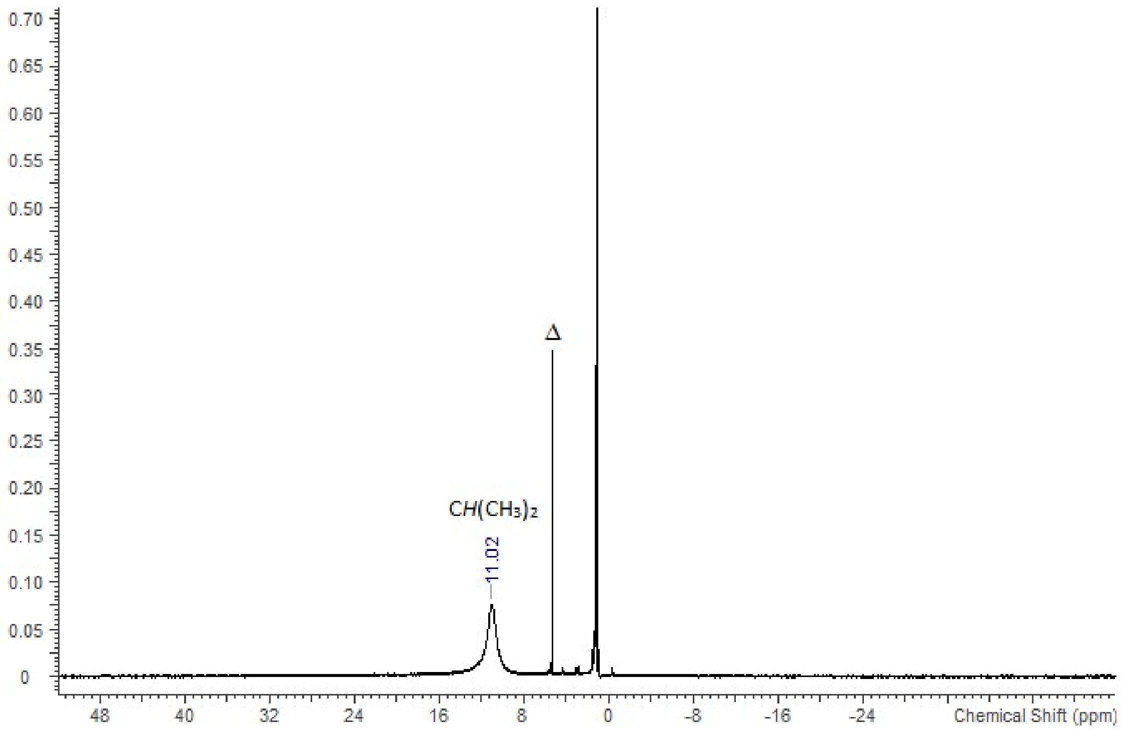

Figure S10. ^{1}H NMR spectrum (swept from +200 to –200 ppm) of **2-Yb** in CD_2Cl_2, zoomed in the region +50 to –50 ppm. Δ denotes solvent residual. Signal at 1.01 ppm assigned to trace $HN(Si^{i}Pr_3)_2$ impurity.

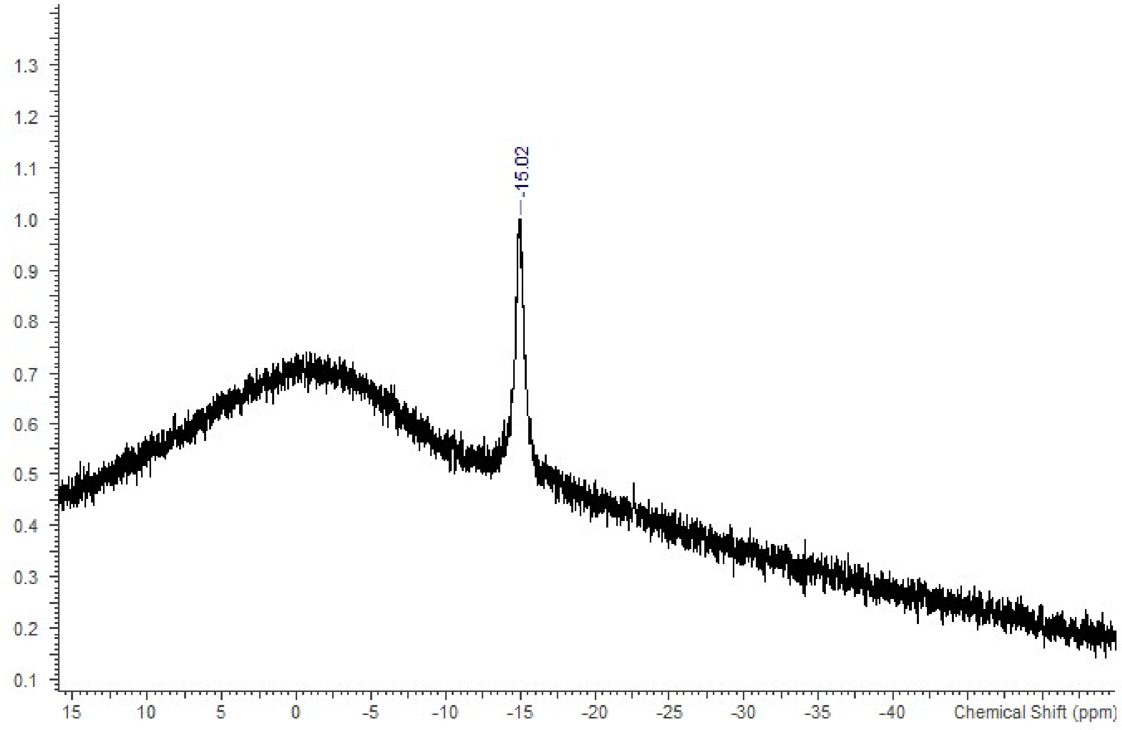

Figure S11. ^{11}B NMR spectrum of **2-Yb** in CD_2Cl_2.

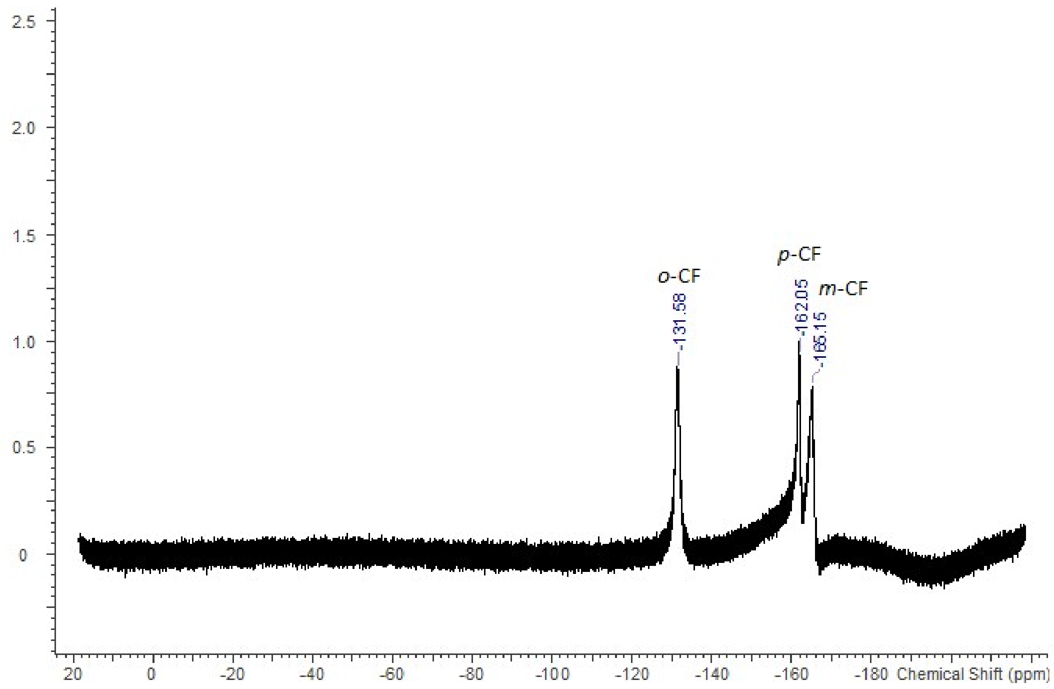

Figure S12. ^{19}F NMR spectrum of **2-Yb** in CD_2Cl_2.

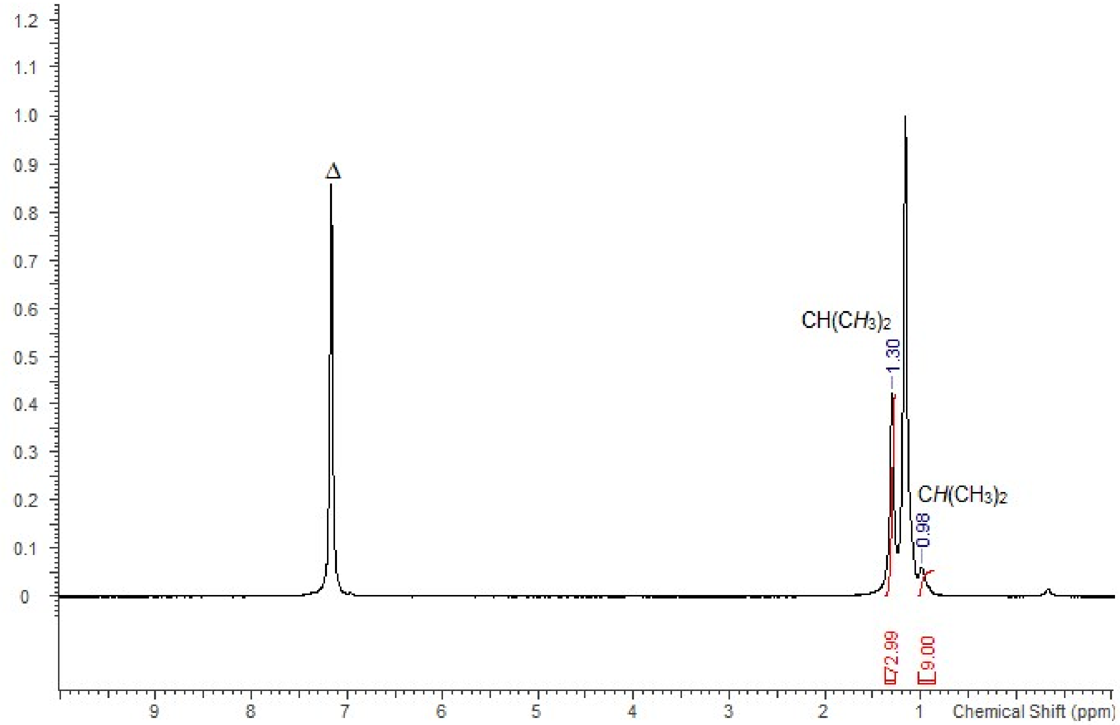

Figure S13. ^{1}H NMR spectrum (swept from +200 to –200 ppm) of **3-Yb** in CD_2Cl_2, zoomed in the region +10 to –2 ppm. Δ denotes solvent residual. Signals observed are assigned to diamagnetic impurities; a similar 1H NMR spectrum was observed for **3-Tm**.

4. ATR-IR spectroscopy

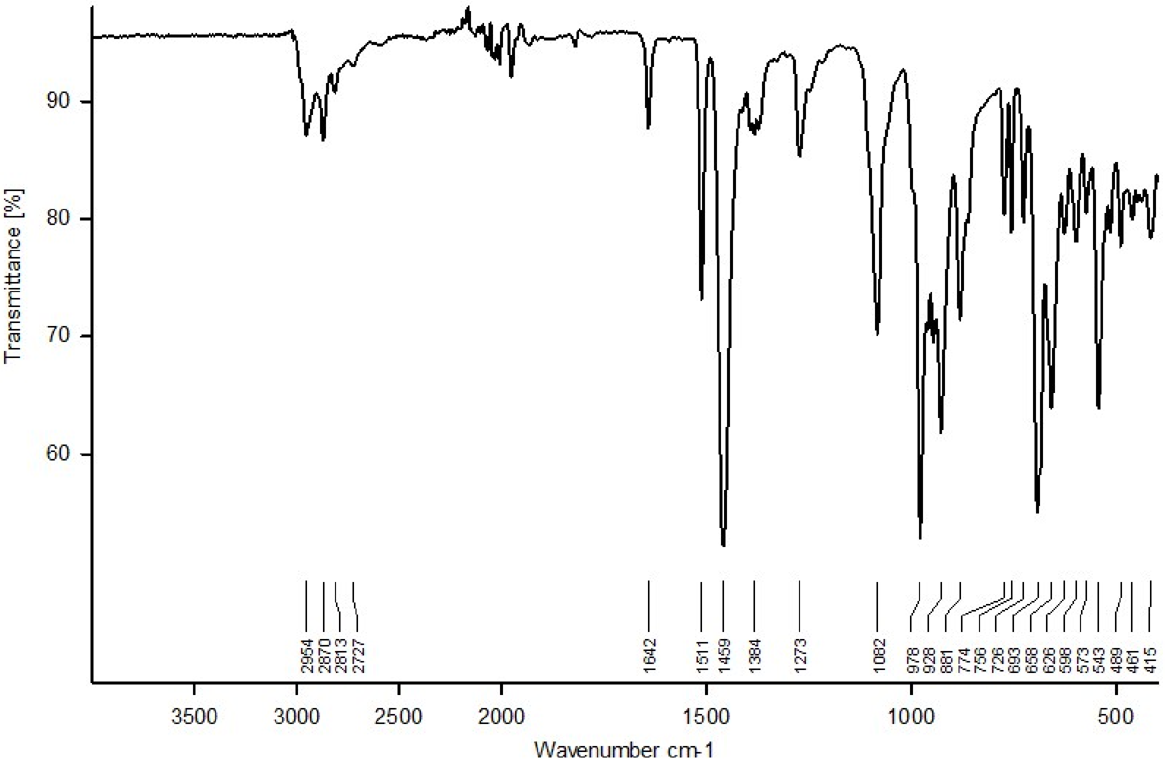

Figure S14. ATR-IR spectrum of **2-Sm** as a microcrystalline powder.

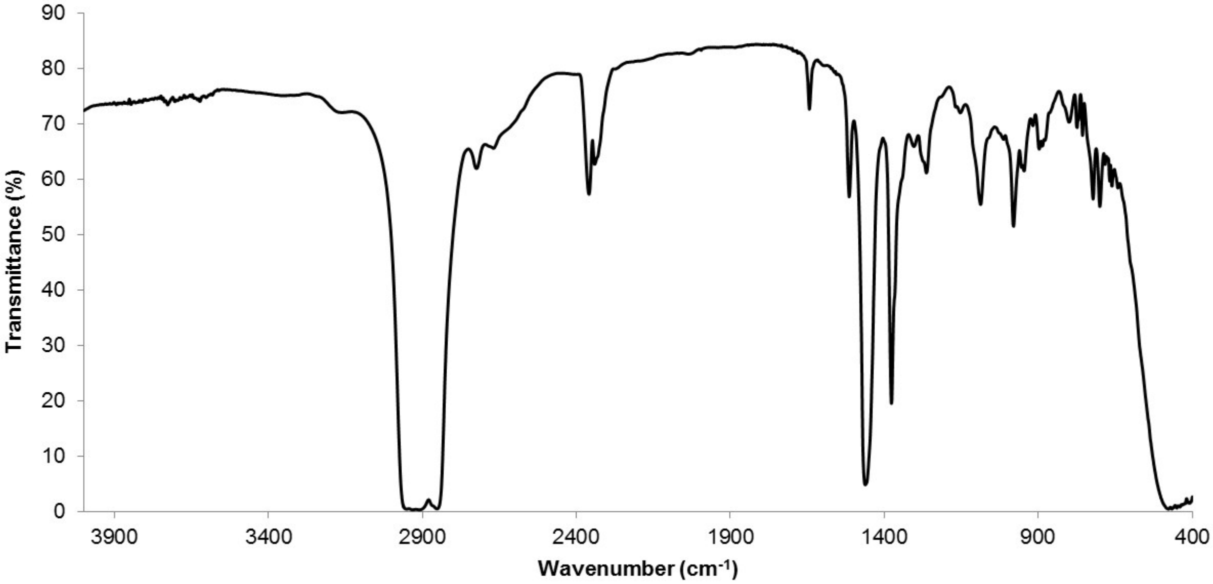

Figure S15. FTIR spectrum of **2-Tm** as a Nujol mull on KBr discs.

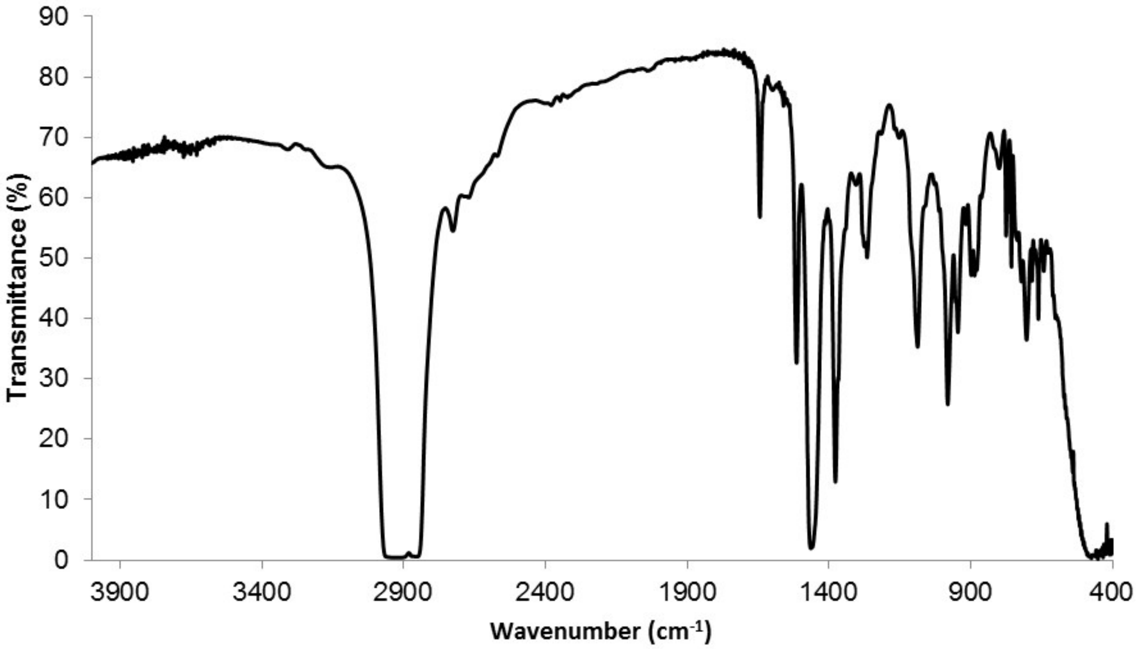

Figure S16. FTIR spectrum of **2-Yb** as a Nujol mull on KBr discs.

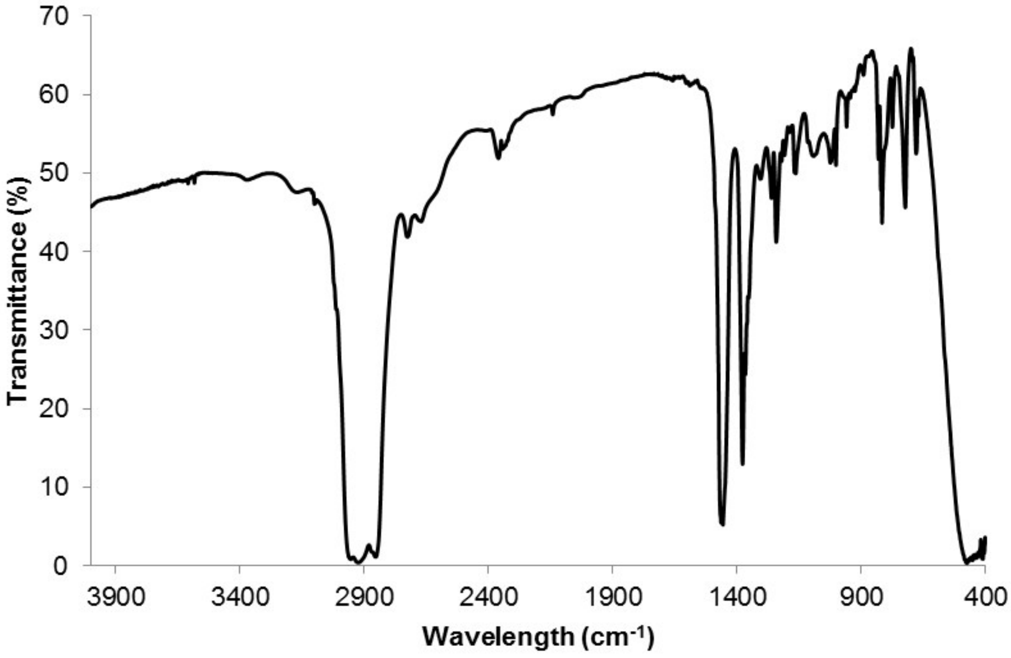

Figure S17. FTIR spectrum of **3-Tm** as a Nujol mull on KBr discs.

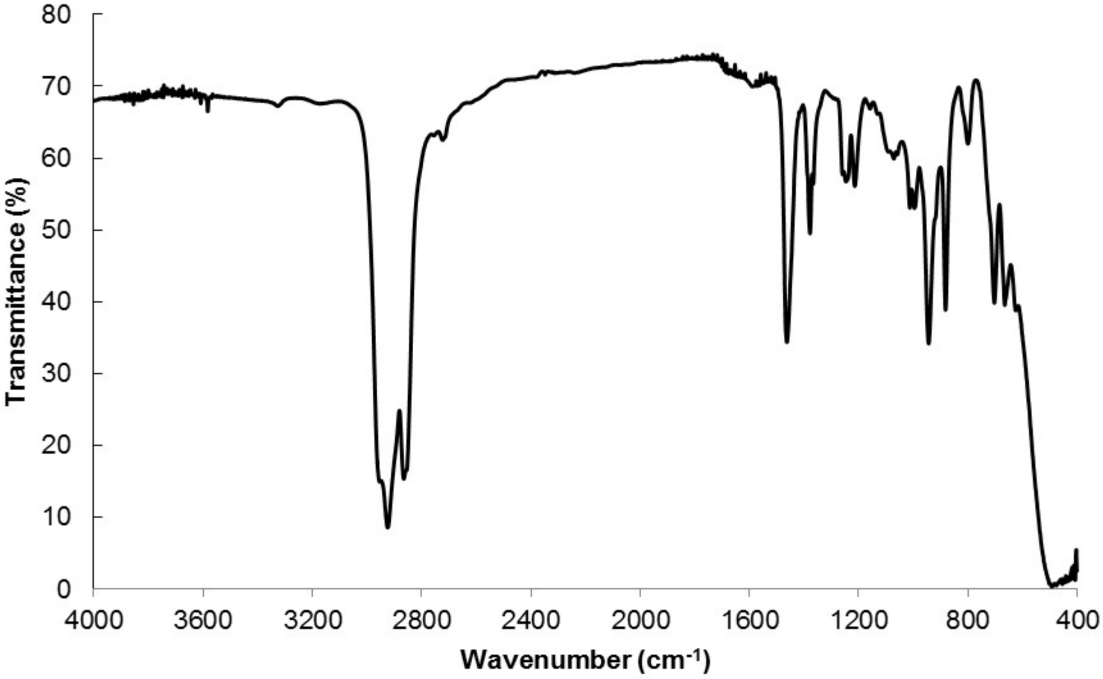

Figure S18. FTIR spectrum of **3-Yb** as a Nujol mull on KBr discs.

5. UV-vis-NIR spectroscopy

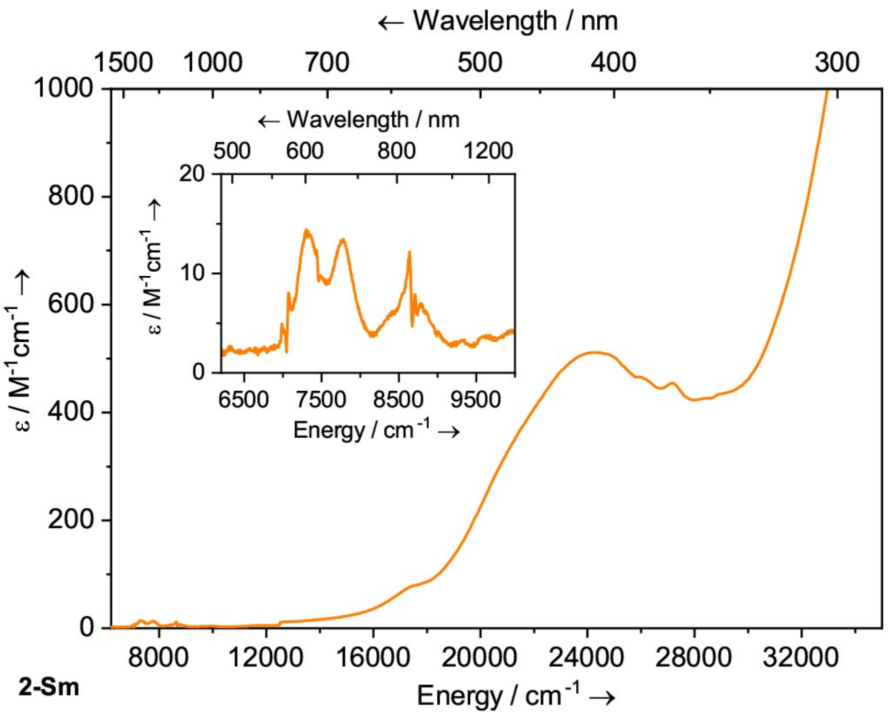

Figure S19. Room temperature UV-vis-NIR spectrum of **2-Sm** (1 mM in DCM) from 6,000–35,000 cm^{-1}.

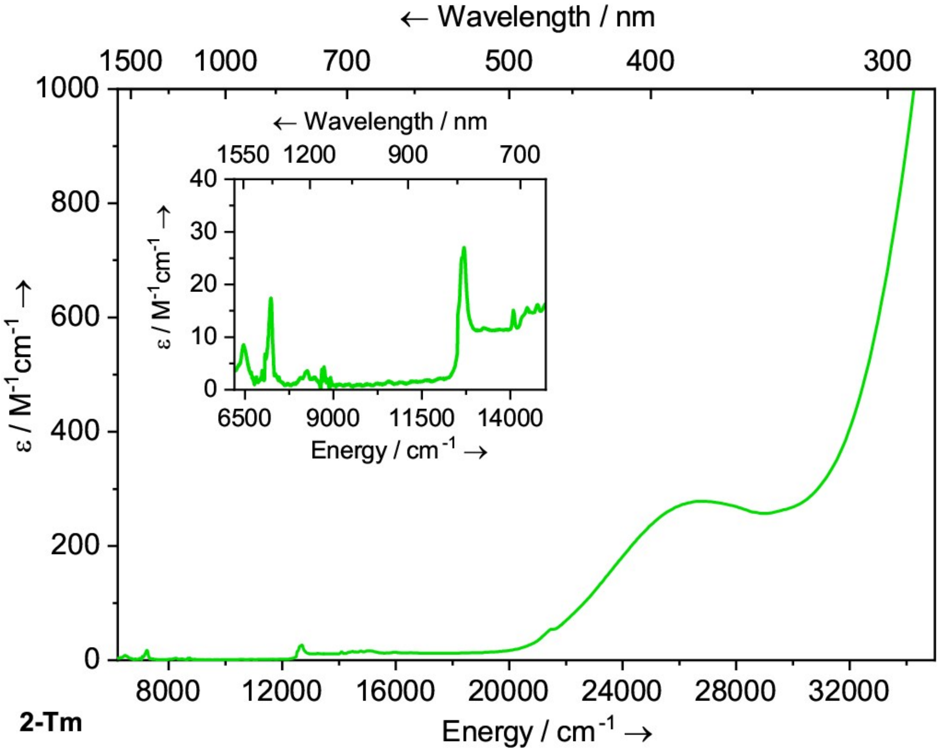

Figure S20. Room temperature UV-vis-NIR spectrum of **2-Tm** (1 mM in DCM) from 6,200–35,000 cm^{-1}. An empirical absorption correction of ε + 2.7 mol^{-1} dm^{3} cm^{-1} has been applied.

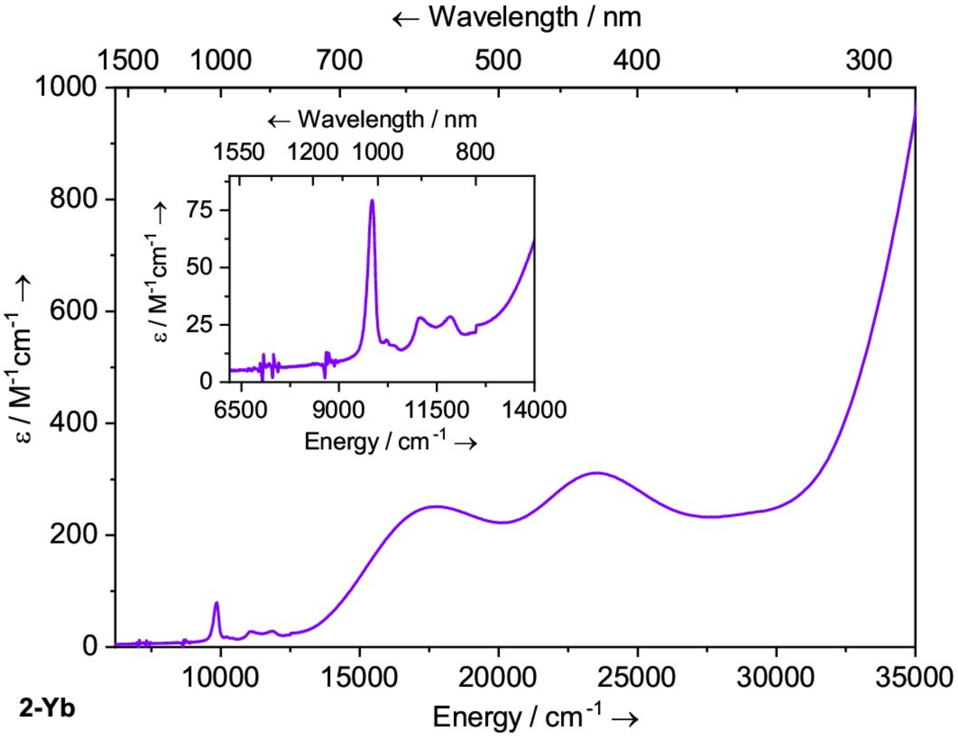

Figure S21. Room temperature UV-vis-NIR spectrum of **2-Yb** (1 mM in DCM) from 6,200–35,000 cm^{-1}. An empirical absorption correction of ε + 2.0 mol^{-1} dm^{3} cm^{-1} has been applied.

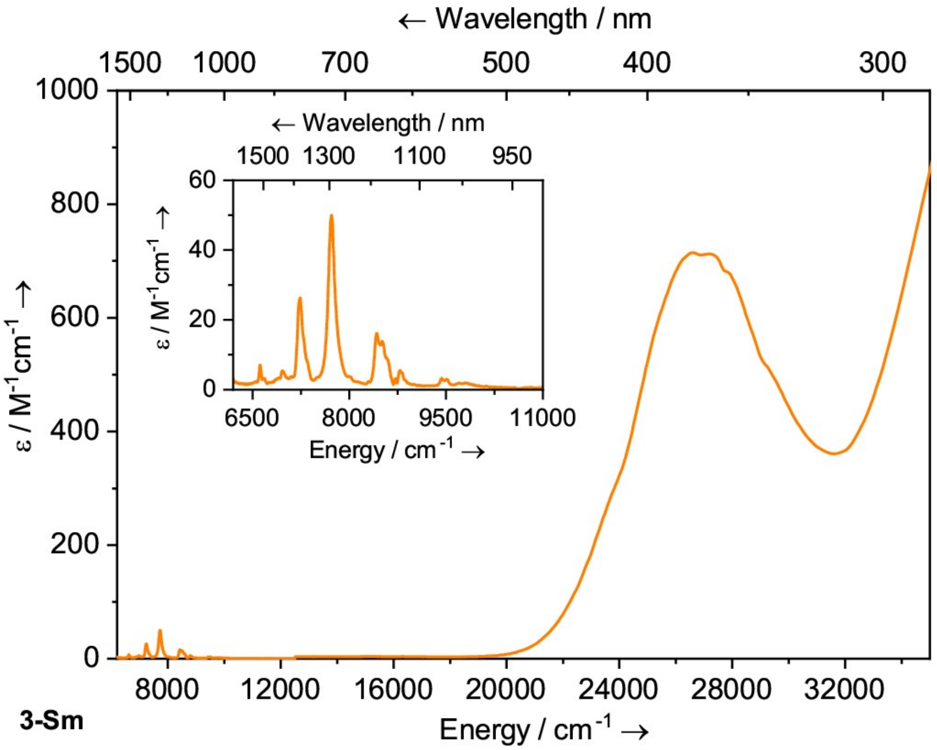

Figure S22. Room temperature UV-vis-NIR spectrum of **3-Tm** (1 mM in THF) from 6,100–35,000 cm^{-1}.

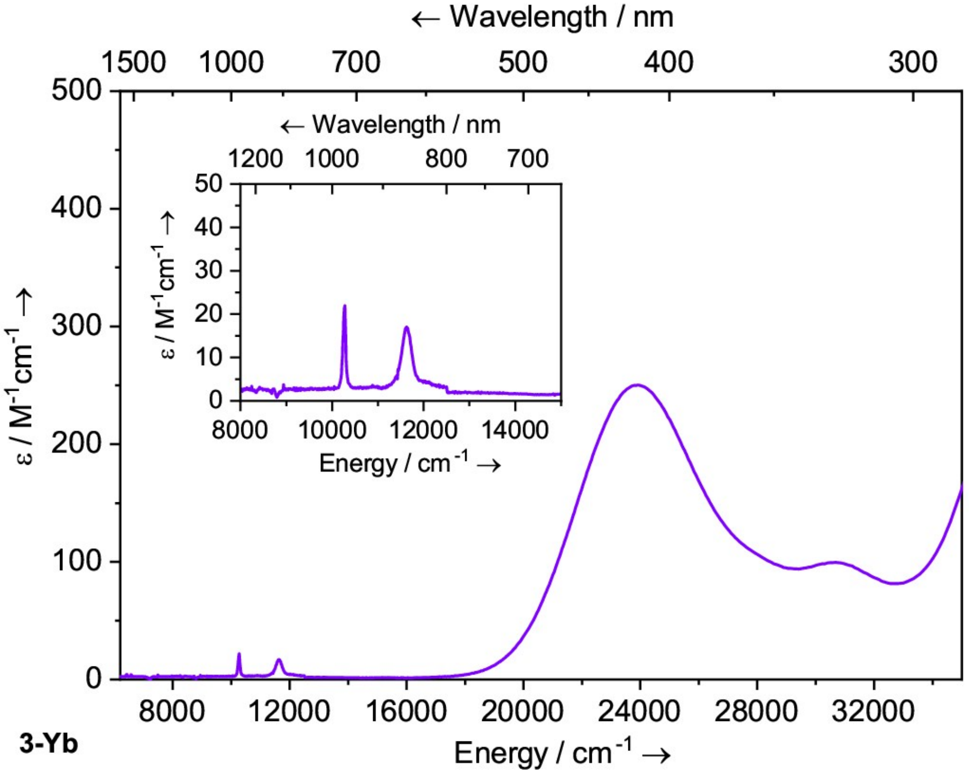

Figure S23. Room temperature UV-vis-NIR spectrum of **3-Yb** (1 mM in THF) from 6,100–35,000 cm^{-1}.

6. Magnetism, EPR spectroscopy and CASSCF-SO electronic structure

Magnetic measurements were performed using a Quantum Design MPMS-XL7 superconducting quantum interference device (SQUID) magnetometer. Crystalline samples with mass ranging between 15 and 40 mg were crushed with a mortar and pestle under an inert atmosphere, and then loaded into a borosilicate glass NMR tube along with *ca.* 5 - 20 mg powdered eicosane, which was then evacuated and flame-sealed to a length of *ca.* 5 cm. The eicosane was melted by heating the tube gently with a low-power heat gun in order to immobilize the crystallites. The NMR tube was then mounted in the centre of a drinking straw using friction by wrapping it with Kapton tape, and the straw was then fixed to the end of the sample rod. The measurements were corrected for the diamagnetism of the straw, borosilicate tube and eicosane using calibrated blanks, and the intrinsic diamagnetism of the sample using Pascal's constants.[7]

CASSCF-SO calculations were performed with the program MOLCAS 8.0[8] using the CASSCF/RASSI/SINGLE_ANISO approach,[9] employing structures as determined by XRD with no optimization and no counterion or solvent molecules. For all calculations the Sm, Tm and Yb atoms were treated with the ANO-RCC-VTZP basis, the N donors atoms with the ANO-RCC-VDZP basis, while all other atoms were treated with the ANO-RCC-VDZ basis.[10] In order to save disk space the two electron integrals were decomposed using the Cholesky decomposition with a high threshold of 10^{-8}. The electronic configuration of Sm^{III} ($4f^6$), Tm^{III}, and Yb^{III} ($4f^9$) was modelled with a complete active space of 5, 12, and 13 electrons respectively in the 7 *f* orbitals. The spin multiplets that were included in the orbital optimisation of the spin-only wave functions were 21 sextets, 224 quartets, and 490 doublets for Sm^{III}; 21 triplets and 28 singlets for Tm^{III}; 7 doublets for Yb^{III}. Due to calculation power limitation a selected number of states was allowed to be mixed by spin-orbit coupling, specifically 21 sextets, 128 quartets, and 130 doublets for Sm^{III}, 21 triplets and 28 singlets for Tm^{III}, and 7 doublets for Yb^{III}. The SINGLE_ANISO module was used to compute the magnetic properties of the complexes and to obtain the CFPs by projecting the lowest lying CASSCF-SO wave functions onto a $(2J + 1)$-dimensional pseudo-spin basis.[11] These CFPs were used with the software PHI in order to calculate the in-field wave function composition of **2-Tm** and **3-Tm**.[12]

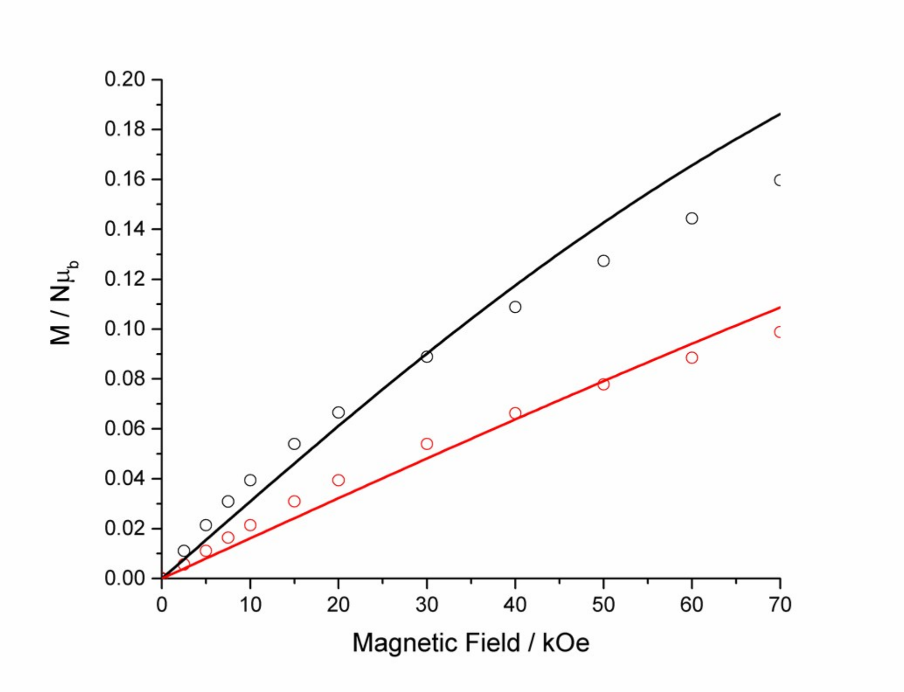

Figure S24. Experimental (open circles) and CASSCF-SO (solid lines) magnetization isotherms at 2 (black) and 4 K (red) for **2-Sm**.

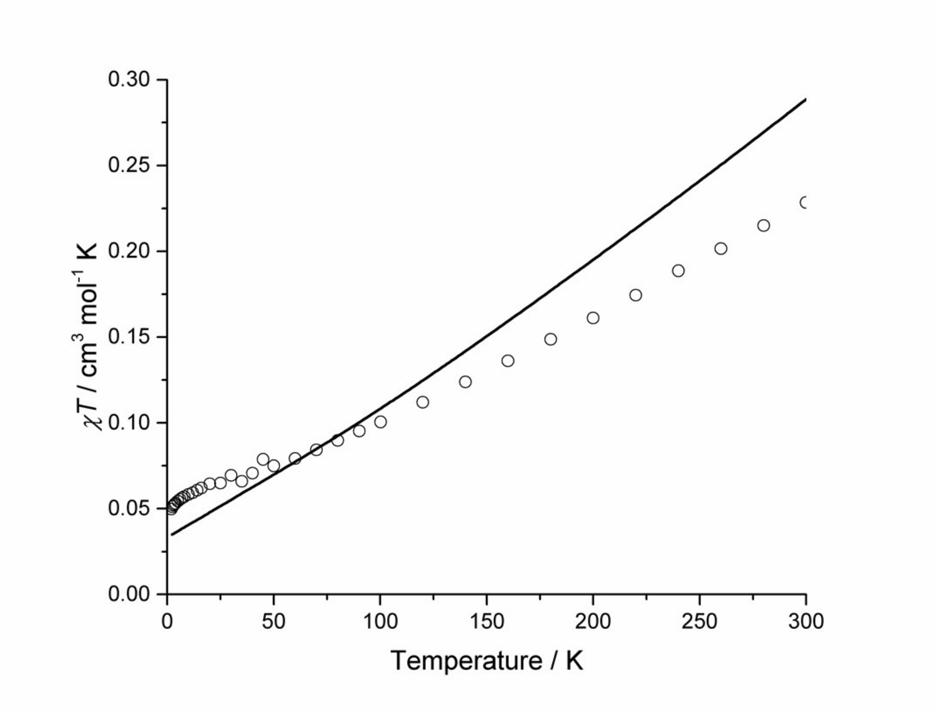

Figure S25. Experimental (open circles) 2-300 K χT temperature dependence in 5000 Oe (2-20 K) and 10000 Oe (20-300 K) applied field and CASSCF-SO (solid line) theoretical curve for **2-Sm**.

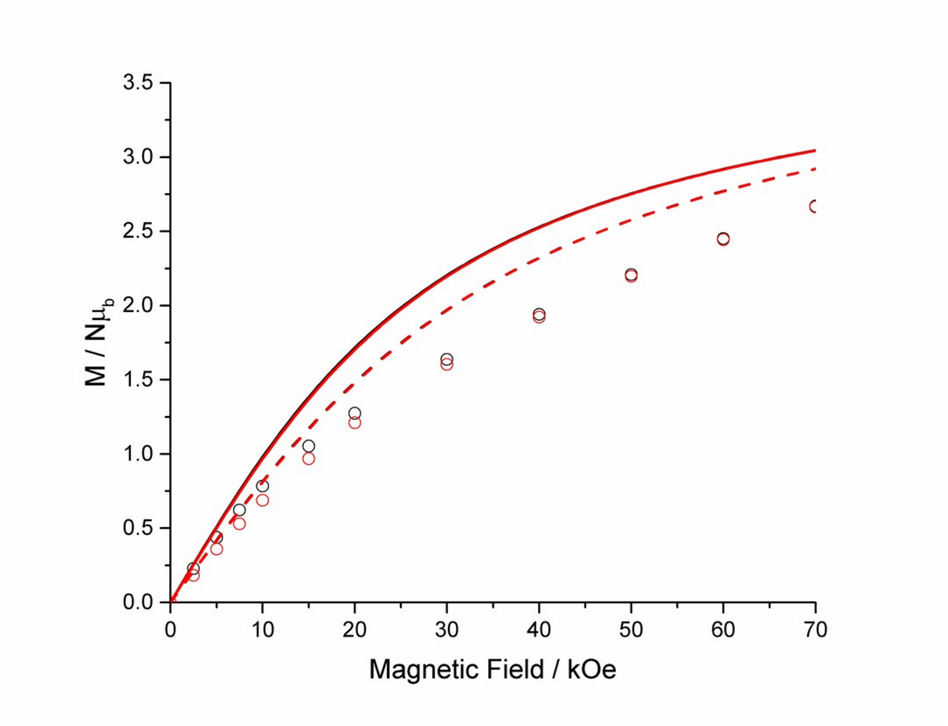

Figure S26. Experimental (open circles) and CASSCF-SO magnetization isotherms at 2 (black) and 4 K (red) for **2-Tm**. CASSCF-SO calculations have been performed on the two crystallographically distinct molecular moieties in the unit cell: solid lines for results for the molecule containing Tm1, dashed lines for the molecule containing Tm2.

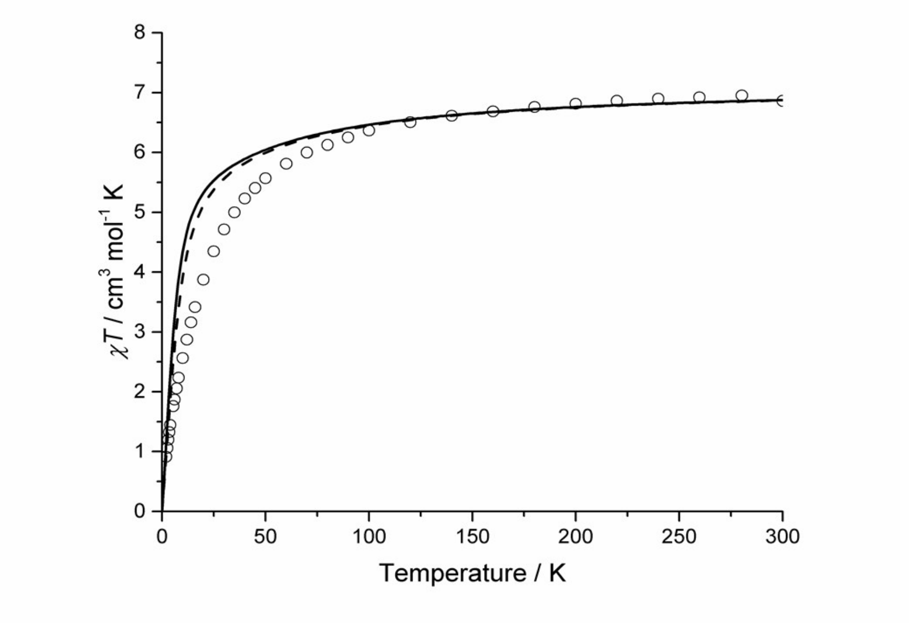

Figure S27. Experimental (open circles) 2-300 K χT temperature dependence in 1000 Oe applied field and CASSCF-SO theoretical curves for **2-Tm**. CASSCF-SO calculations have been performed on the two crystallographically distinct molecular moieties in the unit cell: solid lines for results for the molecule containing Tm1, dashed lines for the molecule containing Tm2.

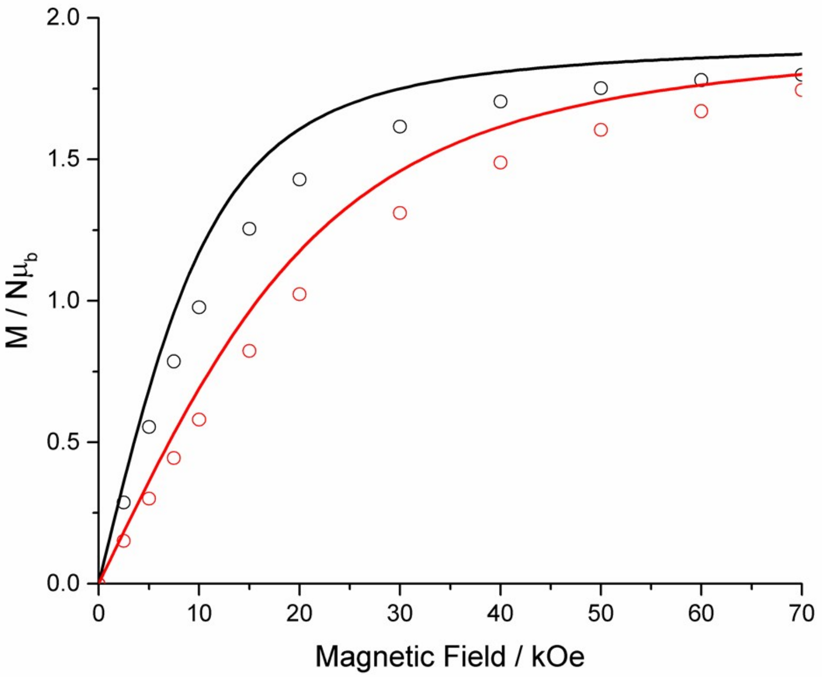

Figure S28. Experimental (open circles) and CASSCF-SO (solid lines) magnetization isotherms at 2 (black) and 4 K (red) for **2-Yb**.

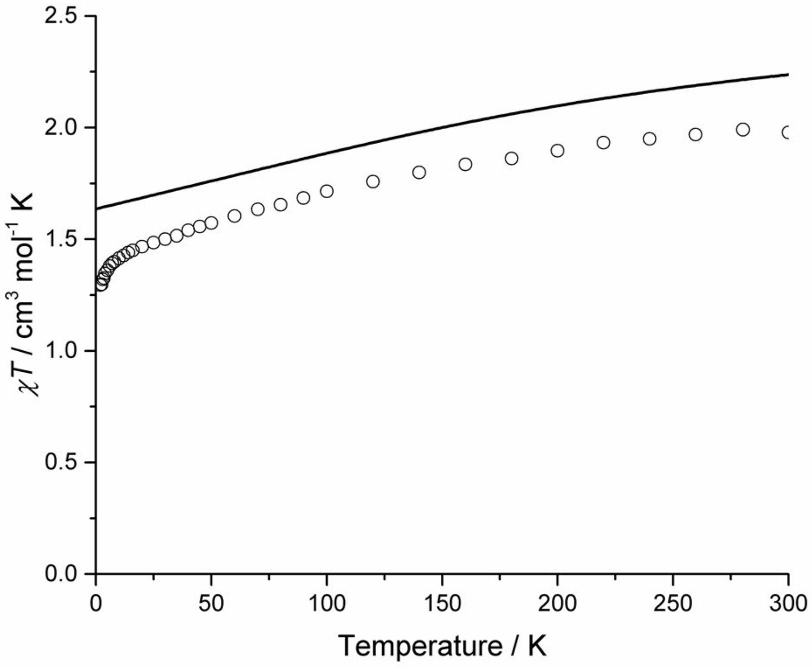

Figure S29. Experimental (open circles) 2-300 K χT temperature dependence in 1000 Oe applied field and CASSCF-SO (solid line) theoretical curve for **2-Yb**.

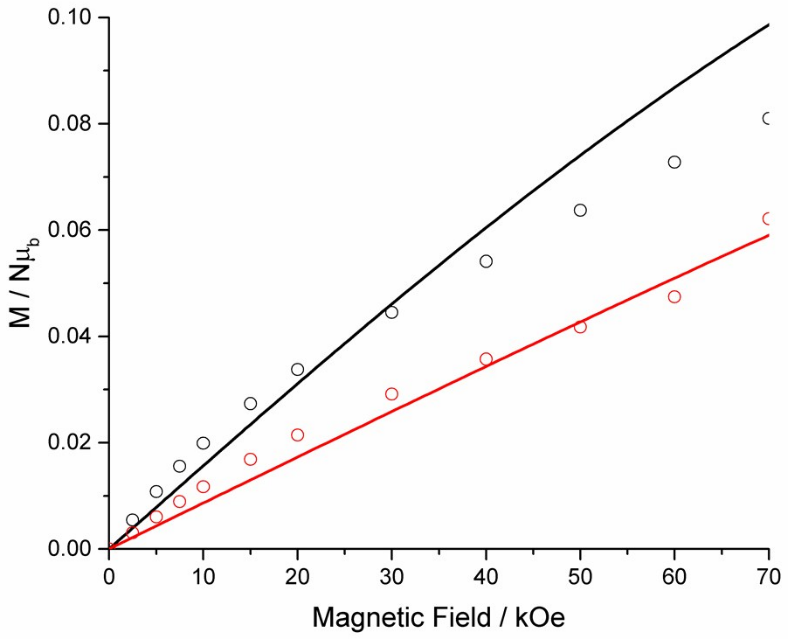

Figure S30. Experimental (open circles) and CASSCF-SO (solid lines) magnetization isotherms at 2 (black) and 4 K (red) for **3-Sm**.

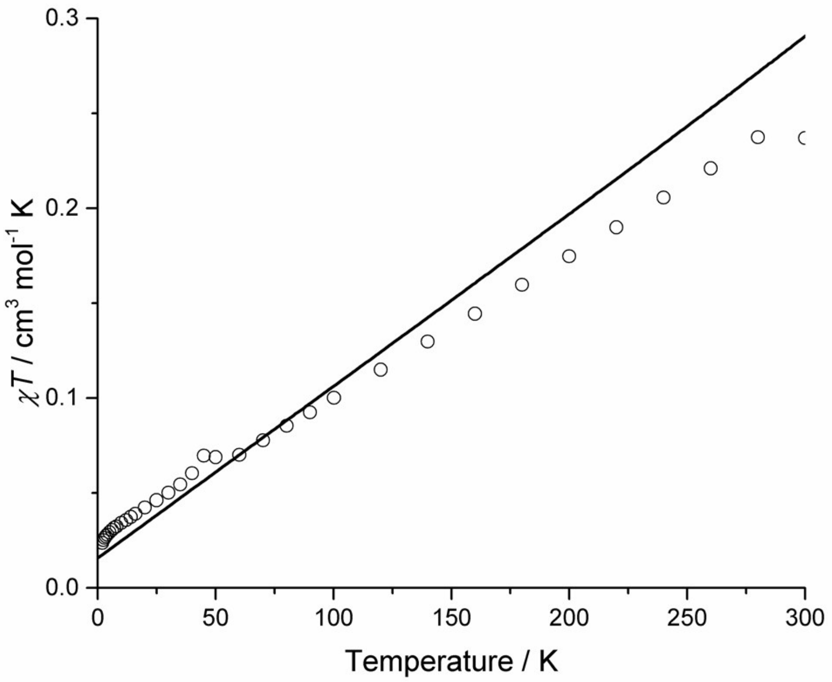

Figure S31. Experimental (open circles) 2-300 K χT temperature dependence 10000 Oe applied field and CASSCF-SO (solid line) theoretical curve for **3-Sm**.

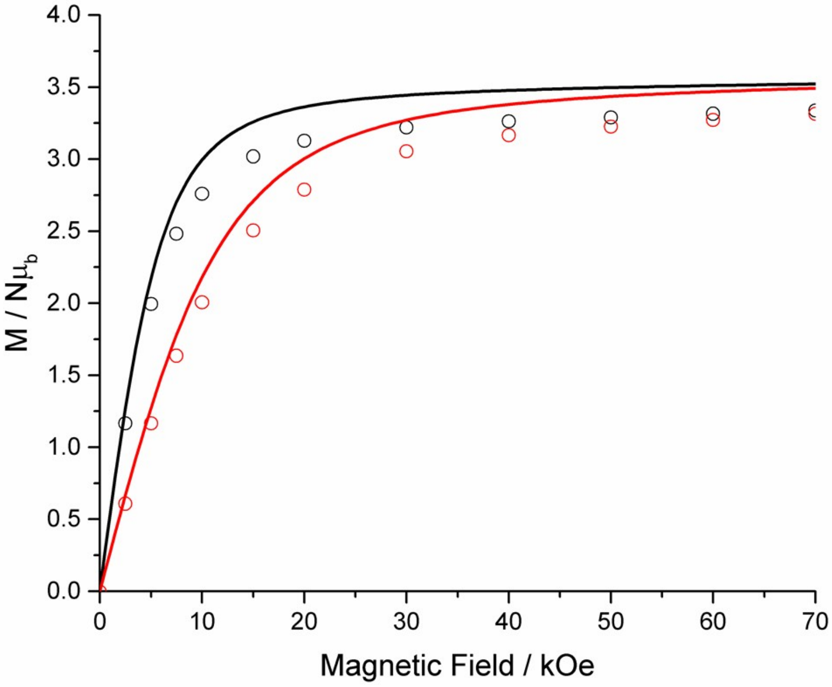

Figure S32. Experimental (open circles) and CASSCF-SO (solid lines) magnetization isotherms at 2 (black) and 4 K (red) for **3-Tm**.

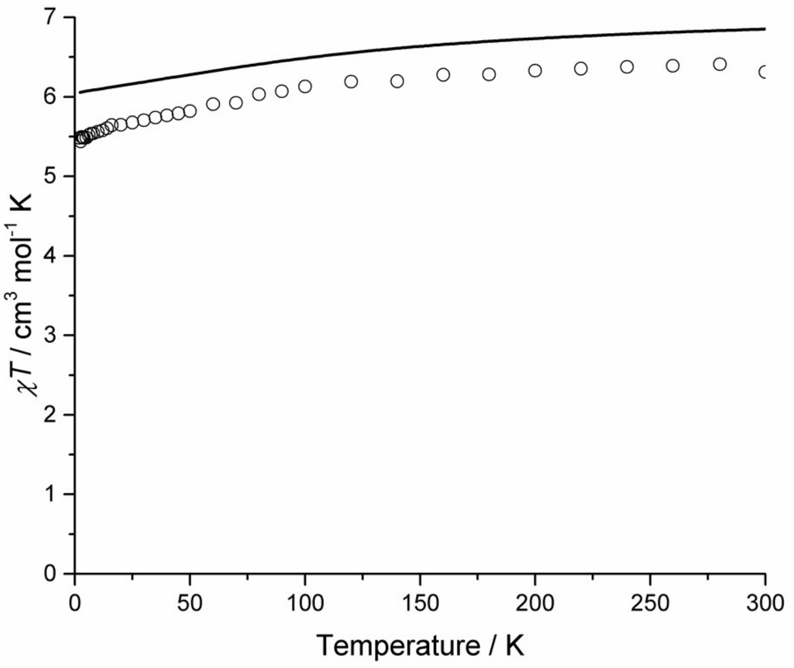

Figure S33. Experimental (open circles) 2-300 K χT temperature dependence in 1000 Oe applied field and CASSCF-SO (solid line) theoretical curve for **3-Tm**.

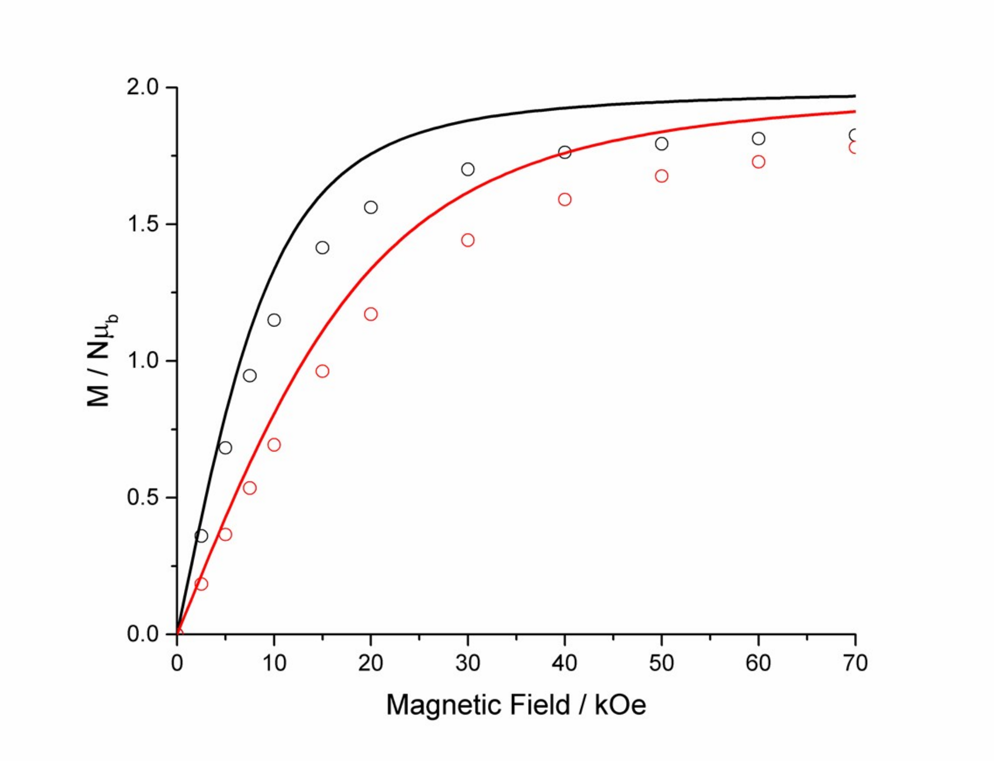

Figure S34. Experimental (open circles) and CASSCF-SO (solid lines) magnetisation isotherms at 2 (black) and 4 K (red) for **3-Yb**.

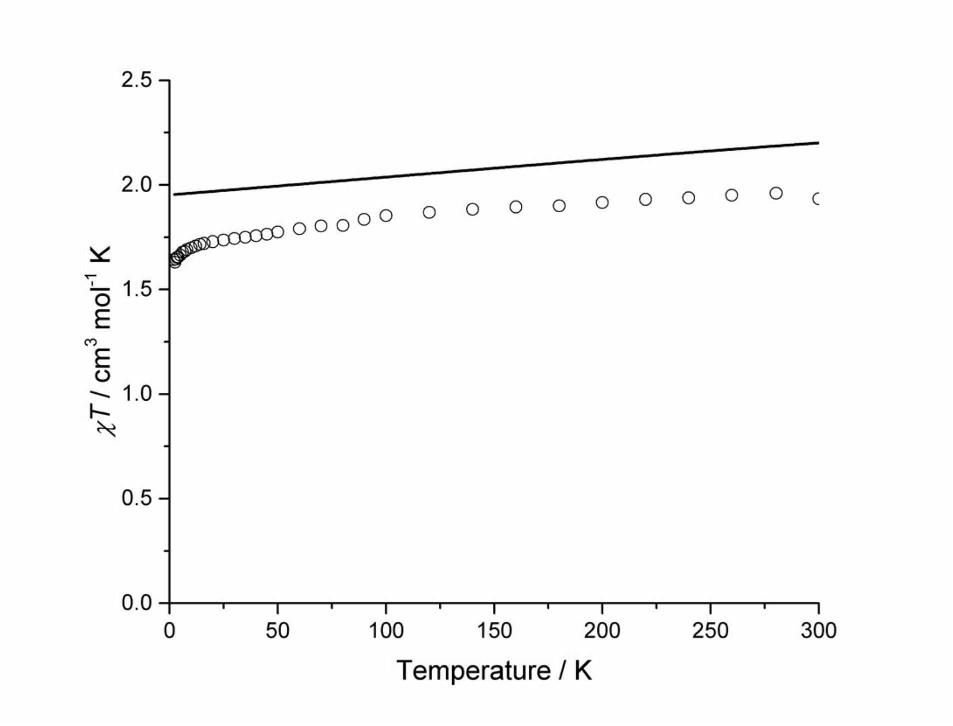

Figure S35. Experimental (open circles) 2-300 K χT temperature dependence in 1000 Oe applied field and CASSCF-SO (solid line) theoretical curve for **3-Yb**.

Table S3. CASSCF-SO energies (cm^{-1}) of the states corresponding to the Russell-Saunders ground state multiplet for **2-Ln** and **3-Ln.**

**2-Yb**	**3-Yb**	**2-Sm**	**3-Sm**	**2-Tm(Tm1)**	**2-Tm(Tm2)**	**3-Tm**
0.000	0.000	0.000	0.000	0.000	0.000	0.000
0.000	0.000	0.000	0.000	13.28356478	16.08515818	0.133259
490.3054	992.5129	306.8354	522.6088	121.8005117	107.7836067	318.0041
490.3054	992.5129	306.8354	522.6088	198.0878091	197.6502197	319.0755
1038.453	1488.225	947.8721	1051.453	244.3786101	223.6609887	674.5274
1038.453	1488.225	947.8721	1051.453	433.5337136	430.5619677	688.55
1819.963	1918.833			458.866744	453.0834139	912.7948
1819.963	1918.833			774.2921221	785.4236917	1017.674
				783.8165126	792.4769861	1084.05
				1215.215941	1249.590776	1354.284
				1216.698621	1250.545118	1358.753
				1769.559927	1832.639687	1766.986
				1769.643357	1832.694104	1767.05

Table S4. CASSCF-SO calculated EPR data for **2-Sm** and **3-Sm**.

Complex	N–Ln–N/°	Calculated *g*-values		
		g_1	g_2	g_3
**2-Sm**	131.018	0.7778	0.5920	0.3269
**3-Sm**	128.263	0.7053	0.0097	0.0770

Table S5. CASSCF-SO percentage wave function composition for **3-Tm** when the quantization axis is oriented perpendicularly to the N-N-Cl plane in a zero applied field.

M_J	WF 1	WF 2	WF 3	WF 4	WF 5	WF 6	WF 7	WF 8	WF 9	WF 10	WF 11	WF 12	WF 13
-6	49.6	49.6	0.0	0.0	0.3	0.3	0.1	0.1	0.0	0.0	0.0	0.0	0.0
-5	0.0	0.0	48.2	48.3	0.1	0.2	1.2	1.3	0.2	0.2	0.3	0.0	0.1
-4	0.3	0.3	0.1	0.1	42.1	45.0	0.2	0.6	6.0	3.5	0.9	0.8	0.0
-3	0.1	0.1	1.6	1.6	0.2	0.2	28.1	42.3	0.6	2.7	17.4	0.0	5.3
-2	0.0	0.0	0.0	0.0	6.3	4.4	0.1	0.2	23.2	41.5	4.0	20.2	0.0
-1	0.0	0.0	0.0	0.0	0.0	0.0	20.4	5.4	0.0	2.1	27.4	0.0	44.5
0	0.0	0.0	0.1	0.0	1.9	0.0	0.0	0.2	39.9	0.0	0.0	57.9	0.0
1	0.0	0.0	0.0	0.0	0.0	0.0	20.4	5.4	0.0	2.1	27.4	0.0	44.5
2	0.0	0.0	0.0	0.0	6.3	4.4	0.1	0.2	23.2	41.5	4.0	20.2	0.0
3	0.1	0.1	1.6	1.6	0.2	0.2	28.1	42.3	0.6	2.7	17.4	0.0	5.3
4	0.3	0.3	0.1	0.1	42.1	45.0	0.2	0.6	6.0	3.5	0.9	0.8	0.0
5	0.0	0.0	48.2	48.3	0.1	0.2	1.2	1.3	0.2	0.2	0.3	0.0	0.1
6	49.6	49.6	0.0	0.0	0.3	0.3	0.1	0.1	0.0	0.0	0.0	0.0	0.0

Table S6. CASSCF-SO percentage wave function composition for **3-Tm** when the quantization axis is oriented perpendicularly to the N-N-Cl plane in 0.1 T applied field.

M_J	WF 1	WF 2	WF 3	WF 4	WF 5	WF 6	WF 7	WF 8	WF 9	WF 10	WF 11	WF 12	WF 13
-6	98.4	0.9	0.0	0.0	0.3	0.3	0.1	0.1	0.0	0.0	0.0	0.0	0.0
-5	0.0	0.0	69.9	26.6	0.1	0.2	1.2	1.3	0.2	0.2	0.3	0.0	0.1
-4	0.6	0.0	0.2	0.1	43.4	43.7	0.2	0.6	6.0	3.5	0.9	0.8	0.0
-3	0.1	0.0	2.3	0.8	0.2	0.2	28.1	42.2	0.6	2.7	17.4	0.1	5.3
-2	0.0	0.0	0.0	0.0	6.5	4.2	0.1	0.2	23.2	41.5	4.0	20.2	0.0
-1	0.0	0.0	0.1	0.0	0.0	0.0	20.4	5.4	0.0	2.1	27.5	0.1	44.4
0	0.0	0.0	0.1	0.0	1.9	0.0	0.0	0.2	39.9	0.0	0.0	57.8	0.1
1	0.0	0.0	0.0	0.0	0.0	0.0	20.4	5.4	0.0	2.1	27.4	0.0	44.6
2	0.0	0.0	0.0	0.0	6.2	4.5	0.1	0.2	23.2	41.6	4.0	20.1	0.1
3	0.0	0.1	0.9	2.3	0.2	0.2	28.0	42.3	0.6	2.7	17.4	0.0	5.3
4	0.0	0.6	0.1	0.2	40.8	46.3	0.2	0.6	6.0	3.5	0.9	0.8	0.0
5	0.0	0.0	26.5	70.0	0.1	0.2	1.2	1.3	0.2	0.2	0.3	0.0	0.1
6	0.9	98.4	0.0	0.0	0.3	0.3	0.1	0.1	0.0	0.0	0.0	0.0	0.0

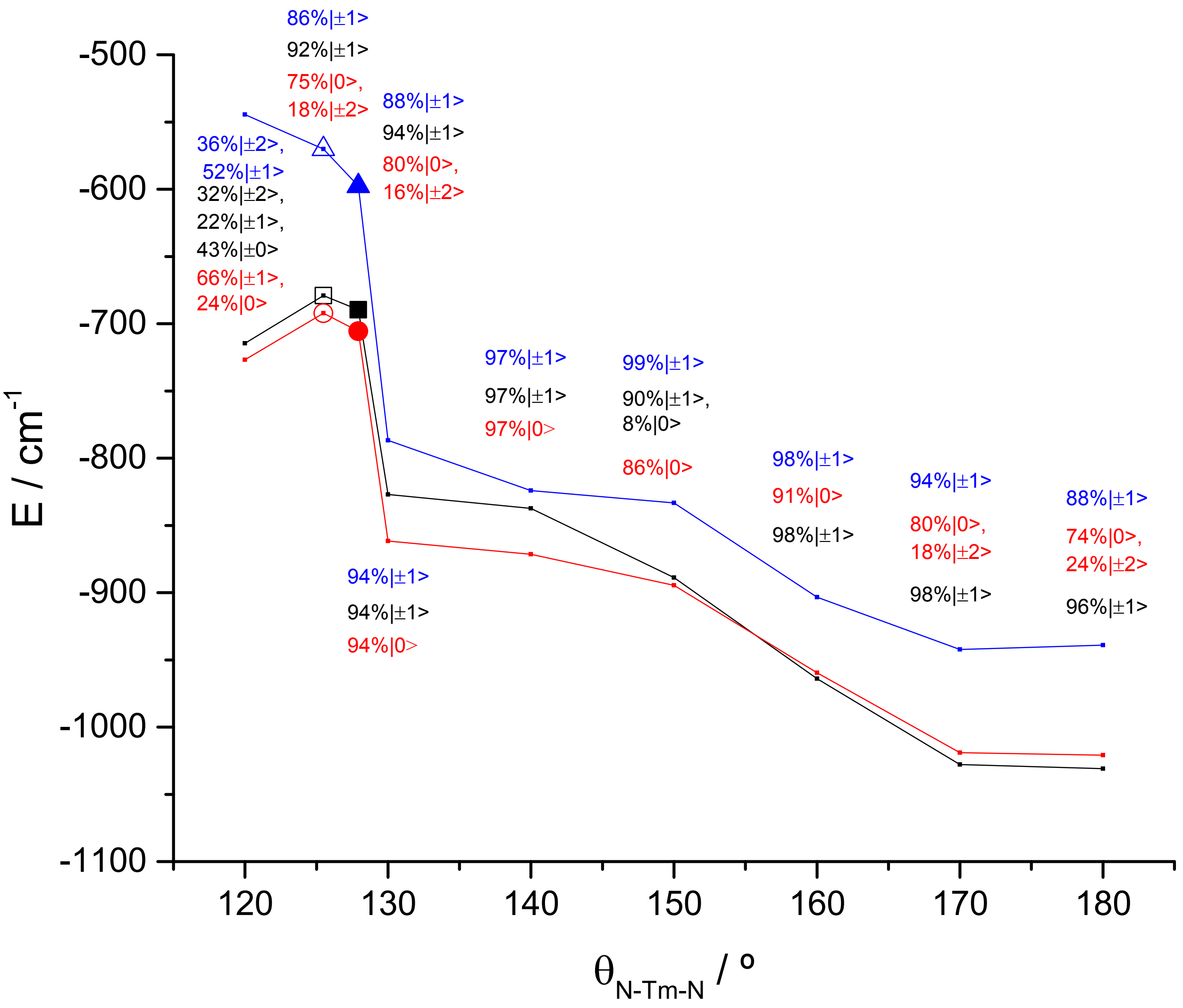

Figure S36. Calculated wave function composition in an applied 0.1 T field for the lowest three states of model structures based on **2-Tm** as a function of the N–Tm–N angle (lines) quantized along the direction that bisects the N–Tm–N angle. CASSCF-SO values based on the two crystallographically distinct molecules in XRD experimental structure of **2-Tm** containing Tm1 (open symbols) and Tm2 (solid symbols). Colour code for the states: singlet state (red), *pseudo*-doublet (black and blue).

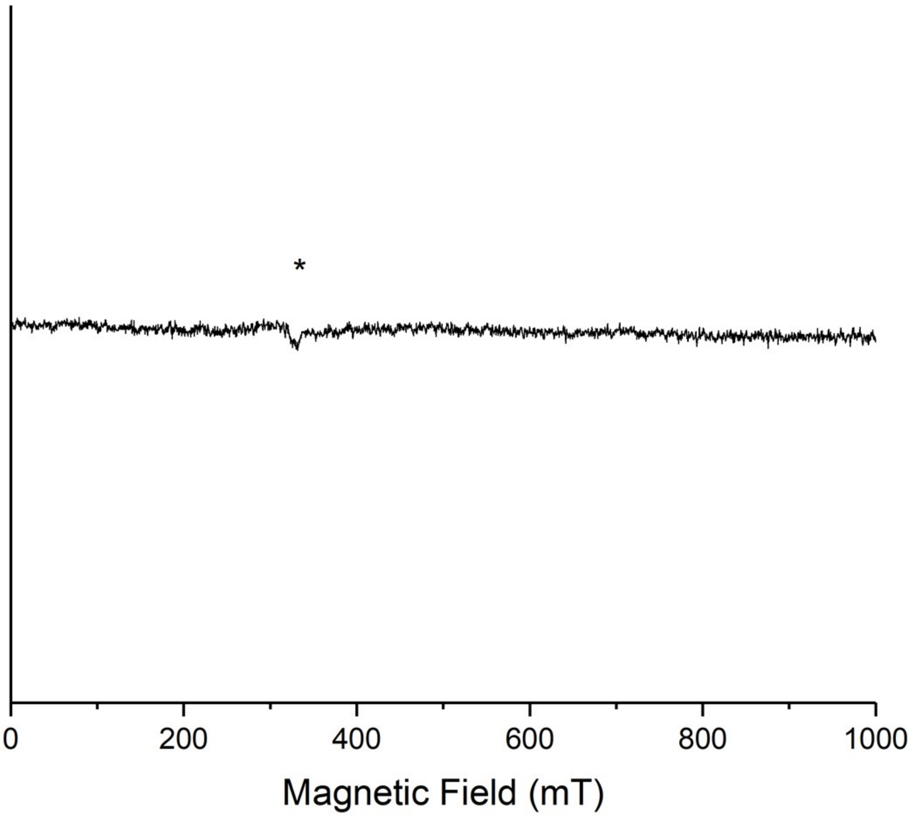

Figure S37. X-band EPR spectrum of solid **2-Tm** collected at 5 K. The star indicates an impurity in the cavity at *g* ~ 2.

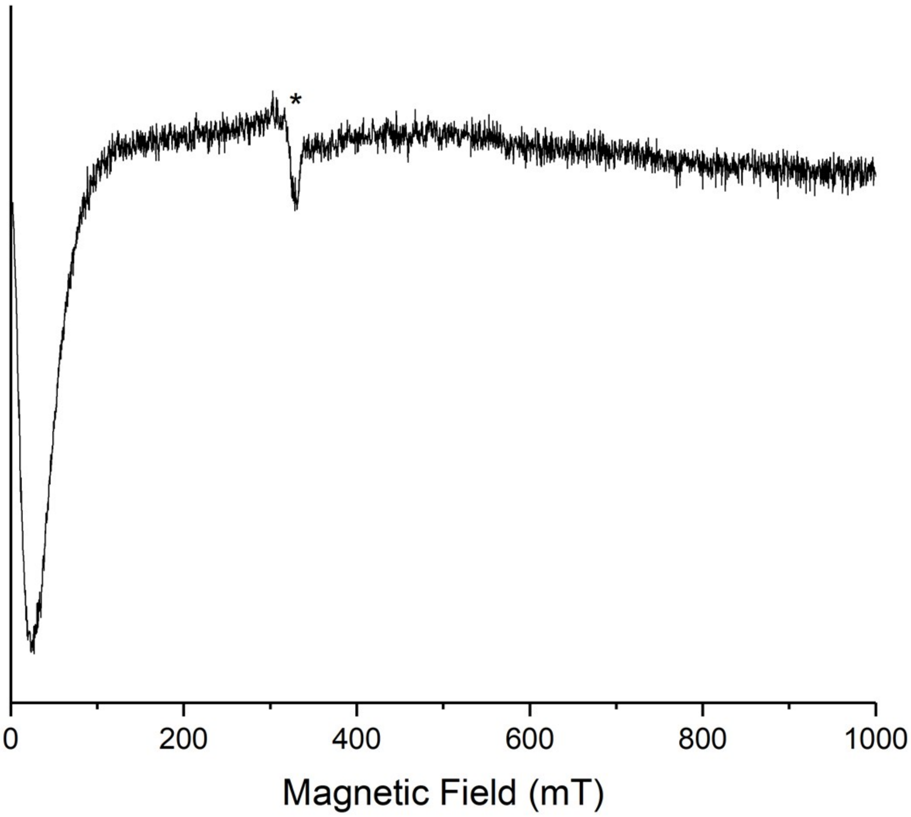

Figure S38. X-band EPR spectrum of solid **3-Tm** collected at 5 K. The star indicates an impurity in the cavity at *g* ~ 2.

Table S7. CASSCF-SO percentage wave function composition angular dependence for **2-Tm** when the quantization axis is oriented along the N-N direction in a 0.1 T applied field.

N-Tm-N angle / º	M_J	WF 1	WF 2	WF 3	WF 4	WF 5	WF 6	WF 7	WF 8	WF 9	WF 10	WF 11	WF 12	WF 13
	-6	1.9524956	4.4977437	27.995493	2.977899	40.770556	16.519317	0.6580097	3.8716331	0.0426595	0.5326937	0.1265513	0.000482	0.0544675
	-5	10.478253	2.0744029	3.3722172	25.285929	0.3617337	0.8266481	33.092555	0.0931775	18.554099	1.1982235	4.0305661	0.5966195	0.0355745
	-4	1.55705	12.862049	10.998448	2.3572845	0.3950123	18.313987	0.1910861	32.390218	0.0918842	13.184743	4.3197447	0.1384432	3.2000497
	-3	16.517705	1.3511607	2.9201416	13.858077	0.394774	0.0157327	0.0623499	0.0512022	22.016935	8.0918226	23.874818	10.340397	0.5048845
	-2	0.6554247	18.459604	2.8825618	2.5194632	3.8597353	14.157678	0.1400203	1.3564479	0.0051181	23.778952	8.0533628	1.3130983	22.818534
	-1	19.072211	0.4639519	1.9149175	1.767948	0.2499907	0.1155767	15.826207	0.0126857	9.2563645	3.2175894	9.5877386	36.684288	1.8305323
**180**	0	0.2633187	20.278445	0.0608876	2.3502006	7.5661791	0.0314823	0.0288111	24.444036	0.0352371	0	0.0045958	2.2589003	42.677902
	1	18.999413	0.5081296	1.9097127	1.8106126	0.254114	0.1178438	15.798746	0.0114567	9.2755779	3.2095861	9.5819192	36.471169	2.0517196
	2	0.5909748	18.467466	2.9552203	2.5422292	3.7891367	14.192406	0.1414689	1.3488146	0.0049091	23.775398	8.0502573	1.115117	23.026602
	3	16.325196	1.4684768	2.9030693	13.943639	0.4026145	0.0159171	0.0598898	0.0495933	22.005644	8.0954178	23.878475	10.296523	0.5555444
	4	1.4496143	12.877935	11.039387	2.3679123	0.4327313	18.292212	0.1899952	32.400387	0.0942799	13.188271	4.3259959	0.163787	3.1774921
	5	10.271574	2.1953951	3.3580038	25.312718	0.3626881	0.8225225	33.144039	0.0924699	18.574458	1.1951152	4.0381609	0.6169249	0.0159307
	6	1.8667702	4.4952405	27.68994	2.9060867	41.160735	16.578677	0.6668225	3.8778783	0.0428326	0.5321841	0.1278152	0.0042521	0.0507665
	-6	41.899205	35.895882	1.4092186	1.4063524	6.8974423	0.0901185	8.7213053	3.0480135	0.0084543	0.0519491	0.5237354	0.0155727	0.0327511
	-5	0.822094	0.8196552	19.719556	32.552296	0.2095107	25.282999	0.197331	0.0229172	15.315883	4.0283984	0.4902929	0.3013962	0.2376716
	-4	8.4597341	6.956942	0.2981582	0.0868893	7.4676098	0.0964834	25.563718	31.744512	0.0620122	1.8421741	14.429045	1.3079896	1.6847318
	-3	0.5612066	0.4102515	16.087587	13.664036	0.1597233	1.5229665	0.000945	0.094165	25.291631	28.43951	3.4499487	6.1607433	4.1572863
	-2	1.4518501	1.7995063	0.2071564	0.0000695	21.8025	0.047027	15.432322	2.1278934	0.0388335	3.4038611	29.722244	10.665463	13.301273
**170**	-1	0.311287	0.1264299	12.791451	1.6760048	0.1060645	22.967077	0.0339806	0.0533519	9.2064173	12.215596	1.3770589	23.174114	15.961166
	0	0.0019686	0.9776877	0.2368204	0.0003528	26.804216	0.0011726	0.0172325	25.810527	0.1280588	0.0403407	0.009623	19.281732	26.690267
	1	0.2946849	0.144416	12.668942	1.8084975	0.1065164	22.939855	0.0365448	0.0579552	9.2204512	12.238602	1.3400978	22.191908	16.95153
	2	1.233314	2.028447	0.2073104	0.0001288	21.749182	0.044451	15.483191	2.1152505	0.0416699	3.412015	29.708318	9.6223623	14.35436
	3	0.4963669	0.4794488	15.678288	14.077676	0.1610859	1.5084347	0.0011609	0.0884726	25.286913	28.421414	3.4762695	5.6856188	4.6388502
	4	7.3817531	8.0514881	0.2957404	0.0919647	7.4214067	0.0921128	25.568202	31.759739	0.0617567	1.8244386	14.456193	1.2448577	1.750347
	5	0.7085448	0.9392067	19.021475	33.187572	0.2106767	25.316535	0.1997136	0.024603	15.329502	4.0289911	0.4935551	0.3251545	0.21447
	6	36.377991	41.370639	1.3782966	1.4481592	6.904066	0.0907677	8.7443533	3.052599	0.0084173	0.0527101	0.5236173	0.0230878	0.0252955
	-6	0.4243737	10.539558	34.201837	0.0203193	35.360862	8.522693	6.5234538	0.1620004	3.5276248	0.4042091	0.2511362	0.0460629	0.015871
	-5	13.405591	0.2532465	0.0300842	31.99347	0.0860331	14.160649	17.237503	16.432869	0.790563	1.965262	2.9994902	0.1755105	0.4697282
	-4	0.1462064	14.840484	12.531717	0.0113336	0.082635	11.065426	9.4074546	1.6493283	30.085688	9.9655916	6.9084878	2.5267644	0.7788842
	-3	17.783834	0.1078869	0.0679994	15.860666	0.0958291	0.1256022	0.1768875	22.086735	1.2207351	13.236069	18.513609	2.4805212	8.2436259
	-2	0.0315022	16.305141	2.9516739	0.070671	7.9515284	7.7908637	6.7009685	0.0727944	1.6227186	19.032891	13.431796	18.536278	5.501174
	-1	18.372772	0.0393659	0.0689799	1.8852516	0.0296239	8.303919	9.9328751	8.9347834	0.5230001	5.4405518	7.84639	8.773941	29.848546
**160**	0	0.0077426	16.34653	0.0064961	0.0741809	12.62315	0.0121105	0.0023963	1.2974917	24.447226	0.0002723	0.0057766	34.850515	10.326113
	1	18.332484	0.0402873	0.0684113	1.9375914	0.0299259	8.2914052	9.9190734	8.9530479	0.5247806	5.447591	7.8244329	8.8457737	29.785196
	2	0.028084	16.206814	3.0936187	0.0704839	7.8760195	7.8127468	6.7199572	0.0728614	1.613215	19.001885	13.456361	18.594635	5.4533189
	3	17.666919	0.1122673	0.0663259	15.978681	0.0968894	0.1222984	0.1727506	22.075824	1.218765	13.185493	18.572667	2.4718279	8.2592916
	4	0.1354729	14.655192	12.729192	0.0113608	0.072507	11.05459	9.3995318	1.6498245	30.099321	9.9444123	6.9401082	2.4969935	0.8114936
	5	13.25874	0.2602437	0.0289946	32.06524	0.0869042	14.181396	17.260451	16.450338	0.7924046	1.9678753	3.0014907	0.158423	0.4875002
	6	0.4062778	10.292985	34.15467	0.0207512	35.608093	8.5563014	6.5466967	0.162103	3.5339584	0.4078969	0.248255	0.0427541	0.0192574
	-6	18.304158	7.0845347	31.688102	3.4866925	25.039597	6.2553899	4.6214211	1.1642845	1.7336612	0.2649605	0.2970493	0.0498897	0.0102587
	-5	2.9241345	12.482525	3.0240378	31.48145	1.1468886	12.330546	16.4828	9.4333449	5.6405073	2.3224114	2.1123008	0.0916864	0.5273678
	-4	12.605134	3.580848	6.9745479	0.3890392	1.9964843	15.29113	9.1697524	11.757088	19.184967	7.5139614	8.3500697	2.7164721	0.4705058
	-3	2.4584112	12.362589	3.5723651	13.023493	0.3160525	0.2102096	0.8616505	16.100387	9.1670367	16.564571	14.873279	1.4999934	8.9899628
	-2	9.6159238	1.8000073	1.2089062	0.0394986	12.997481	9.2164602	5.2825171	0.6985285	1.6847641	16.247798	17.305199	20.473411	3.4295047
	-1	1.6692564	10.616479	3.4583995	1.3979989	0.0359993	6.6242163	13.57249	6.1871693	3.4248427	7.1130891	7.0179532	5.7485419	33.133564

**150**	0	8.3159461	1.2543052	0.1143221	0.0080238	16.899654	0.0846966	0.0000903	9.2948441	18.311966	0.0285955	0.0016594	38.889495	6.7964019
	1	1.5591727	10.717139	3.4328453	1.4445302	0.0362211	6.6141532	13.555125	6.2005225	3.4346903	7.127702	6.9864529	5.7059995	33.185447
	2	9.2952279	2.0827716	1.2742976	0.039898	12.930323	9.2476736	5.297487	0.6957167	1.6753852	16.228616	17.318315	20.446691	3.4675975
	3	2.1119281	12.672706	3.4954897	13.144502	0.3167575	0.2060822	0.8519807	16.095326	9.1595207	16.519069	14.929309	1.4976738	8.9996557
	4	11.864907	4.206189	7.1037681	0.399542	1.9504368	15.286757	9.1639088	11.763388	19.195727	7.4857101	8.3897619	2.7301828	0.4597225
	5	2.4327023	12.907347	2.9201499	31.58493	1.1453159	12.353844	16.503666	9.4433599	5.6495489	2.3177819	2.121607	0.0986337	0.5211138
	6	16.843098	8.2325596	31.732768	3.5604027	25.188789	6.278841	4.6371116	1.1660417	1.7373837	0.2657341	0.2970437	0.051329	0.0088979
	-6	27.590513	13.729849	27.742362	1.5307144	19.191928	4.8617618	2.9128969	0.9898978	0.980186	0.173552	0.2423378	0.0469907	0.0070097
	-5	0.1634807	12.923844	6.583198	34.937013	1.8610069	10.344116	16.414561	6.943937	5.5962679	2.0631032	1.5972708	0.0943293	0.4778727
	-4	11.909306	2.9367041	4.2539052	0.0579558	4.8871896	18.191362	10.24517	13.947163	16.101754	6.2679761	8.1766503	2.5870747	0.43779
	-3	0.232251	10.838949	5.8231791	11.582805	0.4323518	0.6034849	1.5294573	15.199515	12.504755	17.703785	13.341207	1.470618	8.7376416
	-2	7.3987164	0.6432464	0.6392281	0.452105	15.032044	8.9531961	4.7875351	1.2224946	1.9912486	15.404436	19.712276	20.413284	3.3501907
	-1	0.2483089	8.647982	4.661842	1.0476429	0.0835445	6.9935104	14.10034	5.3599375	4.3477585	8.4053059	6.8936858	5.5666453	33.643496
**140**	0	6.1145687	0.170631	0.1414778	0.5465362	17.043445	0.0536065	0.0097231	12.649303	16.939828	0.0253818	0.0047451	39.669562	6.6311921
	1	0.2461791	8.6406374	4.6479646	1.079393	0.0845586	6.9842692	14.082858	5.3746116	4.3593592	8.3999185	6.88001	5.5909912	33.62925
	2	7.3248029	0.6846152	0.6871335	0.4509223	14.980577	8.9840599	4.8023248	1.2161321	1.9821569	15.386715	19.725024	20.424438	3.3510994
	3	0.2263417	10.805904	5.7748356	11.677003	0.4358327	0.5970696	1.5163925	15.193964	12.49849	17.682222	13.376104	1.4434007	8.7724405
	4	11.668098	3.0541294	4.4001197	0.056641	4.8347702	18.190254	10.242241	13.95742	16.111593	6.2544807	8.2024958	2.5631484	0.4646093
	5	0.1550162	12.850378	6.5003375	35.036321	1.8633024	10.363512	16.435371	6.9537399	5.6043667	2.0599451	1.6048541	0.0840768	0.4887798
	6	26.722417	14.073131	28.144416	1.5449475	19.26945	4.8797986	2.9211293	0.9918847	0.9822363	0.1731784	0.2433405	0.0454422	0.0086285
	-6	22.333828	0.1900205	40.745732	1.6744831	24.151376	4.2189938	4.4088058	1.3723559	0.5298631	0.0731377	0.2582947	0.0380587	0.0050512
	-5	0.4592111	17.773311	0.2792613	34.622366	2.2091849	14.676875	13.732082	4.5209799	7.8715953	2.4678023	0.8732284	0.174648	0.3394552
	-4	12.755617	0.7544466	7.4168654	0.5845906	3.1940852	14.3111	13.62285	19.279476	11.109747	3.8232461	10.240829	2.4782695	0.4288783
	-3	0.7049094	16.126072	0.1310232	11.010054	0.6356241	1.1165448	1.137214	10.910974	17.0119	22.515195	8.5786484	2.8633382	7.2585035
	-2	9.2956414	0.9669289	1.1597738	0.7009661	12.079068	6.9002797	6.1524433	1.8706461	1.5845169	10.282326	25.194839	19.376146	4.4364264
	-1	0.6663488	13.835731	0.0144849	1.0322306	0.5355471	8.7556154	10.639475	3.5397653	6.0572378	10.724865	4.8443733	9.3797641	29.974562
**130**	0	8.1666841	0.9053797	0.011854	0.6013498	14.379742	0.0015822	0.6000805	16.995464	11.643226	0.1965563	0.0389501	36.173911	10.28522
	1	0.6648623	13.819265	0.0156548	1.0565523	0.5363228	8.7461576	10.622856	3.5446971	6.0800075	10.759999	4.7887182	8.0524835	31.312423
	2	9.249538	0.9637852	1.2226418	0.7033954	12.030908	6.9211926	6.1721604	1.8652986	1.5729814	10.307499	25.165397	17.677656	6.1475473
	3	0.7001411	16.067408	0.1343931	11.075551	0.638636	1.1099128	1.1253827	10.909712	16.999159	22.508929	8.6010677	1.689148	8.4405601
	4	12.621131	0.74889	7.5734389	0.5864569	3.1487652	14.30738	13.614182	19.29055	11.120911	3.8042317	10.274002	2.0011029	0.9089592
	5	0.4536336	17.662774	0.2816398	34.670305	2.2086468	14.700273	13.750042	4.5249304	7.8872111	2.4637058	0.8821183	0.0673484	0.4473713
	6	21.928454	0.1859872	41.013237	1.6816997	24.252095	4.2340945	4.4224262	1.3751511	0.5316451	0.0725073	0.2595347	0.0281265	0.0150418
	-6	31.1536	27.450094	17.065167	16.556892	2.4737808	1.0898886	2.7182198	0.9890739	0.2810902	0.0657253	0.1476238	0.0070315	0.0018143
	-5	9.4399716	11.065186	13.763206	17.547059	16.431587	18.328572	3.3173165	1.3552364	5.9841638	2.0631495	0.4376399	0.1432815	0.1236315
	-4	5.7131399	4.1974313	4.557428	6.8298139	8.9259619	10.752872	20.06274	22.352345	2.4496478	1.3524817	10.503481	1.8837081	0.4189491
	-3	2.4703288	2.7058199	6.5496109	4.1030757	4.122983	6.9490711	4.7204689	5.6963572	24.054021	28.031157	1.0023516	4.3571877	5.2375678
	-2	2.0350914	1.0969636	2.8372575	3.1222245	9.5787303	3.9343174	6.0102911	7.7283696	4.5486857	1.3099127	33.743718	19.850453	4.2039851
	-1	0.8598016	0.9670054	4.7425678	0.7688522	3.5268571	8.649397	9.7461407	2.5269337	9.5526545	14.477974	4.058523	11.683125	28.440169
**120**	0	1.223209	0.4888115	1.5411684	1.5981055	10.033225	0.4697515	6.8967889	18.671692	6.2398613	5.541141	0.0458131	36.574398	10.676035
	1	0.8216724	1.0074033	4.7245336	0.7888209	3.5180121	8.6565674	9.7278369	2.5183505	9.584166	14.747399	3.7666893	7.5413763	32.597173
	2	1.9191342	1.2179568	2.8108064	3.1506069	9.5447605	3.9558986	6.017724	7.7305363	4.5270952	1.3470644	33.705831	15.090567	8.9820194
	3	2.2533734	2.930765	6.4811342	4.1670426	4.1109019	6.9533096	4.6964945	5.7087442	24.034532	27.810334	1.2480104	1.6915207	7.9138374
	4	5.2618304	4.6579264	4.4935718	6.8862075	8.8655472	10.774019	20.042939	22.380606	2.4578582	1.1724906	10.701051	1.1206793	1.1852734
	5	8.5003478	12.014463	13.536851	17.713319	16.396893	18.392923	3.3178117	1.3505546	6.0030693	2.0199534	0.4864517	0.0517003	0.2156622
	6	28.3485	30.200174	16.896697	16.767981	2.4707605	1.0934128	2.7252282	0.9911998	0.2831547	0.0612187	0.1528172	0.0049722	0.003884

Table S8. CASSCF-SO percentage wave function composition angular dependence for**2-Tm** when the quantization axis is oriented along the direction bisecting the N-Tm-N angle in a 0.1 T applied field.

N-Tm-N angle / °	M_J	WF 1	WF 2	WF 3	WF 4	WF 5	WF 6	WF 7	WF 8	WF 9	WF 10	WF 11	WF 12	WF 13
	**-6**	0.000171	0.0002879	0.0000415	0.0016683	0.0010637	0.0051611	0.0050246	0.0083634	0.0031286	0.044555	0.0009997	99.927825	0.0017104
	**-5**	0.0023759	0.0042081	0.0046639	0.004336	0.0040428	0.015055	0.0147492	0.0796575	0.0293153	97.601941	2.1928852	0.0467697	0
	**-4**	0.0148926	0.0075236	0.0127425	0.0675629	0.0632539	0.0434044	0.041214	73.07841	26.549137	0.109591	0.0024591	0.0098087	0
	**-3**	0.3933966	0.0397494	0.8472683	0.2658117	0.0896964	50.310625	47.931849	0.0617429	0.023441	0.0247843	0.0006011	0.0110339	0
	**-2**	0.4006768	11.701148	4.376259	43.695843	39.568059	0.0554905	0.0532268	0.0733578	0.0587752	0.014115	0.0003613	0.0026883	0
	**-1**	48.183188	1.3868008	44.335807	4.1525402	0.4121276	1.2150931	0.291736	0.0080299	0.0071906	0.0060075	0.0014037	0.000075	0
**180**	**0**	2.1568339	73.733028	0.7464451	3.9382903	19.352161	0.0110259	0.0225807	0.0315133	0.007356	0.0003593	0.0002326	0.0000864	0.0000878
	**1**	48.048676	1.4087449	44.446167	4.1505866	0.4174142	1.1785155	0.3272003	0.001593	0.0136184	0.000172	0.0072367	0	0.000075
	**2**	0.39091	11.6664	4.3660302	43.386065	39.932747	0.0518855	0.0567256	0.0161535	0.115924	0.0002802	0.014191	0	0.0026875
	**3**	0.3914383	0.0401553	0.8471247	0.2643595	0.0904876	47.05427	51.190602	0.021994	0.0631587	0.0005163	0.0248623	0	0.0110311
	**4**	0.0148911	0.0074818	0.0127402	0.0669748	0.063791	0.0405878	0.0439998	26.587383	73.040318	0.0024672	0.1095579	0	0.0098066
	**5**	0.002379	0.0041862	0.0046687	0.0043044	0.0040813	0.0140634	0.0157316	0.0288008	0.0801482	2.1942089	97.600667	0	0.0467601
	**6**	0.0001707	0.0002865	0.0000416	0.001658	0.0010741	0.0048224	0.0053612	0.0030004	0.0084889	0.0010027	0.0445427	0.0017111	99.92784
	-6	0.0001748	0.0002682	0.000123	0.0014477	0.0010257	0.0077038	0.0067298	0.0103128	0.0001128	0.0209628	0.0002112	99.950327	0.0006001
	-5	0.0040096	0.0023679	0.0032473	0.0037824	0.0047173	0.013409	0.0103753	0.0344443	0.0004104	98.902655	0.9989773	0.0216043	
	-4	0.0019881	0.00792	0.0028447	0.0615894	0.0645662	0.0130742	0.0123155	98.726069	1.0637352	0.0357867	0.0003754	0.0097352	
	-3	0.1720373	0.0312427	1.1525929	0.0891729	0.0296086	53.048559	45.416184	0.0246393	0.000172	0.0207347	0.0002073	0.0148488	
	-2	0.2785735	8.9523796	1.4250306	47.892147	41.304022	0.0197788	0.0095304	0.102675	0.002637	0.0103781	0.0001415	0.0027072	
**170**	-1	49.050458	1.0546363	46.977342	1.1921704	0.3208642	1.3072757	0.0861316	0.000387	0.0016426	0.0083126	0.0006354	0.0001434	
	0	1.1250058	79.924738	0.7612514	1.793862	16.23221	0.0245725	0.0715652	0.0308878	0.0345987	0.0007176	0.0005264	0.0000323	0.0000325
	1	48.91583	1.0651947	47.099341	1.1939824	0.3217291	1.2382413	0.1545628	0.0017498	0.0002815	0.0000445	0.0088997	0.0001434	
	2	0.2748529	8.9194095	1.4203075	47.616923	41.620732	0.0177414	0.0115497	0.0029623	0.1022998	0.0000742	0.0104417	0.0027065	
	3	0.1708887	0.0312916	1.1517136	0.0886457	0.0297639	44.281116	54.185997	0.0003779	0.0244243	0.0002116	0.0207245	0.0148454	
	4	0.0019991	0.0079311	0.0028285	0.0610844	0.0649805	0.0108206	0.0145593	1.0650491	98.72486	0.0003486	0.0358053	0.009733	
	5	0.0040084	0.0023535	0.0032542	0.0037541	0.0047477	0.0112898	0.0124869	0.0003354	0.0345117	0.9995615	98.902097	0.0215999	
	6	0.0001747	0.0002667	0.0001233	0.0014397	0.0010336	0.0064173	0.0080132	0.0001099	0.0103133	0.0002123	0.0209574	0.0006004	99.950338
	-6	0.0001358	0.0001915	0.000057	0.001509	0.0014152	0.0082551	0.0079709	0.0031655	0.0013198	0.0009975	0.000016	99.973367	0.0016002
	-5	0.0071785	0.0005529	0.0042882	0.003165	0.0046232	0.0009282	0.0009827	0.0071285	0.0030995	98.378085	1.5889337	0.0010342	0
	-4	0.0079767	0.038794	0.0049621	0.0070194	0.0061785	0.0072438	0.0079489	69.763702	30.14129	0.0100956	0.0001804	0.004609	0
	-3	0.0108508	0.1205637	0.5729651	0.0033736	0.0235201	51.216541	48.020867	0.0092087	0.0045405	0.0015528	0.0000119	0.0160037	0
	-2	0.0654633	3.6961789	0.2569177	50.080777	45.86483	0.0144549	0.0036307	0.0057065	0.0021834	0.006594	0.0001461	0.0031177	.0
	-1	49.101515	0.8286944	48.896216	0.0564999	0.5463659	0.4082207	0.1383218	0.0030064	0.0084533	0.0114355	0.0010483	0.0002217	.0
**160**	0	1.7901739	90.644973	0.3621027	0.0712819	6.7079305	0.0320642	0.2952436	0.0040285	0.0903077	0.0010367	0.0007678	0.0000442	0.0000449
	1	48.924669	0.8301743	49.068101	0.0594142	0.5464177	0.4127535	0.1343129	0.0002615	0.0111944	0.0000327	0.0124455	0	0.0002218
	2	0.0659136	3.6798442	0.2534282	49.702078	46.2629	0.0141721	0.003918	0.0048362	0.0030549	0.000075	0.0066625	0	0.0031168
	3	0.0108808	0.1205049	0.5716295	0.0033046	0.0235195	47.870059	51.368794	0.0037726	0.0099709	0.0000425	0.0015217	0	0.0160002
	4	0.0079471	0.0387872	0.0049705	0.0069503	0.0062184	0.0067334	0.0084517	30.190717	69.714343	0.0001473	0.0101263	.0	0.0046077
	5	0.0071594	0.0005501	0.0043048	0.0031307	0.0046539	0.000868	0.0010422	0.0030778	0.0071477	1.5898887	98.377143	.0	0.001034
	6	0.0001357	0.0001906	0.0000574	0.0014968	0.0014268	0.0077059	0.0085159	0.0013889	0.0030954	0.0000162	0.0009971	0.0016013	99.973372
	-6	0.0001608	0.0001324	0.0000997	0.0023831	0.0025769	0.0060653	0.0059051	0.0006644	0.0005224	0.0011352	0.0000997	99.974246	0.0060093
	-5	0.0001392	0.0064503	0.0098876	0.0022505	0.0024608	0.02717	0.0316784	0.0462188	0.0330683	91.77783	8.0617691	0.0010771	0

	-4	0.0711845	0.0109337	0.0190702	0.0777731	0.1459423	0.0845385	0.0860734	58.202503	41.226223	0.0686982	0.0061491	0.0009112	0
	-3	0.1320234	0.7038631	0.060775	0.045398	0.0852185	50.503823	48.250506	0.0887505	0.0625359	0.0495574	0.0049379	0.0126098	0
	-2	3.1378908	0.1743065	0.2038715	47.031293	49.0786	0.0832112	0.0562103	0.0835507	0.1388421	0.0068038	0.0005828	0.0048357	0
	-1	3.7966723	45.354809	49.451778	0.4092651	0.3173896	0.0072731	0.59063	0.0245112	0.0258831	0.0162085	0.0054141	0.0001645	0
**150**	0	85.753007	7.6694609	0.3259359	5.5202527	0.0638415	0.0854392	0.4468052	0.0094757	0.1238705	0.0003721	0.001254	0.0001404	0.0001446
	1	3.7772291	45.184888	49.637344	0.4146762	0.315624	0.0057706	0.5923301	0.016511	0.0338495	0.0000604	0.0215522	0	0.0001644
	2	3.1280591	0.1757298	0.2011826	46.370982	49.750204	0.0811164	0.0582169	0.0494575	0.1728335	0.0006211	0.0067622	0	0.0048349
	3	0.1322083	0.7020518	0.0609292	0.0446571	0.0859264	49.00154	49.754385	0.0631487	0.0880694	0.0038929	0.0505835	0	0.0126076
	4	0.0711238	0.0108249	0.0191153	0.076503	0.147113	0.0819035	0.0886168	41.381949	58.047114	0.0059429	0.0688825	0	0.0009113
	5	0.0001412	0.006418	0.0099101	0.002218	0.0024927	0.0262675	0.0325575	0.032796	0.0464661	8.0687779	91.770878	0	0.0010768
	6	0.0001605	0.0001316	0.0001002	0.0023481	0.0026104	0.0058815	0.0060856	0.0004644	0.000722	0.0000998	0.0011347	0.0060141	99.974247
	-6	0.0000595	0.0001698	0.0001686	0.0022268	0.0021005	0.0027475	0.0027132	0.0053806	0.0048606	0.0313555	0.0049955	99.92916	0.0140618
	-5	0.0001283	0.0009928	0.0071694	0.0072128	0.0056723	0.1768788	0.1925484	0.0176916	0.01517	85.856443	13.683346	0.0367416	0.0000052
	-4	0.0213135	0.0409908	0.065358	0.4657324	0.6837375	0.0250855	0.0261699	53.922208	44.702649	0.0326635	0.0053627	0.0087266	0
	-3	0.3305738	0.8511588	0.7346701	0.0047247	0.0161879	49.844042	47.819976	0.0263521	0.0228253	0.2931526	0.0509517	0.0053832	0
	-2	0.7873042	0.1270578	0.5387238	49.655237	47.76146	0.00779	0.0004577	0.4556478	0.6475307	0.011671	0.0017522	0.0053651	0
	-1	0.5137923	48.878998	48.216565	0.1368041	0.584764	0.4522674	1.0670841	0.0651232	0.0581791	0.0157106	0.0104143	0.000298	0
**140**	0	96.698548	1.0173869	0.0623955	0.1318498	1.2090057	0.1702249	0.593111	0.0138715	0.0987484	0.0020814	0.0022754	0.0002453	0.0002566
	1	0.5114636	48.071795	49.024722	0.1415458	0.5826017	0.4365263	1.0817152	0.0548158	0.0684046	0.0001847	0.0259275	0	0.0002981
	2	0.7853749	0.1304512	0.532264	48.982744	48.439546	0.0077932	0.000466	0.3593952	0.7431832	0.0019426	0.0114743	0	0.0053651
	3	0.3299553	0.8397604	0.7444465	0.0046789	0.0161995	48.67711	48.989346	0.0219166	0.0272346	0.04381	0.3001592	0	0.005383
	4	0.0212993	0.0401177	0.0661403	0.4579245	0.6908367	0.024458	0.02676	45.038413	53.587309	0.0050983	0.0329164	0	0.0087263
	5	0.000128	0.0009538	0.0072064	0.007123	0.0057586	0.1723949	0.196875	0.0147281	0.0181248	13.700883	85.839086	0.0000051	0.0367327
	6	0.0000596	0.0001673	0.0001709	0.0021962	0.0021298	0.0026814	0.0027776	0.0044571	0.0057803	0.0050038	0.0313385	0.0140739	99.929164
	-6	0.0001951	0.0001144	0.0002447	0.0010511	0.0008365	0.0034508	0.003478	0.0058968	0.0049144	0.8791522	0.0787674	99.011703	0.0101958
	-5	0.0015569	0.004077	0.0050923	0.0422385	0.042646	0.207441	0.2172919	1.3541544	1.0969117	88.191031	7.9057829	0.9316811	0.0000955
	-4	0.0566524	0.1184812	0.1351876	0.5125537	0.6398188	1.2172381	1.2124754	51.8842	42.017581	1.9873746	0.1763497	0.0420813	0.000006
	-3	0.3425594	0.8776023	0.1387834	0.9639043	1.032684	47.935168	46.080683	1.0614599	0.8012998	0.696063	0.067933	0.00186	0
	-2	0.0219611	0.6088325	1.9603178	48.580197	45.69501	0.7632753	0.729031	0.7355296	0.8973764	0.0056973	0.0007517	0.0020186	0
	-1	2.5966217	46.88485	46.722822	0.2000549	2.2391366	0.4415741	0.7783863	0.0715259	0.0551571	0.0053007	0.004385	0.0001796	0.000006
**130**	0	93.973008	3.2656184	1.8159623	0.067293	0.0267738	0.1874292	0.6328578	0.0010362	0.0268612	0.0017898	0.0010288	0.0001676	0.0001742
	1	2.5854652	46.639303	46.980927	0.1901256	2.2487876	0.4260187	0.792904	0.0591126	0.0674881	0.0002353	0.0094472	0	0.0001808
	2	0.0220938	0.6039556	1.9590304	47.945227	46.337873	0.7430135	0.7484093	0.5713323	1.0605984	0.000416	0.0060305	0	0.0020191
	3	0.3416471	0.8751596	0.140668	0.9497569	1.0454897	46.685941	47.333879	0.8706987	0.9912604	0.0579988	0.7056413	0	0.0018594
	4	0.0564925	0.1178265	0.1356226	0.504918	0.6469145	1.1843624	1.2441708	42.277334	51.62737	0.1798411	1.9830768	0	0.0420683
	5	0.0015523	0.0040659	0.0050961	0.041641	0.043181	0.2017308	0.2228661	1.1029301	1.3471625	7.9161724	88.182069	0.0000965	0.9314359
	6	0.0001948	0.0001138	0.0002452	0.001038	0.0008488	0.0033569	0.0035681	0.0047897	0.0060192	0.0789281	0.8787364	0.0102041	99.011957
	-6	0.0003138	0.001002	0.0039019	0.0246662	0.0353342	0.1117265	0.1321744	0.4915411	0.4664023	7.6718394	5.1972075	85.457808	0.4060832
	-5	0.0518155	0.0106489	0.0660276	0.9309539	1.4887133	0.156543	0.1545038	19.345336	18.240205	28.502589	19.554533	11.444761	0.0533705
	-4	0.0387995	0.9618576	3.1303677	0.598888	1.941724	17.024241	16.617592	18.576492	17.419305	13.23738	8.3589427	2.0835166	0.0108938
	-3	3.6745292	0.1310639	2.2841251	16.999654	12.482727	23.888702	19.213224	4.6489089	2.9369006	7.6238775	5.6891236	0.4254988	0.0016655
	-2	0.9584174	15.998052	18.398568	22.801754	18.664767	3.2711576	1.8600669	6.6943181	8.1052183	2.0256832	1.1177372	0.1029755	0.0012857
	-1	33.420673	11.23412	26.094381	8.3695996	1.8223438	4.8514133	11.298214	1.1363801	0.8428773	0.4017204	0.5189742	0.009118	0.0001849
**120**	0	23.762124	43.379179	0.0000105	0.669487	26.961013	1.8544868	0.9788354	0.2331962	1.9557521	0.1542065	0.0460402	0.00245	0.0032191
	1	33.381694	11.218125	26.150098	8.3770957	1.827056	4.772636	11.367441	1.0975477	0.8795733	0.2366787	0.6827693	0.0007036	0.0085818
	2	0.956541	15.963966	18.394933	22.743769	18.771174	3.2493358	1.8846354	6.3877097	8.4042855	1.4262995	1.713228	0.0001122	0.1040109
	3	3.6646849	0.1317556	2.2827556	16.937447	12.531935	23.697765	19.434236	4.4992067	3.088767	5.0775332	8.2272346	0.0025064	0.4241733

	4	0.03844	0.9586643	3.1253554	0.5965051	1.9465436	16.856131	16.769288	17.851762	18.175249	9.1088389	12.48028	0.0089732	2.0839698
	5	0.0516559	0.0105681	0.0655902	0.9256648	1.4912907	0.1553424	0.1564865	18.566419	19.000342	19.323872	28.758808	0.0553935	11.438568
	6	0.0003122	0.000998	0.0038857	0.0245162	0.0353777	0.1105206	0.133302	0.4711839	0.4851227	5.2094827	7.6551217	0.4061835	85.463993

Table S9. CASSCF-SO percentage wave function composition angular dependence for **2-Tm** when the quantization axis is oriented along the direction perpendicular to the N-Tm-N plane in a 0.1 T applied field.

N-Tm-N angle / °	M_J	WF 1	WF 2	WF 3	WF 4	WF 5	WF 6	WF 7	WF 8	WF 9	WF 10	WF 11	WF 12	WF 13
	**-6**	39.708521	35.676486	3.3653048	3.7608628	5.6388706	0.0191943	8.2652289	2.9345136	0.0260197	0.0936946	0.4641919	0.0078716	0.0392405
	**-5**	2.2471427	2.2774052	18.691767	30.407712	0.6194496	25.604343	0.0848777	0.1449584	14.940237	3.6106471	0.8392672	0.4139681	0.1182254
	**-4**	7.7506077	6.2078492	0.5864169	0.1805764	7.8787744	0.310034	25.79682	31.780095	0.246311	3.1956229	13.073519	0.7184251	2.274949
	**-3**	1.2541298	0.9195325	15.222687	12.922243	0.2259876	1.4496117	0.0732038	0.1455715	25.396464	26.04863	5.9721726	8.4214503	1.9483167
	**-2**	1.3964841	1.392277	0.3016946	0.0228605	22.047623	0.1983433	15.316942	2.068259	0.1136422	5.9140192	27.191369	5.8602052	18.176282
	**-1**	0.657174	0.1408828	12.267392	2.1225338	0.0432795	22.396325	0.4005475	0.1219997	9.2284373	11.120782	2.4102295	31.158833	7.9315843
**180**	**0**	0.0886061	0.6613461	0.2980082	0.0417407	27.174083	0.049423	0.0496254	25.599095	0.0748083	0.0857137	0.0440212	9.9557168	35.877813
	**1**	0.6256165	0.1754319	12.156375	2.2420282	0.0440095	22.369515	0.4025697	0.1219173	9.2467907	11.210891	2.3062868	30.032478	9.0660901
	**2**	1.2335409	1.5641038	0.301374	0.0233834	21.996326	0.1997304	15.363203	2.0587169	0.1136182	5.9289442	27.170541	4.6147412	19.431777
	**3**	1.1236655	1.0593943	14.860374	13.282284	0.2291054	1.4348631	0.0734748	0.1455588	25.386152	25.966455	6.0624191	7.7972316	2.5790232
	**4**	6.90021	7.0714429	0.5803848	0.1915382	7.8334021	0.3085734	25.798376	31.79536	0.2464265	3.1307556	13.147666	0.5864409	2.4094233
	**5**	1.9747933	2.5632526	18.076639	30.946501	0.623232	25.642119	0.0869297	0.145111	14.955018	3.5995838	0.8540763	0.4196312	0.113113
	**6**	35.039508	40.290596	3.2915823	3.8557372	5.6458585	0.0179249	8.2882016	2.9388441	0.0260754	0.0942606	0.4642407	0.0130073	0.0341629
	-6	0.8211719	5.5958617	30.450688	0.877702	40.101752	15.825354	1.5671483	0.4600628	3.559157	0.5603295	0.1213588	0.0157651	0.0436491
	-5	11.180947	0.8647044	1.1162127	29.065097	0.1257634	3.4981438	29.900618	15.988181	2.405628	1.0533665	4.1552906	0.4371409	0.2089066
	-4	0.7209908	13.710718	13.167321	0.7727154	0.2470071	16.105598	2.3112133	4.539904	27.698875	13.598042	3.7804988	1.1389494	2.2081676
	-3	17.288789	0.6398736	1.0880096	15.792076	0.113571	0.0221623	0.0516247	19.098795	3.1685449	7.1267657	24.78058	6.9669774	3.8622312
	-2	0.4344296	18.714447	3.4692073	0.9604863	4.7795106	12.389424	1.8072757	0.179969	1.2224083	24.840193	7.0941976	8.7440456	15.364406
**170**	-1	19.63967	0.3949982	0.7700064	1.9802781	0.0221578	2.1218829	14.344383	7.9196569	1.4050572	2.8318383	10.053054	24.586336	13.930681
	0	0.3321345	20.180394	0.000577	0.8904489	8.948787	0.0003739	0.0116675	3.6107215	21.060872	0.0002774	0.0066582	16.277854	28.679235
	1	19.584457	0.4154117	0.7637444	2.0332663	0.0230188	2.1247075	14.316419	7.9315541	1.4113502	2.8364282	10.034563	24.538732	13.986348
	2	0.4035146	18.672526	3.5716166	0.9702759	4.7081799	12.423893	1.8093152	0.1805825	1.2137627	24.824107	7.1037133	8.7050734	15.41344
	3	17.13943	0.6975587	1.0706132	15.90125	0.1154178	0.021776	0.0487159	19.090521	3.1639929	7.1080131	24.806746	6.9695014	3.8664637
	4	0.6653478	13.649058	13.248233	0.7809929	0.2727	16.090701	2.3032969	4.5385569	27.712865	13.59936	3.7889954	1.1553828	2.1945109
	5	11.015321	0.9286602	1.103308	29.109626	0.1258644	3.4986239	29.945616	16.001206	2.4119088	1.0581809	4.1549697	0.4466981	0.2000179
	6	0.7737968	5.5357889	30.180463	0.8657858	40.41627	15.87736	1.5827061	0.4602898	3.5655784	0.5630992	0.1193742	0.0175444	0.0419432
	-6	43.230379	28.601078	0.1298654	0.1208082	12.518448	3.6433471	7.4617265	0.1475483	3.4372813	0.1337779	0.5226723	0.0364066	0.0166609
	-5	0.077768	0.0281544	17.621146	32.613059	0.0529706	18.356437	9.2842013	15.663994	0.8944957	3.7101465	1.1315749	0.1300147	0.4360383
	-4	11.388308	8.7734661	0.0717579	0.0025711	4.6993419	7.7004402	15.791266	1.6385384	30.241613	3.9693931	12.680768	2.3521939	0.6903423
	-3	0.0463245	0.0744276	17.218598	15.182393	0.1544234	0.6364291	0.2662986	23.162209	1.1410509	24.351143	7.4382436	2.3206652	8.0077934
	-2	2.5233267	3.6392977	0.1941158	0.0022347	19.675699	4.9853944	10.46976	0.0869917	1.8237777	7.4884771	25.202824	18.516028	5.3920732
	-1	0.0121627	0.1333434	15.007798	1.7158212	0.1869622	14.66295	6.6868178	8.7116133	0.4168683	10.346575	3.0215872	9.080466	30.017035
**160**	0	0.2904169	2.6784644	0.2780163	0.000029	25.50192	0.008767	0.0298735	1.1555619	24.075962	0.0011635	0.0001811	35.312812	10.666832
	1	0.0107708	0.1346601	14.921458	1.8146073	0.1873102	14.644668	6.6786343	8.730004	0.4185204	10.334645	3.0187352	8.9727844	30.133202
	2	1.6755654	4.5059283	0.1944919	0.0019153	19.614723	4.9996747	10.503276	0.0866011	1.8140186	7.4869518	25.198372	18.425292	5.4931902
	3	0.0391225	0.081528	16.945636	15.463067	0.1552678	0.6281719	0.2625429	23.149355	1.1416591	24.358401	7.4400616	2.3009531	8.0342335
	4	8.3437282	11.841403	0.0724794	0.0024358	4.6546924	7.6970133	15.787037	1.6381766	30.257091	3.9719787	12.688786	2.3716324	0.673547
	5	0.064822	0.0410917	17.215666	32.957465	0.0536633	18.381579	9.2963999	15.681295	0.8950723	3.7134755	1.1328041	0.1419849	0.4246825
	6	32.297305	39.467157	0.1289708	0.1235934	12.544578	3.655129	7.4821656	0.1481111	3.4425902	0.1338729	0.5233902	0.0387675	0.014369
	-6	25.291906	24.346416	8.1212359	0.3860034	21.713264	4.015154	10.500446	1.3222744	3.399337	0.3412973	0.4999749	0.0490879	0.0136032

	-5	2.2848817	4.1480275	9.1255596	27.843304	0.5786895	20.458027	10.560348	12.347421	6.4929699	2.9767794	2.5624762	0.0997688	0.5217486
	-4	13.105875	9.0315291	2.3732157	1.140682	1.2326174	8.0142053	12.243214	11.404209	20.748461	8.4121923	9.1201351	2.7211826	0.452482
	-3	1.0676362	3.5468348	14.418176	15.840364	0.5394929	0.1354073	0.1020751	14.319207	7.8479458	16.440139	15.276509	1.4339545	9.0322585
	-2	8.1778959	2.1881402	0.4896409	1.7480578	14.905787	6.0643476	9.4789587	0.5473077	0.8737475	15.276156	16.345161	20.685412	3.2193875
	-1	0.350053	3.0565304	15.639125	1.9382147	0.0722037	11.277923	7.0948403	5.6946216	3.2329756	6.5632987	6.1767274	5.285543	33.617944
**150**	0	6.6027311	0.3334905	0.0174221	1.8735674	21.923139	0.0388162	0.0001345	8.7129043	14.783812	0.0093919	0.0008243	39.528672	6.1750944
	1	0.2729246	3.1375424	15.578823	2.005913	0.0752579	11.260481	7.0828689	5.7060272	3.2437951	6.5655002	6.1583054	5.2398625	33.672698
	2	7.504895	2.8735365	0.5027828	1.7502365	14.838286	6.0830156	9.5056936	0.5453879	0.8655345	15.273656	16.340989	20.654335	3.2616517
	3	0.8114286	3.8126262	14.253447	15.9929	0.5461486	0.1322791	0.099435	14.315131	7.835828	16.431908	15.295269	1.4249403	9.0486589
	4	11.358755	10.768053	2.3941924	1.147424	1.2046461	8.0100241	12.22719	11.409989	20.757656	8.401643	9.143754	2.7266775	0.4499952
	5	1.836481	4.6015643	8.9533354	27.937617	0.5825451	20.485441	10.57236	12.355171	6.5078221	2.9693051	2.576098	0.101363	0.5208971
	6	21.334537	28.155709	8.1330442	0.3957175	21.787922	4.024879	10.532436	1.3203488	3.4101159	0.3387332	0.5037758	0.0492013	0.0135806
	-6	17.522943	19.461767	12.144963	0.6885406	25.942065	5.5241991	11.536512	2.3153839	3.7007536	0.560709	0.5299471	0.057708	0.0145092
	-5	0.2497241	4.5332452	5.4160592	26.446096	0.9159911	21.55261	11.965278	11.930544	9.7740524	2.7946651	3.7371978	0.1360365	0.5485006
	-4	13.902058	8.8178745	5.1708444	0.1376508	0.1214187	6.5672638	10.418465	14.756624	17.884474	10.962568	7.9105168	2.8750469	0.4751955
	-3	0.0901443	6.6765046	11.494861	18.730904	1.0127964	0.0403755	0.0170666	10.864418	8.6717412	13.311368	18.388673	1.9376251	8.7635225
	-2	12.560442	2.4176182	1.4039109	0.812737	11.740196	6.247455	10.032927	0.4051788	0.407398	17.453805	12.559696	20.263821	3.6948167
	-1	0.2429118	7.6773934	14.215397	2.5356283	0.2363543	10.054306	6.0039193	4.7892699	3.8802885	4.9188495	6.8407538	6.5289411	32.075987
**140**	0	11.938252	0.172738	0.1997019	1.1485187	20.049394	0.0032151	0.0238008	9.8567205	11.337124	0.0020304	0.0454661	37.784075	7.4389635
	1	0.2414774	7.6695493	14.186119	2.5859716	0.2392344	10.035337	5.9920156	4.8037391	3.8901122	4.9032776	6.8388482	6.1792541	32.435064
	2	12.398516	2.5526998	1.4618596	0.8093139	11.677476	6.2635917	10.056588	0.4007093	0.4027942	17.444511	12.561545	19.785348	4.1850458
	3	0.0865104	6.6562724	11.423909	18.818855	1.0176785	0.0422399	0.018362	10.85419	8.6603167	13.31842	18.394106	1.5717708	9.1373685
	4	13.568702	9.0612358	5.2658682	0.136342	0.1166339	6.5567527	10.397116	14.763852	17.889954	10.973302	7.9165984	2.7203786	0.633264
	5	0.2389345	4.5174321	5.3613364	26.458712	0.917885	21.57422	11.97345	11.94233	9.7897082	2.7961567	3.7444101	0.1036505	0.5817744
	6	16.959385	19.78567	12.255171	0.6907304	26.012877	5.5384337	11.5645	2.3170405	3.7112834	0.5603363	0.5322416	0.0563439	0.0159883
	-6	22.434592	32.954133	0.024026	0.8054019	21.162376	6.770756	8.7098222	3.2083993	2.6670275	0.8054638	0.3796509	0.0668926	0.0114589
	-5	0.0341761	0.0353752	10.266709	25.929766	0.8888752	16.739929	15.770408	8.2572838	13.987787	2.2404826	5.0764457	0.4556677	0.3170941
	-4	13.308997	12.929257	0.1800641	0.3148521	0.2941122	8.8998084	8.0281684	20.910725	11.288797	15.357557	4.7847452	3.0432158	0.6597017
	-3	0.3720643	0.164725	17.297078	18.275391	1.7734364	0.560324	0.7694283	6.2664604	11.925044	7.8740274	23.413297	5.6582947	5.6504309
	-2	9.4984182	3.1862584	1.4767646	1.3013512	12.957514	9.1186481	7.7995885	1.1743966	1.4636532	21.130249	6.6597183	17.895322	6.3381176
	-1	0.8999973	0.1029822	19.706915	2.6015678	2.3019399	7.3429208	8.2858396	3.8493479	5.6147713	2.7376445	8.6472528	15.149574	22.759247
**130**	0	8.2099124	0.0007172	2.3013221	1.3957019	21.26963	1.1142169	1.2601925	12.658879	6.0696529	0.3187898	1.4460398	28.458265	15.49668
	1	0.8947525	0.1089382	19.672645	2.6478836	2.3009271	7.3278982	8.2739858	3.8390545	5.6473391	3.6168377	7.7516294	11.63769	26.280419
	2	9.3438428	3.3635931	1.4729342	1.3011335	12.90661	9.1423214	7.8105394	1.1741625	1.4570125	21.497074	6.2852842	13.358038	10.887454
	3	0.3654439	0.1718082	17.210693	18.357233	1.773994	0.5625443	0.771598	6.2733018	11.895349	7.2315671	24.069108	2.4604394	8.8569197
	4	12.940697	13.306796	0.1792244	0.3144671	0.2835476	8.8819918	8.0142142	20.914172	11.296074	14.556436	5.6055874	1.6694201	2.0373728
	5	0.0329632	0.0362218	10.187651	25.947827	0.8890515	16.752565	15.778524	8.2602144	14.013479	1.8826335	5.4450678	0.1166557	0.6571472
	6	21.664144	33.639196	0.0239736	0.8074245	21.197986	6.7860767	8.7276902	3.2136025	2.6740139	0.7512374	0.4361742	0.0305243	0.0479573
	-6	7.1772973	4.7886343	16.051696	5.2009893	23.50562	8.0540461	15.16087	6.4652588	6.2001366	4.2034182	2.6663391	0.5047881	0.020907
	-5	4.3239537	6.101923	2.397503	10.537057	4.0421695	11.897601	13.861924	5.3132113	17.525942	7.8768513	13.066406	2.6730162	0.3824428
	-4	9.1475587	7.6461357	6.8891914	4.8125894	4.3243386	7.5798288	7.4587873	15.38466	3.4087652	20.766282	3.9681204	8.175943	0.4378001
	-3	9.4112977	8.3138919	5.3350176	5.4800907	7.6957182	10.486093	3.3459496	12.516698	4.5183964	0.5800834	15.807704	12.869056	3.6400047
	-2	5.9445359	11.695951	7.7556036	6.7347585	6.5891203	6.4640219	1.4382501	0.9151601	17.56655	7.1906353	3.2014951	20.779481	3.7244373
	-1	13.639918	3.4819253	4.8284506	14.300864	3.5298574	4.589537	2.5801452	8.8865018	0.7423132	10.706083	2.2759064	16.481781	13.956718
**120**	0	2.6083961	14.147798	13.563644	5.9135041	0.5690782	1.8442618	12.307503	1.029367	0.0319432	0.2062989	15.115794	19.824408	12.838003
	1	13.43716	3.6855281	4.837359	14.272407	3.5326887	4.6150904	2.581587	8.8571404	0.7447995	11.525851	1.4667031	7.835067	22.60862
	2	5.6268657	12.011714	7.7410526	6.7212098	6.6166187	6.4757058	1.416304	0.9144127	17.565847	7.9972486	2.3990087	7.9191258	16.594887

	3	9.0100049	8.7031716	5.3361452	5.4672264	7.7024695	10.478424	3.3467911	12.542504	4.507482	0.1724744	16.211597	1.4059274	15.115783
	4	8.7372095	8.0298525	6.8907069	4.814091	4.3386463	7.5504711	7.4673145	15.376887	3.4260923	19.032925	5.711619	1.3605842	7.2636002
	5	4.0736066	6.3304585	2.3918628	10.529794	4.0347988	11.891439	13.855426	5.3167236	17.546678	6.1273515	14.841123	0.1145054	2.946234
	6	6.8621967	5.0630165	15.981768	5.2154203	23.518876	8.0734806	15.179149	6.4814754	6.2150555	3.6144975	3.2681848	0.0563164	0.470564

Table S10. CASSCF-SO energies (cm^{-1}) of the states in the ground multiplet for $[Yb\{N(SiH_3)_2\}_2]^+$ with the same core geometry as **2-Yb**.

$\mathbf{[Yb\{N(SiH_3)_2\}_2]^+}$
0.000
0.000
543.412
543.412
1033.451
1033.451
1786.097
1786.097

Table S11. CASSCF-SO calculated EPR data for $[Yb\{N(SiH_3)_2\}_2]^+$ with the same core geometry as **2-Yb**.

Complex	g_1	g_2	g_3
$\mathbf{[Yb\{N(SiH_3)_2\}_2]^+}$	7.46	0.53	0.29

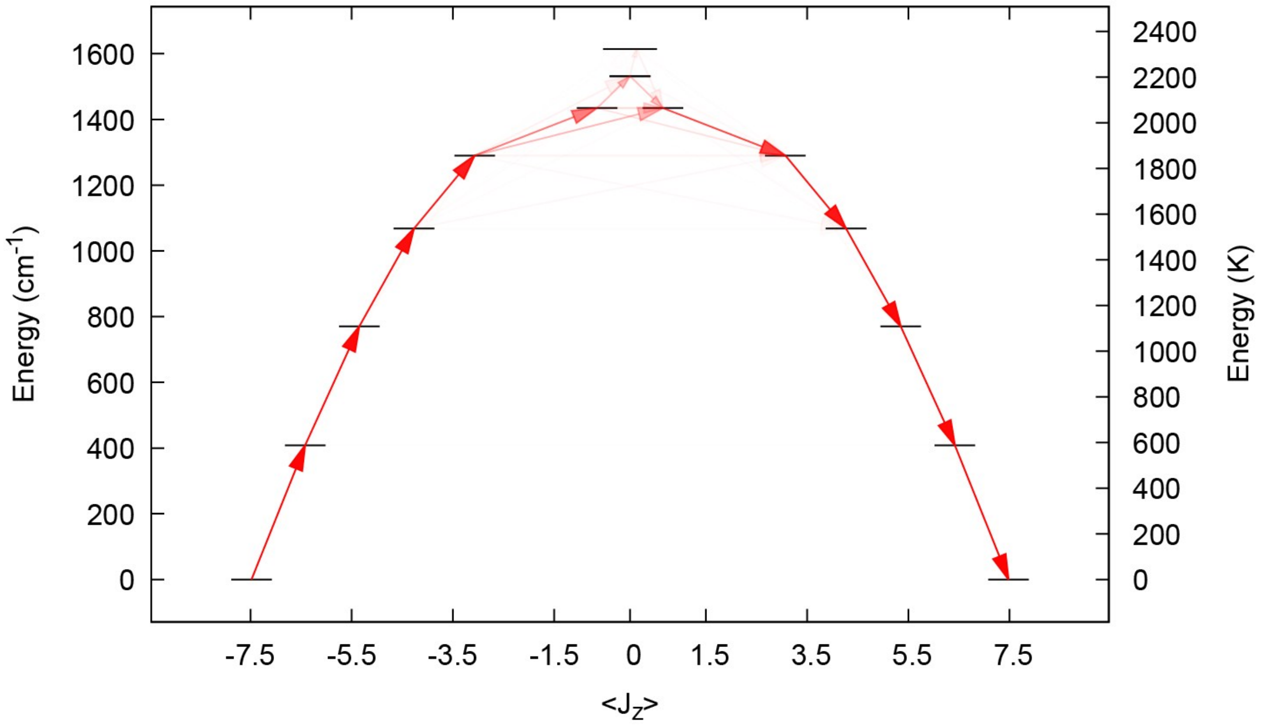

Figure S39. Barrier to magnetic relaxation for hypothetical $[Dy\{N(Si^{i}Pr_3)_2\}_2]^+$ complex calculated with CASSCF-SO using the geometry of **2-Tm**. Opacity of arrows is proportional to transition propensity calculated using the average of the three Cartesian magnetic moment operators.

Table S12. Electronic states for hypothetical $[Dy\{N(Si^{i}Pr_3)_2\}_2]^+$ cation calculated with CASSCF-SO using the geometry of **2-Tm**. CF wavefunctions given to nearest percent, with only contributions > 10% shown.

**Energy (cm^{-1})**	g_x	g_y	g_z	**Angle (°)**	**CF wavefunction**
0.0	0.00	0.00	19.83	--	98% $|\pm 15/2\rangle$
410.9	0.01	0.02	16.88	3.7	96% $|\pm 13/2\rangle$
769.0	0.16	0.17	13.97	5.7	92% $|\pm 11/2\rangle$
1065.9	0.75	0.91	11.10	6.2	74% $|\pm 9/2\rangle$ + 11% $|\mp 9/2\rangle$
1290.0	0.94	2.35	8.37	10.2	74% $|\pm 7/2\rangle$ + 14% $|\pm 3/2\rangle$
1436.2	3.95	4.73	7.81	73.4	56% $|\pm 5/2\rangle$ + 20% $|\pm 1/2\rangle$
1533.5	1.75	3.65	13.99	89.4	43% $|\pm 3/2\rangle$ + 15% $|\mp 1/2\rangle$ + 15% $|\mp 5/2\rangle$ + 12% $|\pm 7$
1615.1	0.29	0.76	19.12	88.3	34% $|\pm 1/2\rangle$ + 20% $|\pm 3/2\rangle$ + 18% $|\mp 1/2\rangle$ + 10% $|\pm 5$

7. DFT optimisation

We have optimised the geometry of the cation in **2-Yb** starting from the X-Ray geometry in the gas phase, using the Gaussian09d[13] suite of programs with the M06 functional.[14] The 28 inner electrons of Yb were described with the 1997 double-zeta Stuttgart effective core potential and the remaining valence electrons were described with the corresponding valence basis set.[15] All other atoms were described by the cc-pVDZ basis.[16,17]

Table S13. Optimized geometry of **2-Yb**.

Atom	x (Å)	y (Å)	z (Å)
Yb	0.131338	-0.8685	0.103806
N	2.190535	0.088553	-0.06097
N	-2.15504	0.014476	-0.14773
Si	3.154206	-0.97678	-1.02434
Si	2.428766	1.17422	1.269227
Si	-3.28512	-0.99303	0.821547
Si	-2.37523	1.407046	-1.26443
C	1.997664	-2.55265	-1.35424
C	1.005758	0.631908	2.512168
C	1.163407	-0.80291	3.017362
C	0.742765	1.568047	3.689351
C	4.14354	1.062002	2.108938
C	5.189659	1.783445	1.256804
C	4.239237	1.531895	3.558067
C	1.936038	2.976921	0.820709
C	2.261206	3.353637	-0.61988
C	2.412696	4.071768	1.772586
C	-2.15213	-2.41358	1.471132
C	0.825625	-2.37296	-2.32648
C	-1.60278	-3.32416	0.366896
C	-2.77146	-3.27812	2.569731
C	-4.73739	-1.64042	-0.23474
C	-5.53541	-2.74016	0.466264
C	-4.33377	-2.11895	-1.62706
C	-3.83487	0.117877	2.285855

C	-5.19368	-0.23862	2.880373
C	-2.77256	0.214929	3.376928
C	-1.13467	0.985515	-2.67369
C	-1.62614	-0.15621	-3.55829
C	2.783465	-3.76424	-1.85766
C	-0.68592	2.173104	-3.52159
C	-1.63726	2.921114	-0.33977
C	-1.95711	3.030152	1.144837
C	-1.95609	4.243043	-1.03506
C	-4.1684	1.63819	-1.86172
C	-5.08567	2.178144	-0.76557
C	-4.27389	2.509009	-3.11443
C	4.718681	-1.62935	-0.13429
C	4.384183	-2.5009	1.074896
C	5.780284	-2.29779	-1.00617
C	3.653255	-0.23888	-2.71633
C	2.492317	0.377844	-3.49127
C	4.730442	0.827152	-2.5162
H	1.602554	-2.86362	-0.34819
H	0.060044	0.708208	1.903417
H	1.376871	-1.55394	2.223711
H	0.292	-1.1556	3.602946
H	2.039402	-0.87705	3.685498
H	0.464402	2.57954	3.35392
H	1.633718	1.665395	4.329434
H	-0.07561	1.197381	4.333909
H	4.384776	-0.01819	2.096917
H	5.131272	1.523331	0.186427
H	6.213268	1.546648	1.597683
H	5.076559	2.878684	1.330583
H	3.93124	2.584724	3.677867
H	5.282052	1.465025	3.915999
H	3.628556	0.923426	4.245611
H	0.829054	2.933258	0.910038
H	3.350343	3.470402	-0.76642

H	1.797132	4.319067	-0.89754
H	1.919332	2.58689	-1.3355
H	1.950836	5.042555	1.51614
H	3.504285	4.218195	1.706047
H	2.175442	3.867503	2.829292
H	-1.30792	-1.8757	1.968719
H	-1.33902	-2.81402	-0.58336
H	-2.37074	-4.05464	0.061529
H	-0.73363	-3.92341	0.701407
H	-2.03727	-3.99875	2.96903
H	-3.61997	-3.86355	2.181193
H	-3.14292	-2.68283	3.417511
H	-5.39401	-0.75358	-0.34966
H	-4.96555	-3.68507	0.4965
H	-6.46162	-2.95355	-0.09399
H	-5.82761	-2.49109	1.497781
H	-3.66425	-2.99592	-1.5799
H	-3.82638	-1.34418	-2.2218
H	-5.22343	-2.43694	-2.19755
H	-3.91917	1.116767	1.816565
H	-5.46189	0.486408	3.668255
H	-5.18863	-1.23513	3.355485
H	-6.00563	-0.22256	2.135451
H	-1.76467	0.421882	2.979865
H	-2.71076	-0.71104	3.97411
H	-3.01286	1.030363	4.081437
H	-0.21662	0.651907	-2.12864
H	-2.53924	0.130868	-4.10852
H	-1.86944	-1.06996	-2.98733
H	-0.87035	-0.42947	-4.31652
H	0.209322	-1.45445	-2.22826
H	0.136175	-3.23916	-2.32236
H	1.214468	-2.29902	-3.3557
H	0.035139	1.839436	-4.2885
H	-0.18181	2.949848	-2.92414

H	-1.52848	2.644569	-4.05475
H	-0.5494	2.728252	-0.45287
H	-1.43928	3.902634	1.58386
H	-1.64731	2.14121	1.717833
H	-3.03703	3.183815	1.319052
H	-1.75792	4.237278	-2.11751
H	-1.34269	5.050291	-0.59551
H	-3.01142	4.530363	-0.88769
H	-4.51828	0.622476	-2.13216
H	-5.09577	1.551313	0.142659
H	-6.12739	2.242761	-1.12447
H	-4.79195	3.196846	-0.45743
H	-3.71064	2.098981	-3.968
H	-3.909	3.534268	-2.93496
H	-5.32673	2.595933	-3.43276
H	3.310942	-3.5197	-2.79637
H	2.113965	-4.61491	-2.07762
H	3.534642	-4.11349	-1.13667
H	5.168629	-0.6936	0.25206
H	3.619212	-2.04559	1.728582
H	5.27676	-2.68206	1.69997
H	4.008067	-3.49526	0.774663
H	5.443713	-3.26347	-1.41696
H	6.690182	-2.50753	-0.41652
H	6.086205	-1.67118	-1.85927
H	4.084885	-1.06604	-3.3164
H	1.745682	-0.36	-3.82722
H	2.851706	0.901212	-4.39553
H	1.965245	1.129878	-2.87476
H	4.325297	1.679428	-1.9411
H	5.077747	1.230439	-3.48338
H	5.618193	0.453828	-1.97951

8. References

1. *CrysAlis Pro*, Agilent Technologies: Yarnton, England, 2017.

2. G. M. Sheldrick, *Acta Crystallogr. Sect. C*, 2015, **71**, 3.

3. *POV-Ray*, Persistence of Vision Raytracer Pty. Ltd.: Williamstown, Australia, 2013.

4. G. M. Sheldrick, *Acta Crystallogr. Sect. A*, 2008, **64**, 112.

5. L. J. Farrugia, *J. Appl. Crystallogr*., 2012, **45**, 849.

6. O. V. Dolomanov, L. J. Bourhis, R. J. Gildea, J. A. K. Howard and H. Puschmann, *J. Appl. Crystallogr*., 2009, **42**, 339.

7. G. A. Bain and J. F. Berry, *J. Chem. Ed*., 2008, **85**, 532.

8. F. Aquilante, J. Autschbach, R. K. Carlson, L. F. Chibotaru, M. G. Delcey, L. De Vico, I. Fernandez Galván, N. Ferré, L. M. Frutos, L. Gagliardi, M. Garavelli, A. Giussani, C. E. Hoyer, G. Manni, H. Lischka, D. Ma, P. Å. Malmqvist, T. Müller, A. Nenov, M. Olivucci, T. B. Pedersen, D. Peng, F. Plasser, B. Pritchard, M. Reiher, I. Rivalta, I. Schapiro, J. Segarra-Martí, M. Stenrup, D. G. Truhlar, L. Ungur, A. Valentini, S. Vancoillie, V. Veryazov, V. P. Vysotskiy, O. Weingart, F. Zapata and R. Lindh, *J. Comput. Chem*., 2016, **37**, 506.

9. L. F. Chibotaru and L. Ungur, *J. Chem. Phys*., 2012, **137**, 064112.

10. B. O. Roos, R. Lindh, P.-Å. Malmqvist, V. Veryazov and P.-O. Widmark, *J. Phys. Chem. A*, 2005, **109**, 6575.

11. L. Ungur and L. F. Chibotaru, *Chem. Eur. J*., 2017, **23**, 3708.

12. N. F. Chilton, R. P. Anderson, D. L. Turner, A. Soncini, K. S. Murray, *J. Comput. Chem*. 2013, **34**, 1164.

13. *Gaussian 09*, Revision D.01, M. J. Frisch, G. W. Trucks, H. B. Schlegel, G. E. Scuseria, M. A. Robb, J. R. Cheeseman, G. Scalmani, V. Barone, B. Mennucci, G. A. Petersson, H. Nakatsuji, M. Caricato, X. Li, H. P. Hratchian, A. F. Izmaylov, J. Bloino, G. Zheng, J. L. Sonnenberg, M. Hada, M. Ehara, K. Toyota, R. Fukuda, J. Hasegawa, M. Ishida, T. Nakajima, Y. Honda, O. Kitao, H. Nakai, T. Vreven, J. A. Montgomery, Jr., J. E. Peralta, F. Ogliaro, M. Bearpark, J. J. Heyd, E. Brothers, K. N. Kudin, V. N. Staroverov, R. Kobayashi, J. Normand, K. Raghavachari, A. Rendell, J. C. Burant, S. S. Iyengar, J. Tomasi, M. Cossi, N. Rega, J. M. Millam, M. Klene, J. E. Knox, J. B. Cross, V. Bakken, C. Adamo, J. Jaramillo, R. Gomperts, R. E. Stratmann, O. Yazyev, A. J. Austin, R. Cammi, C. Pomelli, J. W. Ochterski, R. L. Martin, K. Morokuma, V. G. Zakrzewski, G. A. Voth, P. Salvador,

J. J. Dannenberg, S. Dapprich, A. D. Daniels, Ö. Farkas, J. B. Foresman, J. V. Ortiz, J. Cioslowski and D. J. Fox, Gaussian, Inc.: Wallingford CT, 2016.

14. Y. Zhao and D. G. Truhlar, *Theor. Chem. Acc.*, 2008, **120**, 215.

15. M. Dolg, H. Stoll, H. Preuss and R. M. Pitzer, *J. Phys. Chem.*, 1993, **97**, 5852.

16. T. H. Dunning, *J. Chem. Phys.*, 1989, **90**, 1007.

17. D. E. Woon and T. H. Dunning, *J. Chem. Phys.*, 1993, **98**, 1358.
